# A review of *Euryoryzomys legatus* (Rodentia, Sigmodontinae): morphological redescription, cytogenetics, and molecular phylogeny

**DOI:** 10.7717/peerj.9884

**Published:** 2020-10-29

**Authors:** Mariana D. Guilardi, Pablo Jayat, Marcelo Weksler, James L. Patton, Pablo Edmundo Ortiz, Keila Almeida, Maria José de J. Silva

**Affiliations:** 1 Laboratório de Ecologia e Evolução, Instituto Butantan, São Paulo, São Paulo, Brazil; 2 Unidad Ejecutora Lillo, CONICET- Fundación Miguel Lillo, San Miguel de Tucumán, Tucumán, Argentina; 3 Setor de Mastozoologia, Departamento de Vertebrados, Museu Nacional / Universidade Federal do Rio de Janeiro, Rio de Janeiro, Rio de Janeiro, Brazil; 4 Museum of Vertebrate Zoology, University of California, Berkeley, CA, United States of America; 5 Cátedra de Paleontología, Facultad de Ciencias Naturales and Instituto Miguel Lillo, Universidad Nacional de Tucumán, San Miguel de Tucumán, Tucumán, Argentina; 6 Superintendência da Polícia Técnico-Cientifica, Núcleo de Perícias em Crimes Contra Pessoa, Instituto de Criminalística, São Paulo, São Paulo, Brazil

**Keywords:** Neotropics, Rodents, Cricetidae, Oryzomyini, Karyotype, Morphology, Molecular systematics, Integrative taxonomy

## Abstract

The taxonomic history of *Euryoryzomys legatus* has been complex and controversial, being either included in the synonymy of other oryzomyine species or considered as a valid species, as in the most recent review of the genus. Previous phylogenetic analyses segregated *E. legatus* from *E. russatus*, its putative senior synonym, but recovered it nested within *E. nitidus*. A general lack of authoritative evaluation of morphological attributes, details of the chromosome complement, or other data types has hampered the ability to choose among alternative taxonomic hypotheses, and thus reach a general consensus for the status of the taxon. Herein we revisit the status of *E. legatus* using an integrated approach that includes: (1) a morphological review, especially centered on specimens from northwestern Argentina not examined previously, (2) comparative cytogenetics, and (3) phylogenetic reconstruction, using mitochondrial genes. *Euryoryzomys legatus* is morphologically and phylogenetically distinct from all other species-level taxa in the genus, but its 2n=80, FN=86 karyotype is shared with *E. emmonsae*, *E. nitidus*, and *E. russatus*. Several morphological and morphometric characters distinguish *E. legatus* from other species of *Euryoryzomys*, and we provide an amended diagnosis for the species. Morphological characters useful in distinguishing *E. legatus* from *E*. *nitidus,* its sister taxon following molecular analyses, include: larger overall size, dorsal fur with a strong yellowish brown to orange brown tinge, flanks and cheeks with an orange lateral line, ventral color grayish-white with pure white hairs present only on the chin, presence of a thin blackish eye-ring, tail bicolored, presence of an alisphenoid strut and a well-developed temporal and lambdoid crests in the skull, and a labial cingulum on M3. Molecular phylogenetic analyses recovered *E. legatus* as a monophyletic group with high support nested within a paraphyletic *E. nitidus*; genetic distances segregated members of both species, except for an exemplar of *E. nitidus*. Our integrated analyses reinforce *E. legatus* as a full species, but highlight that *E. macconnelli*, *E. emmonsae*, and *E. nitidus* each may be a species complex and worthy of systematic attention. Finally, we also evaluated the chromosome evolution of the genus within a phylogenetic context.

## Introduction

The genus *Oryzomys* Baird, 1857 was long recognized as a polyphyletic taxon with a complex taxonomy and wide distribution in the Neotropical region (Musser & Carleton, 2005). Weksler (2006) conducted a morphological and molecular phylogenetic review of the genus to delimit monophyletic units among the different species groups, which led to the erection of 10 new genera (Weksler, Percequillo & Voss, 2006). The *Oryzomys nitidus* group was allocated to the genus *Euryoryzomys* Weksler, Percequillo & Voss, 2006, which currently encompasses six recognized species (Percequillo, 2015): *E. emmonsae* (Musser et al., 1998), *E. lamia* (Thomas, 1901), *E. legatus* (Thomas, 1925), *E. macconnelli* (Thomas, 1910), *E. nitidus* (Thomas, 1884), and *E. russatus* (Wagner, 1848), plus *Euryoryzomys* sp., an additional lineage that is probably a new species (Silva, Percequillo & Yonenaga-Yassuda, 2000; Weksler, Percequillo & Voss, 2006). The species of *Euryoryzomys* are widely distributed through South America in lowland and lower montane tropical rainforest of greater Amazonia, Atlantic Forest, and Yungas, as well as in semi-deciduous forests, isolated patches of rainforest (“Brejos”) in the Caatinga, and gallery forests in the Cerrado and Chaco (Musser et al., 1998; Patton, Silva & Malcolm, 2000; Silva, Percequillo & Yonenaga-Yassuda, 2000; Mattevi & Andrades-Miranda, 2006; Weksler, Percequillo & Voss, 2006; Teta et al., 2007; Bonvicino, Oliveira & D’Andrea, 2008; Percequillo, 2015).

One species of *Euryoryzomys* with a complex taxonomic history is *E. legatus*. This taxon is distributed across premontane and montane forests along the eastern Andean slopes in central and southern Bolivia (Chuquisaca, Santa Cruz, and Tarija departments; the type locality is Caraparí, Tarija, Bolivia; Thomas, 1925) and northwestern Argentina (Jujuy and Salta provinces), at elevations ranging from 500 to 2,100 m (Anderson, 1997; Teta et al., 2007; Percequillo, 2015; Pardiñas & Ruelas, 2017). Sympatry with *E. nitidus* is observed in eastern Andean areas of central Bolivia (Percequillo, 2015). Since the original description by Thomas (1925) as *Oryzomys legatus*, the taxon was considered as a valid species by several authors (e.g., Gyldenstolpe, 1932; Tate, 1932; Ellerman, 1941) until, without any supporting evidence, Hershkovitz (1960) placed it as a synonym of *Oryzomys laticeps,* and Cabrera (1961) listed it as a subspecies of *O. capito*—both *capito* and *laticeps* are currently considered as junior synonyms of the oryzomyine *Hylaeamys megacephalus*; see Brennand, Langguth & Percequillo (2013). Massoia (1975) again considered *O. legatus* as a valid species but, shortly thereafter, Gardner & Patton (1976), followed by Honacki, Kinman & Koeppl (1982) and Redford & Eisenberg (1992), viewed this nominal form as a junior synonym of *O. nitidus*. Once again, the validity of *O. legatus* was sustained by Musser & Carleton (1993), but Musser et al. (1998) later subsumed it in the synonymy of *O. russatus*. Recent revisionary reports coincided in considering *legatus* as a valid species (Musser & Carleton, 2005; Barquez, Díaz & Ojeda, 2006; Weksler, Percequillo & Voss, 2006) and several authors, based on morphological and morphometric analyses, consistently differentiated *E. legatus* from *E. nitidus* and *E. russatus* (Weksler, 1996; Musser et al., 1998; Percequillo, 1998; Percequillo, 2015). Phylogenetic analyses based on the mitochondrial cytochrome-b gene (Patton, Silva & Malcolm, 2000) also supported the separation of *E. legatus* from *E. russatus*, but the former was recovered as nested within samples of *E. nitidus*, which led to uncertainty about the separation of both taxa.

Important sources for the taxonomic evidence for *E. legatus*, however, are still needed, such as cytogenetic data, more detailed molecular analyses including larger samples, and morphometric and morphological studies. This is especially true for populations from northwestern Argentina (NWA), which were underrepresented in previous revisionary studies (Musser et al., 1998). For these populations, morphological descriptions included small samples, with no molecular analysis. Most of what we know about this species in NWA includes meager notes about distribution and microhabitat preferences (Mares, Ojeda & Kosco, 1981; Jayat et al., 2006; Teta et al., 2007).

The aim of this study is to revisit the taxonomic status of *Euryoryzomys legatus* in an integrative taxonomic view. Based on this, we present the cytogenetic information (diploid and fundamental numbers) of *E. legatus* for the first time; examine morphological traits in a series of specimens from northwestern Argentina and southern Bolivia, comparing them with all other recognized species of the genus; and provide an expanded molecular phylogenetic analysis that includes mitochondrial genes of all currently recognized taxa of *Euryoryzomys,* in order to clarify the taxonomic status of this nominal form.

## Materials & Methods

### Studied Sample

**Figure 1:**
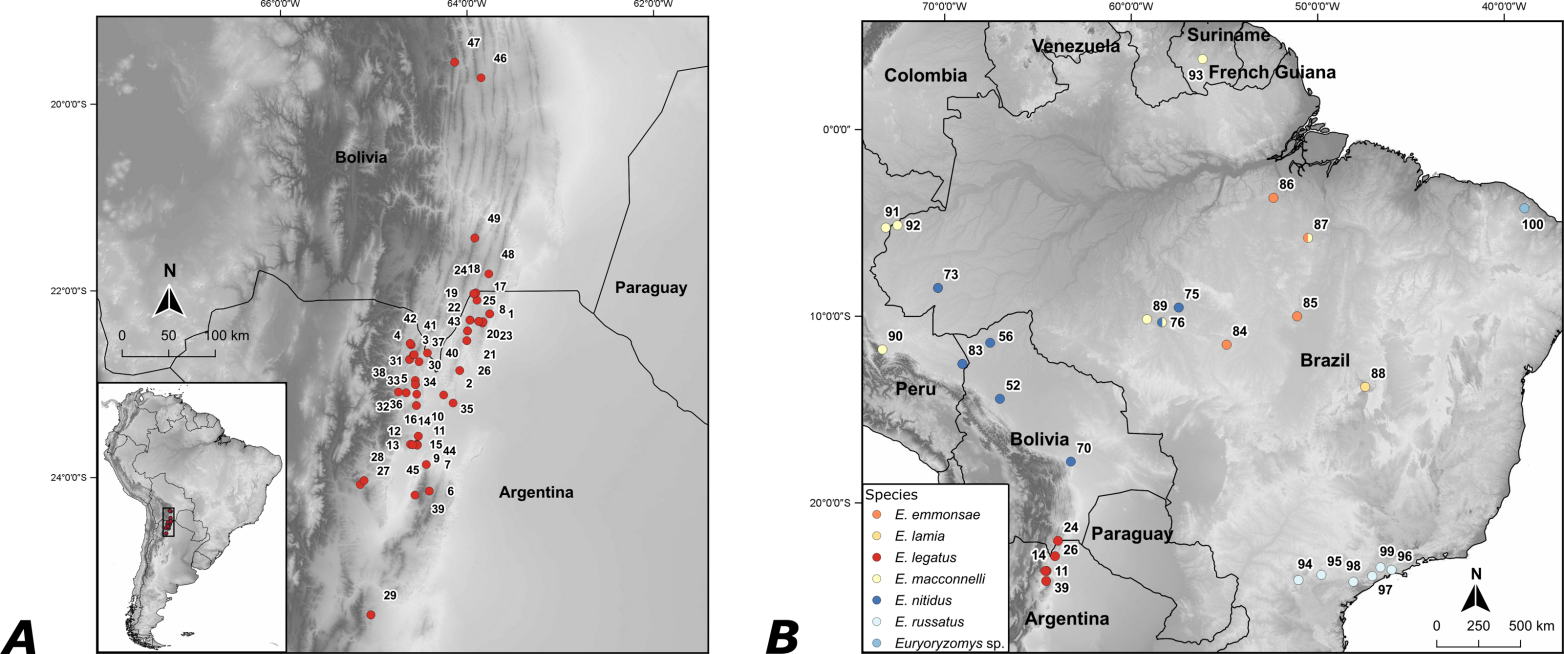
Map with the localities of *Euryoryzomys* samples (Table S1). (A) Localities numbered from 1 to 49 refer to the 145 specimens of *E. legatus* used in the morphological analyses. (B) Localities numbered from 11 to 100 refer to the *Euryoryzomys* species used in the phylogenetic analyses.

We studied the specimens of *E. legatus* based on three different approaches as follow: (i) 145 individuals were used for morphological descriptions and morphometric analyses (Fig. 1A, Table S1); (ii) two specimens out of the 145 were analyzed cytogenetically; and (iii) phylogenetic analyses were carried out using six out of those 145 individuals, in addition to the other *Euryoryzomys* species (Fig. 1B, Table S1). Tissues and chromosomal preparations were shipped under transportation permit CITES #19BR032169/DF.

We collected two specimens of *E. legatus* (CML13250 [field number JPJ2681] and CML13251 [field number JPJ2682]) with Sherman traps in Arroyo Yuto, 13 km SW Yuto, Jujuy, Argentina (Fig. 1A locality #11; Table S1), and deposited them in the Fundación Miguel Lillo collection, Tucumán, Argentina (CML). Collecting and shipping permits for these two specimens were issued by the Dirección de Recursos Genéticos y Protección de la Biodiversidad del Ministerio de Ambiente of Jujuy (#1102-122-2020/SByDS and #178/2020-SByDS). The capture and handling of the specimens followed the guidelines of the American Society of Mammalogists (Sikes et al., 2016) and with permission of the Instituto Butantan Ethics Committee (CEUAIB #1260/14).

We studied 134 additional specimens of *E. legatus* from Jujuy and Salta provinces, Argentina, and nine specimens from Chuquisaca and Tarija departments, Bolivia (Table S1). These specimens were housed in the following institutional collections: Colección Elio Massoia, Ciudad Autónoma de Buenos Aires, Argentina (CEM); Museo Argentino de Ciencias Naturales “Bernardino Rivadavia”, Ciudad Autónoma de Buenos Aires, Argentina (MACN-Ma); Fundación Miguel Lillo, Tucumán, Argentina (CML); Centro Regional de Investigaciones Científicas y Transferencia Tecnológica de La Rioja, La Rioja, Argentina (CRILAR-Ma); Museum of Southwestern Biology, University of New Mexico, New Mexico, USA (MSB); Natural History Museum, London, United Kingdom (BMNH). Analyzed material includes the holotype of *E. legatus* (BMNH 25.2.1.24).

Furthermore, we examined 1,215 representative specimens of other five species of *Euryoryzomys*, including 772 specimens of *E. russatus*, 24 specimens of *E. lamia*, 285 specimens of *E. macconnelli*, 9 specimens of *E. emmonsae*, and 125 specimens of *E. nitidus* (Table S1). These specimens were studied in the following collections: American Museum of Natural History, New York, USA (AMNH); Colección Elio Massoia, Ciudad Autónoma de Buenos Aires, Argentina (CEM); Colección Julio Contreras, Corrientes, Argentina (CJC); Fundación Miguel Lillo, Tucumán, Argentina (CML), Louisiana State University Museum of Zoology, Baton Rouge, Louisiana, USA (LSUMZ); Museo Argentino de Ciencias Naturales “Bernardino Rivadavia”, Ciudad Autónoma de Buenos Aires, Argentina (MACN-Ma); Museu de Biologia Mello Leitão, Santa Teresa, Brazil (MBML), Museu de História Natural Capão da Imbuia, Curitiba, Brazil (MHNCI); Museu Nacional do Rio de Janeiro, Universidade Federal do Rio de Janeiro, Rio de Janeiro, Brazil (MN); Museu Paraense Emílio Goeldi, Belém, Brazil (MPEG); Museum of Southwestern Biology, University of New Mexico, New Mexico, USA (MSB); Museum of Vertebrate Zoology, University of California, Berkeley, CA, USA (MVZ); Museu de Zoologia da Universidade de São Paulo, São Paulo, Brazil (MZUSP); The Museum, Texas Tech University, Lubbock, TX, USA (TTU); Laboratório de Mastozoologia da Universidade Federal de Minas Gerais, Belo Horizonte, Brazil (UFMG); Laboratório de Mastozoologia da Universidade Federal da Paraíba, João Pessoa, Brazil (UFPB); Laboratório de Citogenética de Mamíferos da Universidade Federal do Paraná, Curitiba, Brazil (UFPR); Laboratório de Mastozoologia da Universidade Federal de Santa Catarina, Florianópolis, Brazil (UFSC).

### Cytogenetic analyses

We obtained chromosomal preparations from one female (CML13250) and one male (CML13251) of *E*. *legatus*. Preparations were obtained *in vivo* from bone marrow and spleen, following the protocols of Ford & Hamerton (1956) and Yonenaga (1972), with modifications. Slides were Giemsa stained, and CBG and GTG-banding patterns were obtained according to Sumner (1972) and Seabright (1971), respectively, after modifications. Fluorescence *in situ* hybridization (FISH) with telomeric probes labeled with FITC was carried out following the recommended protocol (Telomere PNA FISH Kit/FITC, Code No. K5326, DAKO). Slides were counterstained with 4′,6-Diamidino-2′-phenylindole dihydrochloride (DAPI) in antifade mounting medium (Vectashield with DAPI, Vector).

We analyzed 72 metaphases from the male and 56 from the female specimen to establish both diploid (2n) and fundamental numbers (FN = number of arms of the autosomes). Metaphases were digitally captured in an Axioskop 40 epifluorescence microscope (Carl Zeiss) equipped with an Axiocam camera and AxionVision software. Adobe Photoshop CS5.1 was used for assembling the karyotypes.

### Phylogenetic and genetic distance analyses

Molecular data consisted of partial sequences from two mitochondrial genes, 801 bp of the cytochrome-b (cyt-b) and 667 bp of the cytochrome oxidase I (coxI; Table S1). In addition to newly generated sequences for 40 specimens, we also acquired sequences from seven individuals from GenBank (Table S1). DNA was extracted from liver or muscle using Chelex 5% (Walsh, Metzger & Higuchi, 1991). Cytochrome-b was amplified with primers MVZ05 and MVZ16 (Smith & Patton, 1993), and cytochrome oxidase I was amplified with primers LCO1490 and HCO2198 (Folmer et al., 1994). Polymerase chain reactions of 15 µL or 25 µL consisted of 30 ng of DNA, 10 mM of each primer, 0.2 mM of dNTP, reaction buffer (50 mM KCl, 2.5 mM MgCl2 and 10 mM Tris-HCl; ph 8.8) and 0.2 units of Platinum Taq DNA polymerase (Invitrogen). Amplification cycles were performed with an initial denaturation at 94 °C for 5 min; 30 cycles for cyt-b and 35 for coxI composed of: denaturation at 94 °C for 30 s, 45 s of annealing at 48 °C, and extension at 72 °C for 45 s; and a final cycle of extension at 72 °C for 5 min. Sequencing was performed at Laboratório de Bacteriologia II, Instituto Butantan, São Paulo, Brazil. We were unable to obtain sequences for both markers for some individuals (Table S1). The sequences were edited using Geneious R7 (Kearse et al., 2012) and deposited in GenBank (Table S1).

We used sequences from *Wiedomys cerradensis*, *Handleyomys, Nephelomys devius, Hylaeamys megacephalus, Oecomys auyantepui*, and *O. roberti* as outgroups in phylogenetic analyses (Table S2). Alignments using single genes and the two concatenate genes for all specimens were performed with MUSCLE (Edgar, 2004) using Geneious R7 (Kearse et al., 2012). The best evolutionary model for each analysis (cyt-b, coxI, and combined markers - cyt-b and coxI) was obtained using PartitionFinder (Lanfear et al., 2012). Bayesian inference (BI) was performed on MrBayes 3.2 (Ronquist & Huelsenbeck, 2003) and Maximum-Likelihood (ML) with bootstrap of 1000 replicates on RAxML-NG (Kozlov et al., 2019). The phylogenetic trees were edited with FigTree v1.4 (Rambaut & Drummond, 2009) and Adobe Illustrator CS4.

We employed MEGA7 (Kumar, Stecher & Tamura, 2015) to estimate cyt-b genetic distances (Kimura-2-parameter; Kimura, 1980) among all clades recovered in the phylogenetic analyses and among pairwise individuals of *Euryoryzomys*.

### Morphometric analyses

The following standard external measurements were recorded from field catalogs and tags: TL: total length; T: tail length; HF: hind foot length (including claw); E: ear length; and W: body mass. The following skull measurements were recorded with a vernier caliper to the nearest of 0.01 mm, following Hershkovitz (1962), Myers (1989) and Myers, Patton & Smith (1990): CIL: condyloincisive length; PB: palatal bridge; RL: rostral length; OL: orbital length; RW2: mid rostral width; ZP: zygomatic plate depth; IOC: interorbital constriction; ZB: zygomatic breadth; BB: braincase breadth; OCW: occipital condyle width; DL: diastema length; MTRL: maxillary toothrow length; IFL: incisive foramina length; AW1: alveolar width (across external side of both M1); BLLT: bullar length less tube; ML: mandible length. Five age classes were defined according to tooth wear following the descriptions in Jayat et al. (2020) and Fig. S1. Descriptive morphometric and univariate comparisons (Tukey’s pairwise comparison; Table 1) for samples of the species of *Euryoryzomys* were carried out with the software PAST (Hammer, Harper & Ryan, 2001). Specimens of age classes 2 and 3 (the largest pool of available specimens for the whole specimen sample) were pooled in tests of significant size differences (for both, *P* ≤ 0.05 and *P* ≤ 0.01).

**Table 1:** External and craniodental measurements for six species of *Euryoryzomys* (Supplementary Data 1) including age classes 2 and 3. * and ** indicate significant size differences (for both, *P* ≤ 0.05 and *P* ≤ 0.01 respectively) in the metric characters comparing all *Euryoryzmys* species with *E. legatus*.

	*E. emmonsae*			*E. lamia*			*E. legatus*			*E. macconnelli*			*E. nitidus*			*E. russatus*		
	*N*	X ± SD	r	*N*	X ± SD	r	*N*	X ± SD	r	*N*	X ± SD	r	*N*	X ± SD	r	*N*	X ± SD	r
TL	6	271 ± 20.23	247–301	5	302 ± 7.58	295–315	60	280 ± 21.26	205–315	103	252** ± 54.31	109–325	75	251** ± 50.16	128–314	393	283 ± 23.29	150–340
T	6	146 ± 14.69	125–168	6	149 ± 7.36	140–160	60	145 ± 10.35	118–165	90	140 ± 15.70	100–175	64	136** ± 12.04	105–161	397	148 ± 13.56	106-185
HF	6	34 ± 1.21	32–35	7	34 ± 1.50	33–37	62	34 ± 2.33	30–45	97	34 ± 1.91	29–38	78	33* ± 2.17	26–39	416	35** ± 2.22	26–41
E	6	22 ± 1.38	19–23	6	21* ± 1.05	19–22	62	24 ± 1.49	18–27	73	20** ± 2.40	14–26	78	22** ± 2.01	16–27	421	21** ± 2.84	14–35
W	6	60 ± 6.77	50–67	6	90** ± 11.40	70–100	55	67 ± 11.86	37–88	67	65 ± 17.30	31–105	74	66 ± 14.31	34–102	324	70 ± 16.33	33–116
CIL	6	28.50** ± 1.16	26.49–29.58	14	36.62* ± 1.26	30.38–34.50	61	31.38 ± 1.40	27.33–33.70	166	29.78** ± 1.53	24.88–32.63	88	29.99** ± 1.40	26.55–32.98	473	30.68** ± 1.47	25.66–35.20
DL	6	8.22 ± 0.44	7.71–8.84	14	9.88** ± 0.63	8.81–10.91	64	8.68 ± 0.49	7.36–9.62	200	9.01** ± 0.63	6.28–10.50	87	8.81 ± 0.53	7.47–10.19	494	8.70 ± 0.53	6.97–10.33
PB	6	6.43** ± 0.21	6.21–6.68	14	7.57* ± 0.50	6.97–8.20	63	7.15 ± 0.33	6.24–7.81	177	7.47** ± 0.52	6.06-8.59	86	6.66** ± 0.46	5.66–7.52	494	6.62** ± 0.41	5.62–7.72
MTRL	6	4.77** ± 0.21	4.38–4.96	14	5.05 ± 0.17	4.77–5.29	64	5.18 ± 0.13	4.87-5.44	202	5.04** ± 0.17	4.67–5.50	88	4.91** ± 0.18	4.43–5.29	495	5.15 ± 0.19	4.51–5.77
BLLT	6	3.58** ± 0.15	3.43–3.83	14	4.14 ± 0.16	3.81–4.34	61	4.17 ± 0.19	3.62–4.71	165	3.87** ± 0.23	3.25–4.89	87	3.88** ± 0.22	3.42–4.57	446	4.04** ± 0.28	3.11–5.21
IFL	6	5.21 ± 0.42	4.72–5.83	14	5.95** ± 0.27	5.27–6.42	64	5.15 ± 0.29	4.39–5.86	199	5.07 ± 0.39	3.98–5.94	87	5.55** ± 0.35	4.61–6.40	500	5.64** ± 0.40	4.37–6.75
AW1	6	5.73** ± 0.21	5.42–6.03	14	6.56 ± 0.24	6.20–6.88	64	6.31 ± 0.19	5.90–6.70	197	6.33 ± 0.32	5.09–7.11	87	6.08** ± 0.28	5.34–6.75	492	6.24 ± 0.31	5.17–7.07
ZB	6	15.68** ± 0.62	14.82–16.44	13	18.25 ± 0.50	17.42–18.91	63	17.74 ± 0.85	15.52–19.17	137	15.86** ± 0.71	14.03–17.33	83	16.50** ± 0.87	13.56–18.14	445	17.32** ± 0.82	14.86–19.45
ZP	6	3.59** ± 0.34	3.21–4.03	14	4.78 ± 0.35	4.18–5.65	64	4.52 ± 0.33	3.63–5.22	191	3.54** ± 0.31	2.48–4.45	88	4.18** ± 0.37	3.04–4.85	496	4.13** ± 0.37	2.94–5.12
BB	6	11.72** ± 0.38	11.27–12.17	14	12.97 ± 0.48	12.42–13.71	63	13.29 ± 0.60	11.89–14.25	189	12.19** ± 0.43	10.86–13.02	88	12.25** ± 0.41	11.31–13.13	481	12.79** ± 0.43	11.08–14.03
IOC	6	4.78** ± 0.23	4.53–5.07	14	5.72 ± 0.23	5.16–6.11	64	5.83 ± 0.23	5.43–6.51	202	5.22** ± 0.26	4.52–6.13	87	5.23** ± 0.22	4.82–5.78	502	5.47** ± 0.25	4.81–6.31
RW2	6	5.83** ± 0.41	5.11–6.32	14	6.86 ± 0.36	6.30–7.62	64	6.52 ± 0.38	5.68–7.38	199	6.16** ± 0.44	5.16–7.26	88	6.20** ± 0.43	5.20–7.11	500	6.28** ± 0.44	5.03–7.66
RL	6	12.44** ± 0.91	11.39–13.76	13	14.21 ± 0.75	13.03–15.42	62	13.68 ± 0.72	11.87–14.98	185	12.94** ± 0.74	10.48–14.62	88	12.78** ± 0.76	10.98–14.39	480	13.10** ± 0.81	10.19–15.44
OL	6	10.85** ± 0.36	10.22–11.26	14	12.16 ± 0.47	11.30–12.86	63	11.70 ± 0.51	10.36–12.57	190	10.76** ± 0.50	9.55–12.02	88	11.27** ± 0.55	9.11–12.72	494	11.46* ± 0.60	8.45–12.76
OCW	6	6.75** ± 0.24	6.53–7.10	14	7.09 ± 0.24	6.64–7.49	60	7.21 ± 0.22	6.78–7.71	155	7.15 ± 0.32	6.30–7.90	88	6.89** ± 0.27	6.24–7.66	470	7.29 ± 0.32	6.38–8.16
ML	6	17.02* ± 0.65	16.34–17.91	14	19.89** ± 0.87	18.26–21.35	63	18.13 ± 0.73	16.62–19.73	168	17.16** ± 0.90	14.42–19.07	87	17.75 ± 0.92	14.87– 19.71	487	18.29 ± 0.91	14.61–20.49

**Notes.**

N sample size.

X mean.

SD standard deviation,

r range.

**Table 2:** Results of the size PCA comparing six species of *Euryoryzomys* including age classes 2 and 3. Sample: *E. emmonsae*, *N* = 6; *E. lamia*, *N* = 12; *E. legatus*, *N* = 58; *E. macconnelli*, *N* = 72; *E. nitidus*, *N* = 77; and *E. russatus*, *N* = 359. Loadings of the variables, eigenvalues, and proportion of the variance explained for the first 3 principal components (PC). Results are based on log10-transformed craniodental variables. See “Materials & Methods” for variable abbreviations.

	Eigenvectors		
Variables	PC 1	PC 2	PC 3
CIL	0.2576	0.0773	−0.0786
DL	0.2618	0.2736	−0.3837
PB	0.1917	0.7229	−0.0132
MTRL	0.0907	0.0171	0.0972
BLLT	0.1537	−0.1312	0.3057
IFL	0.2404	−0.4534	−0.6482
AW1	0.1829	0.1468	−0.0237
ZB	0.2828	−0.1126	0.1085
ZP	0.4900	−0.3038	0.3588
BB	0.1627	−0.0387	0.1982
IOC	0.1557	0.0047	0.3472
RW2	0.3335	0.1298	−0.0308
RL	0.2984	0.1443	−0.0666
OL	0.2373	−0.0675	−0.0517
OCW	0.1234	0.0018	0.0410
ML	0.2495	−0.0546	−0.1113
Eigenvalue	0.0060	0.0016	0.0010
% of variance	50.5	13.7	8.4

**Table 3:** Results of the “size free” PCA comparing six species of *Euryoryzomys* including all age classes. Sample: *E. emmonsae*, *N* = 9; *E. lamia*, *N* = 18; *E. legatus*, *N* = 125; *E. macconnelli*, *N* = 102; *E. nitidus*, *N* = 109; and *E. russatus*, *N* = 476. Loadings of the variables, eigenvalues, and proportion of the variance explained for the first 3 principal components (PC). Results are based on Mosimann shape craniodental variables. See “Materials & Methods” for variable abbreviations.

	Eigenvectors		
Variables	PC 1	PC 2	PC 3
CIL	−0.1003	−0.0837	−0.053373
DL	−0.2237	−0.2936	−0.3602
PB	0.1061	−0.6997	−0.84624
MTRL	0.3475	0.0889	−0.023971
BLLT	0.2996	0.2353	0.13134
IFL	−0.2331	0.4634	−0.59612
AW1	0.1355	−0.0866	−0.095611
ZB	−0.0700	0.1231	0.13243
ZP	−0.5940	0.1555	0.57489
BB	0.2305	0.1084	0.1982
IOC	0.3063	0.1008	0.26222
RW2	−0.1289	−0.1455	0.076318
RL	−0.1759	−0.1820	−0.01441
OL	−0.0809	0.0705	−0.022391
OCW	0.2776	0.0870	−0.08782
ML	−0.0962	0.0579	−0.12142
Eigenvalue	0.0027	0.0015	0.0011
% of variance	34.3	19.3	14.8

**Table 4:** Results of the “size free” DFA comparing six species of *Euryoryzomys* including all age classes. Sample: *E. emmonsae*, *N* = 9; *E. lamia*, *N* = 18; *E. legatus*, *N* = 125; *E. macconnelli*, *N* = 102; *E. nitidus*, *N* = 109; and *E. russatus*, *N* = 476. Loadings of the variables, eigenvalues, and proportion of the variance explained for the first 4 discriminant functions (DF). Results are based on Mosimann shape craniodental variables. See “Materials & Methods” for variable abbreviations.

	Canonical Discriminant Functions			
Variables	DF 1	DF 2	DF 3	DF 4
CIL	0.0008	−0.0001	−0.2852	0.0006
DL	0.0067	−0.0054	−0.0067	0.0036
PB	0.0108	0.0082	−0.0022	0.0059
MTRL	0.0004	0.0005	0.0105	−0.0066
BLLT	−0.0021	0.0026	0.0032	−0.0020
IFL	−0.0031	−0.0156	−0.0034	−0.0031
AW1	0.0037	−0.0004	0.0032	−0.0017
ZB	−0.0043	0.0004	0.0007	0.0049
ZP	−0.0079	0.0021	−0.0179	−0.0008
BB	−0.0025	0.0054	0.0055	−0.0037
IOC	−0.0025	0.0063	0.0025	0.0012
RW2	0.0012	0.0020	−0.0020	−0.0006
RL	0.0017	0.0014	−0.0006	0.0049
OL	−0.0021	−0.0013	−0.0036	−0.0032
OCW	0.0005	−0.0010	0.0124	−0.0036
ML	−0.0012	−0.0051	−0.0016	0.0044
Eigenvalue	3.5206	1.5734	0.3503	0.0884
% of variance	63.1	28.2	6.3	1.6

With the aim of reducing the dimensionality of morphometric data in comparing *E. legatus* with other 5 species of *Euryoryzomys*, we conducted two Principal Components Analyses (PCA) using only skull measurements. The first PCA explored skull size variation (“size PCA” hereafter; Table 2) and we used only specimens of the age classes 2 and 3 (again taking advantage of the largest pool of available specimens). The second PCA was developed to explore skull shape differences (“size free PCA”; Table 3) and all age classes were used (after removing the size effect). This latter analysis was conducted using Mosimann shape variables (Mosimann & James, 1979) obtained as described in Meachen-Samuels and Van Valkenburgh (2009). Principal components (PC) and their statistical significance were obtained as in Jayat et al. (2020). Finally, we conducted a Discriminant Function Analysis using Mosimann shape variables and specimens of all age classes (“size free DFA”; Table 4). All multivariate statistical analyses were conducted in PAST (Hammer, Harper & Ryan, 2001) and using only the specimens without missing data.

### Morphological description

We re-described the skin color pattern and the skull of *E. legatus* and compared them with parapatric populations assigned to *E. nitidus* (its putative sister species) and other species of the genus. Terminology used to describe skull anatomical features followed Hill (1935), Carleton (1980), Voss (1988), Carleton & Musser (1989), Steppan (1995), and Weksler (2006). Descriptions of the molar cusp pattern followed Reig (1977).

## Results

### Cytogenetic analyses

**Figure 2:**
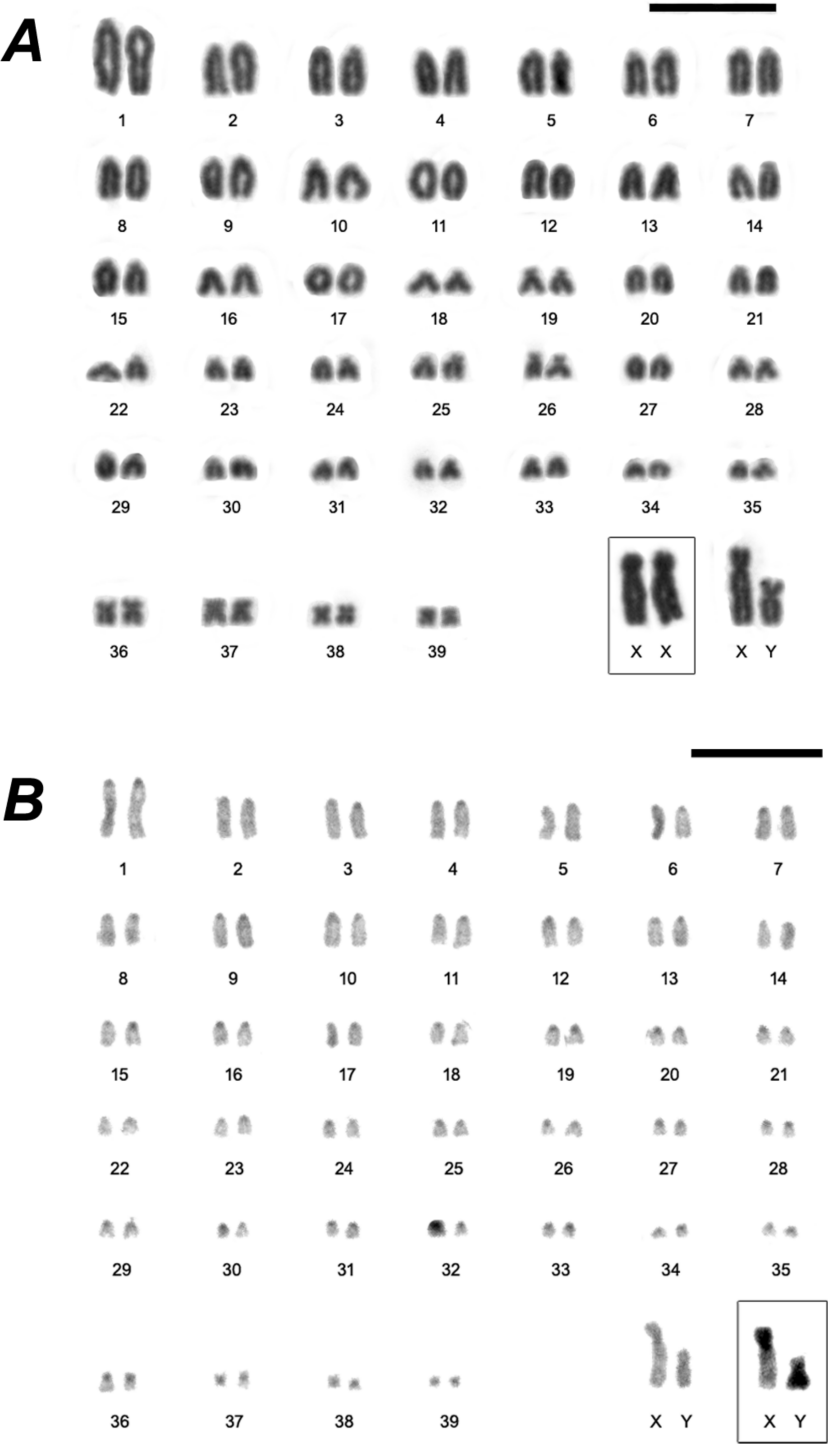
Karyotypes of a male of *Euryoryzomys legatus*, 2n=80, FN=86, from Arroyo Yuto, 13 Km SW Yuto, Jujuy, Argentina. (A) Conventional stained. Inset: sex chromosomes of a female. (B) C-band pattern. Inset: XY from another metaphase. Scale Bar = 10 µm.

Both karyotyped individuals presented 2n=80 and FN=86. The karyotype is composed of 35 acrocentric pairs, with pair 1 the largest of the complement and others pairs (2 to 35) decreasing gradually in size, and four small metacentric pairs (36 to 39). The sex chromosomes are readily distinguishable from the autosomes because of their different morphologies: the *X* is a large submetacentric and the *Y* is a medium-sized submetacentric (Fig. 2A).

**Figure 3:**
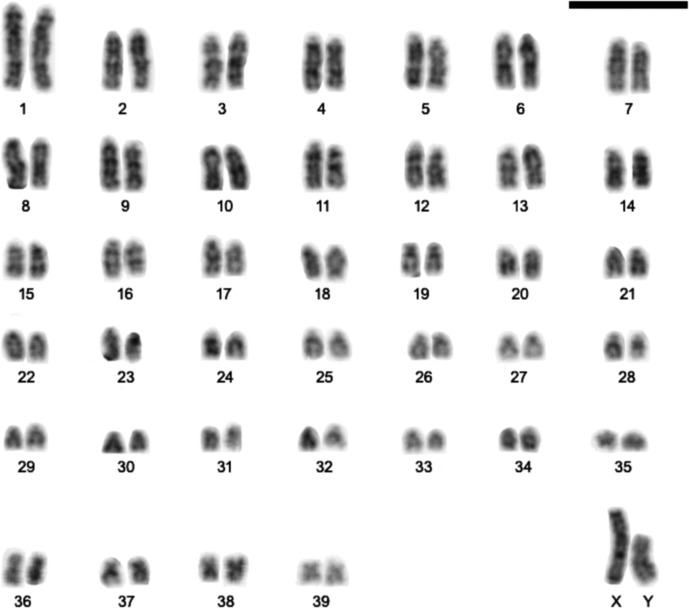
G-band pattern of a male of *Euryoryzomys legatus* with 2n=80, FN=86, from Arroyo Yuto, 13 Km SW Yuto, Jujuy, Argentina. Scale Bar = 10 µm.

**Figure 4:**
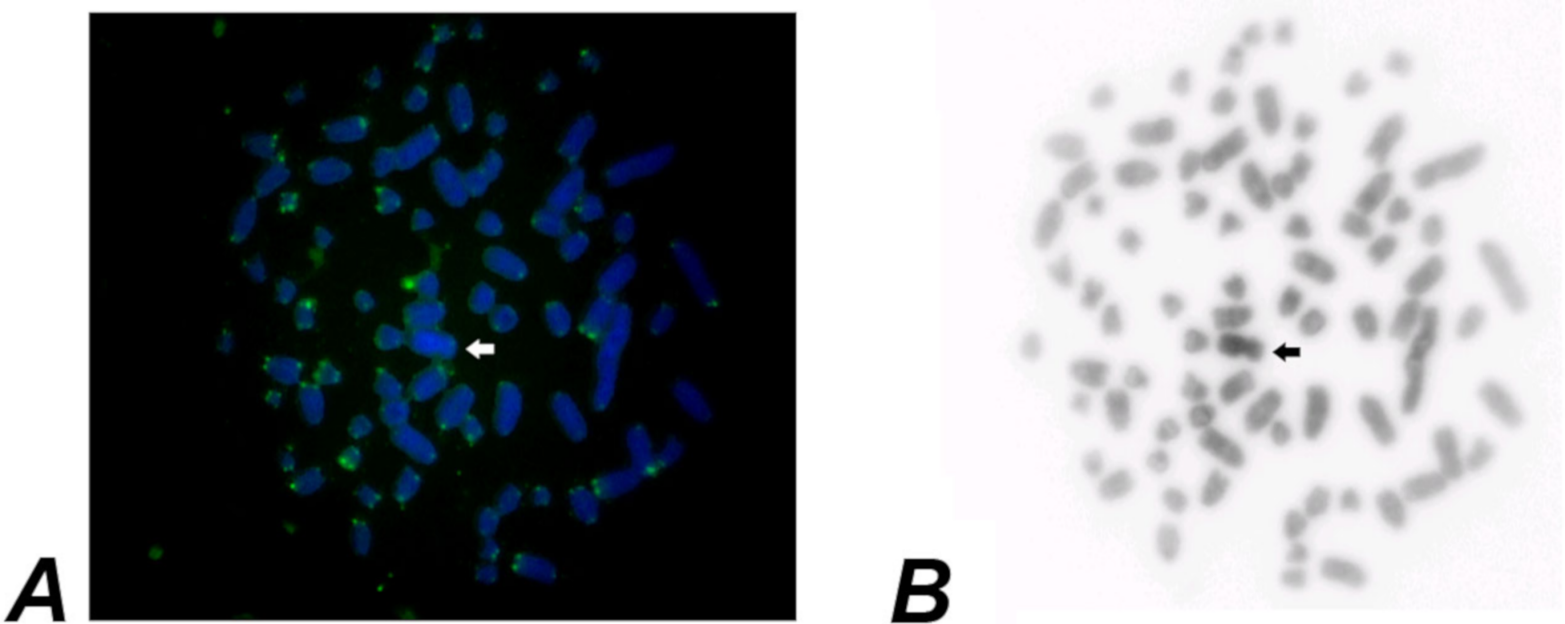
Metaphase of a male of *Euryoryzomys legatus* with 2n=80, FN=86, from Arroyo Yuto, 13 Km SW Yuto, Jujuy, Argentina. (A) After FISH with telomeric probes. (B) DAPI. Arrows indicate the Y chromosome.

CBG-bands revealed subtle pericentromeric constitutive heterochromatin in all autosomes. The X-chromosome is heterochromatic in the short arm, and the Y is entirely heterochromatic (Fig. 2B). GBG-bands allowed the identification of the homologues (Fig. 3). FISH showed signals exclusively at the telomeric regions of all chromosomes (Fig. 4A) and DAPI evinces the Y strongly and entirely stained (Fig. 4B).

### Phylogenetic analyses and genetic distances

**Figure 5:**
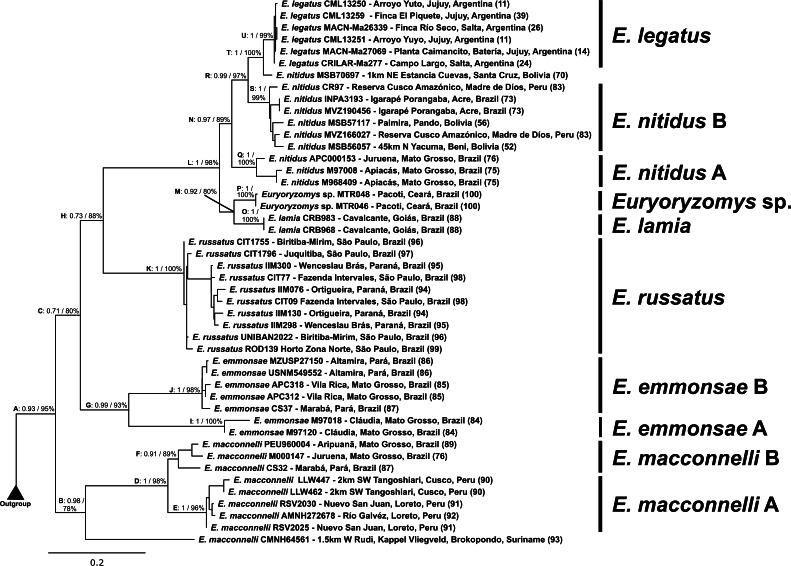
Phylogeny reconstructed with Maximum-Likelihood based on cyt-b partial sequences. The values on the nodes represent posterior probability (PP) using BI and ML bootstrap values, respectively.

The phylogenetic analyses of cyt-b provided high nodal support for the monophyly of *Euryoryzomys* (Clade A, BI: 0.93, ML: 95%) (Fig. 5). *E. macconnelli* (Clade B, BI: 0.98, ML: 78%) was recovered as the sister group to the clade containing all other species (Clade C, BI: 0.71, ML: 80%). *E. emmonsae* (Clade G, BI: 0.99, ML: 93%) was recovered as the sister group to a major subclade (Clade H, BI: 0.73, ML: 88%) composed of *E. russatus* (Clade K, BI: 1, ML: 100%), and the clade containing remaining species (Clade L, BI: 1, ML: 98%). This Clade L encompassed two subclades: Clade M (BI: 0.92, ML: 80%), with *E. lamia* (Clade O, BI: 1, ML: 100%) + *Euryoryzomys* sp. (Clade P, BI: 1, ML: 100%), and its sister clade (Clade N, BI: 0.97, ML: 89%), composed of *E. nitidus* and *E. legatus*. *E. nitidus* was recovered paraphyletic, splitting into *E. nitidus* A (Clade Q, BI: 1, ML: 100%) which is the sister group (Clade R, BI: 0.99, ML: 97%) of *E. nitidus* B (Clade S, BI: 1, ML: 99%) and its sister group (Clade T, BI: 1, ML: 100%), composed of *E. nitidus* MSB70697 and *E. legatus* (Clade U, BI: 1, ML: 99%).

For *E. macconnelli* (Clade B), the individual CMNH64561 from 1.5 km W Rudi, Brokopondo–Suriname (locality #93) was recovered as the sister to a clade (Clade D, BI: 1, ML: 98%) composed of *E. macconnelli* A (Clade E, BI: 1, ML: 96%) + *E. macconnelli* B (Clade F, BI: 0.91, ML: 89%). *E. macconnelli* A corresponds to individuals from Nuevo San Juan and Río Gálvez, Loreto Province–Peru (RSV2025, RSV2030 and AMNH272678–localities #91 and #92)–W Tangoshiari, Cusco Province–Peru (LLW447 and LLW462–locality #90); and *E. macconnelli* B is composed of specimens from Juruena and Aripuanã, Mato Grosso State (PEU 960004 and M000147–localities #76 and #89, respectively), and Marabá, Pará State–Brazil (CS32–locality #87).

*E. emmonsae* (Clade G) was also split into two major clades*, E. emmonsae* A from Cláudia, Mato Grosso State - Brazil (M97018 and M97120–locality #84) (Clade I, BI:1, ML: 100%); and *E. emmonsae* B from Altamira and Marabá, Pará State–Brazil (MZUSP27150, USNM549552 and CS37–localities #86 and #87) and Vila Rica, Mato Grosso State - Brazil (APC312, APC318–locality #85) (Clade J, BI:1, ML: 98%).

*E. nitidus* split into *E. nitidus* A (Clade Q), composed of individuals from Apiacás and Juruena, Mato Grosso State - Brazil (M968409, M97008 and APC153–localities #75 and #76); and *E. nitidus* B (Clade S), composed of individuals from Igarapé Porangaba, Acre State - Brazil (MVZ190456 and INPA3193 –locality #73), Reserva Cusco Amazónico, Cusco Province - Peru (CR97 and MVZ166027–locality #83), 45 km N Yacuma, Beni Department (MSB56057–locality #52) and Palmira, Pando Department - Bolivia (MSB57117–locality #56); and the individual *E. nitidus* MSB70697 from 1km NE Estancia Cuevas, Santa Cruz Department - Bolivia (locality #70) was recovered as sister to *E. legatus*.

**Figure 6:**
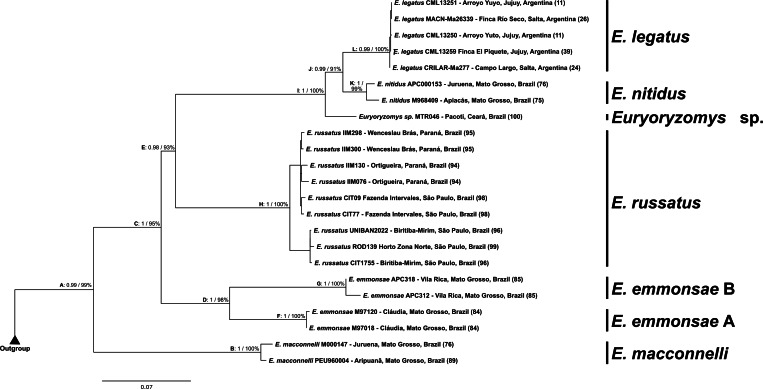
Consensus topology obtained in Maximum-Likelihood based on concatenate analyses using cyt-b and coxI sequences. The values on the nodes represent posterior probability (PP) using Bayesian Inference (BI) and bootstrap from the Maximum-Likelihood analysis (ML), respectively.

The phylogenetic analyses of the concatenated cyt-b and coxI markers (Fig. 6) showed *Euryoryzomys* as a monophyletic group (Clade A) with high support (BI: 0.99, ML: 99%), although *E. lamia* was not included in the analyses. The topology was similar to the cyt-b analyses, with *E. macconnelli* (Clade B) as the sister-group to all the other species (Clade C, BI: 1, ML: 95%), which encompasses *E. emmonsae* (Clade D, BI: 1, ML: 98%) and the sister group (Clade E, BI: 0.98, ML: 93%) composed of *E. russatus* (Clade H, BI: 1; ML: 100%), and its sister group (Clade I, BI: 1, ML: 100%) encompassing *Euryoryzomys* sp., and Clade J (BI: 0.99, ML: 91%) with *E. nitidus* (Clade K, BI: 1, ML: 99%) + *E. legatus* (Clade L, BI: 0.99, ML: 100%). *E. emmonsae* also was split into two subclades: *E. emmonsae* A from Claudia, Mato Grosso State - Brazil (M97018 and M97120–locality #84) (Clade F, BI:1, ML: 100%); and *E. emmonsae* B from Vila Rica, Mato Grosso State - Brazil (APC312 and APC318–locality #85) (Clade G, BI: 1, ML: 100%).

Genetic distances (K2p) using cyt-b sequences (Table S3) are: (i) 4.2% between *E. legatus* and *E. nitidus* B clades; (ii) 5.1% between *E. legatus* and *E. nitidus* A clades; (iii) 6.5% between *E. legatus* and *Euryoryzomys* sp. clades; (iv) 7.5% between *E. legatus* and *E. lamia* clades; (v) 16% between *E. legatus* and *E. russatus* clades; (vi) 14.2% between *E. legatus* and *E. emmonsae* B clades; (vii) 16% between *E. legatus* and *E. emmonsae* A clades; (viii) 15% between *E. legatus* and *E. macconnelli* B clades; and (ix) 14.7% between *E. legatus* and *E. macconnelli* A clades. In this analysis, we did not include the specimen *E. nitidus* MSB70697, since it is a single specimen and not a clade. However, the pairwise analysis of *Euryoryzomys* individuals presented that *E. nitidus* MSB70697 had lower genetic distance with *E. legatus* individuals (ranging from 1.7% to 1.9%) than to *E. nitidus* A (ranging from 4.8% to 5.7%) and *E. nitidus* B individuals (ranging from 3.6% to 4.4%) (Table S4).

### Morphological comparison of *E. legatus*

Externally, *E. legatus* specimens show all the character states reported by Thomas (1925) in the original description for the species. These specimens are similar to those of other species of *Euryoryzomys* in having a strong countershading between the dorsal and ventral pelage coloration. Nevertheless, individuals of *E. legatus* generally have dorsal fur with a strong yellowish brown to orange brown tinge, with clearer and brighter flanks and cheeks, forming an orange lateral line in most of the specimens examined; and ventral color grayish-white, with pure white hairs present only in small area on the chin. This more intense “ochraceous highlights” of the pelage, mentioned by Musser et al. (1998) as a difference between specimens of *E. legatus* and those of *E. nitidus*, may be also a useful character in separating specimens of *E. legatus* from those of *E. lamia*, which have a lighter dorsal coloration. This latter species also differs from *E. legatus* in ventral coloration, being more grayish cream than whitish. Individuals of *E. nitidus* possess unicolored tails (Musser et al., 1998; Percequillo, 2015), while in almost all the specimens of *E. legatus* the tail is certainly bicolored (dark brown above and grayish-white below). The presence of a thin blackish eye-ring in *E. legatus* was not described for other species of *Euryoryzomys*, so this character may be also useful in separating this form.

The skull of specimens of *E. legatus* follows the general form for the genus, with a long rostrum, narrow interorbital region with lateral margins divergent posteriorly, and the zygomatic arches widest at their squamosal roots. The mandible is deep and robust, with coronoid and condylar processes somewhat equal in height (separated by a shallow superior notch), and the angular process not extending posteriorly behind the condyloid process, also resembling the condition in other species of the genus. Qualitative characters of the skulls of *E. legatus* and other species the genus are very similar (see Table 13 in Weksler, 1996). Nevertheless, an alisphenoid strut, present in all specimens of *E. legatus* (including the holotype), is found in about half of previously examined specimens of *E. nitidus* (see Musser et al., 1998), and it is present in most, but not all, specimens of *E. lamia* and *E. russatus*, and completely absent in specimens of *E. macconnelli*. Additionally, we observed well-developed temporal and lambdoid crests in *E. legatus*, which were described as not well developed in *E. nitidus* (Weksler, 1996). Specimens of *E. legatus* with unworn molars always show a labial cingulum on M3, which is well developed in most of the examined specimens; in contrast, a labial cingulum is absent or vestigial in *E. lamia* and *E. nitidus* (Weksler, 1996).

### Morphometric analyses

Descriptive statistics for each of the species of *Euryoryzomys* are summarized in Table 1. *Euryoryzomys legatus* differed in several of the recorded metric characters from all other species of the genus. *Euryoryzomys lamia*, the most similar to *E. legatus* following the univariate comparison, showed significant differences in seven of the 21 morphometric characters studied. On the other extreme, *E. nitidus* was the most different species when compared to *E. legatus* (these species significatively differed in 18 of the 21 analysed measurements).

**Figure 7:**
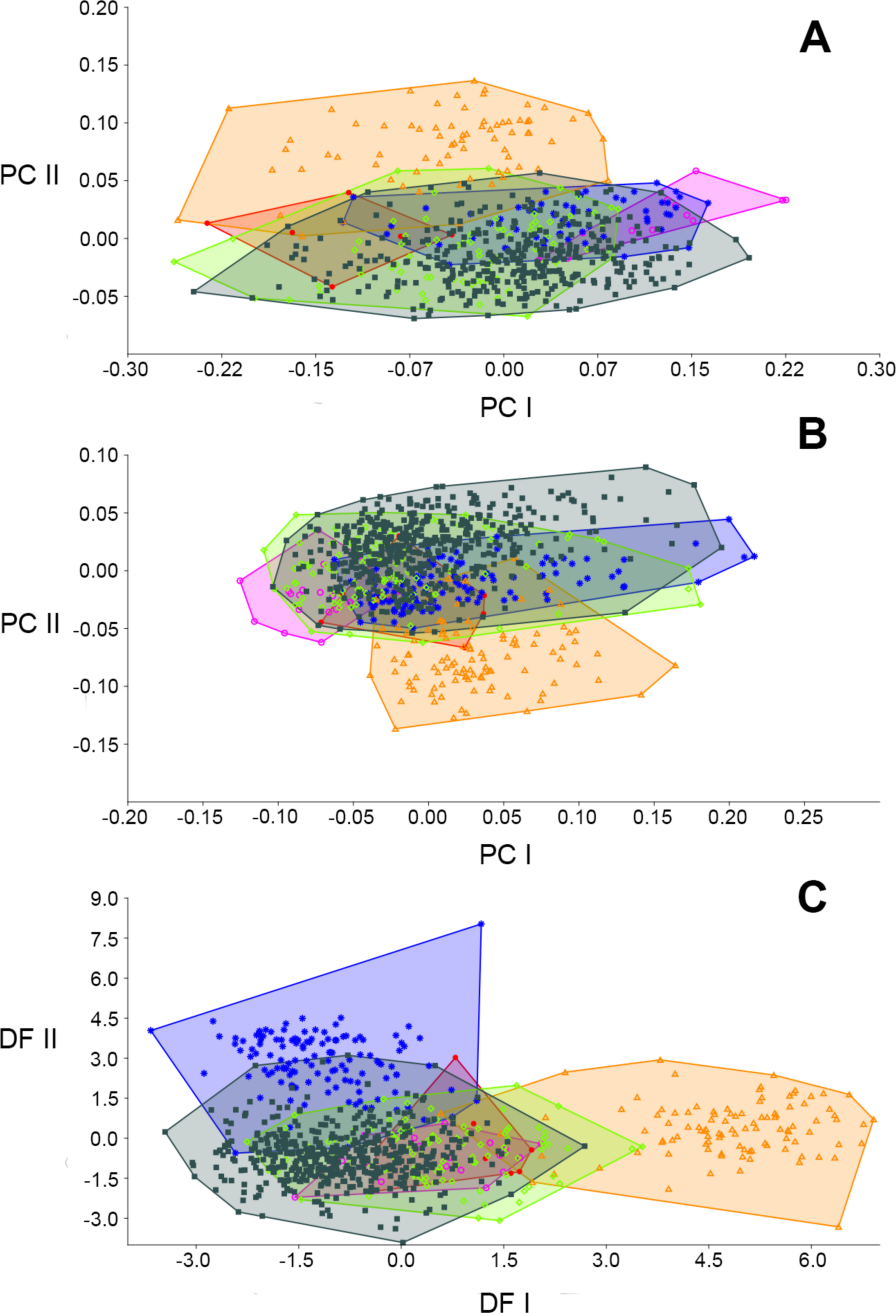
Graphic results of the PCA and DFA comparing six species of *Euryoryzomys*. (A) Individual specimen scores based on log-transformed values of 16 cranial measurements projected onto the first and second principal components of the “size” PCA. Character loadings and the variance explained by each of the first three principal components appear in Table 2. (B) Individual specimen scores based on log-transformed values of 16 cranial measurements (Mosimann shape variables) projected onto the first and second principal components of the “size-free” PCA. Character loadings and the variance explained by each of the first three principal components appear in Table 3. (C) Individual specimen scores based on log-transformed values of 16 cranial measurements (Mosimann shape variables) projected onto the first and second discriminant functions of the “size-free” DFA. Character loadings and the variance explained by each of the first four discriminant functions appear in Table 4. Red dots: *E. emmonsae*; fuchsia circles: *E. lamia*; blue asterisks: *E. legatus*; orange triangles: *E. macconnelli*; green diamonds: *E. nitidus*; black squares: *E. russatus*.

The first 3 principal components of the “size” PCA (Table 2) accounted for 50.48%, 13.70%, and 8.39% of the total variance, respectively, but only the first was judged statistically significant by the Broken-stick test. PC I was a size component, as all the variables had equal sign and loaded heavily on this axis (we confirm this by regressing PC I scores against several total length measure, e.g., CIL [slope = 17.957; intercept = 30.63; *r*^2^ = 0.877, *t* = 64.342, *p* = 9.3868E−267], and DL [slope = 5.238; intercept = 8.783; *r*^2^ = 0.515, *t* = 24.877, *p* = 1.3258E−93]). On bivariate plots of PC I and PC II, *E. legatus* mostly separated well from *E. macconnelli* and *E. emmonsae*, partially overlapped with *E. nitidus* and *E. lamia*, and widely overlapped with *E. russatus* in multivariate space (Fig. 7A). Specimens of *E. emmonsae* occupy negative values on PC I (being characterized by narrower zygomatic plates and smaller skulls) and those of *E. lamia* occupy positive values (broader zygomatic plates and larger skulls). The other four species widely spread over the PC I, but *E. macconnelli* and *E. nitidus* were mostly on the negative side. PC II mostly separated specimens of *E. macconnelli* from the other species, mostly by metric characters as the palatal bridge (longer in *E. macconnelli*) and the incisive foramina length (shorter in *E. macconnelli*).

**Figure 8:**
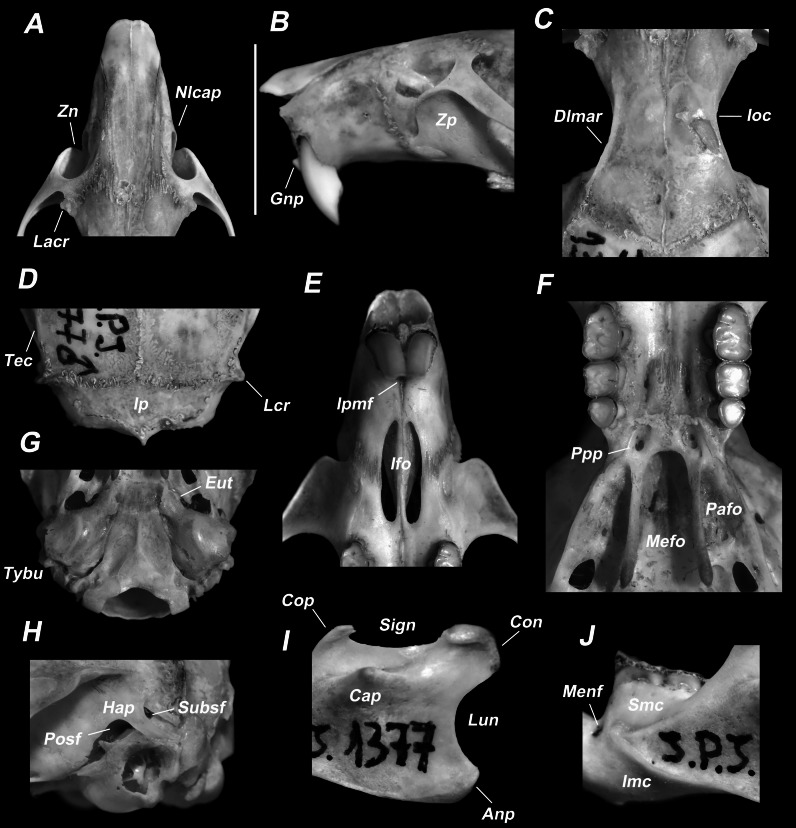
Qualitative morphological characters of several skull regions of *E. legatus* from NWA. (A) Dorsal view of the rostrum. (B) Lateral view of the rostrum. (C) Dorsal view of the interorbital region. (D) Dorsal view of the posterior skull. (E) Ventral view of the rostrum. (F) Ventral view of the palatal region. (G) Ventral view of the posterior skull. (H) Lateral view of the posterior skull. (I) Lateral view of the posterior mandible. (J) Lateral view of the middle mandible. Anp: angular process; Con: condyloid process; Cop: Coronoid process; DLmar: dorsolateral margins of the interorbital region; Gnp: gnathic process; Hap: Hamular process of the squamosal; Ioc: interorbital constriction; Ifo: Incisive foramina; Imc: inferior rami of the masseteric crest; Ip: Interparietal; Ipmf: interpremaxilary foramen; Lcr: lambdoidal crest; Lun: lunar notch; Mefo: Mesopterygoid fossa; Menf: mental foramen; Nlcap: nasolcrimal capsule; Pafo: parapterygoid fossa; Posf: Postglenoid foramen; Ppp: posteropalatal pit; Sign: sigmoid notch; Smc: superior rami of the masseteric crest; Subsf: subsquamosal foramen; Tec: temporal crest; Zn: zygomatic notch; Zp: zygomatic plate.

The “size free” PCA (Table 3) shows a similar general pattern to that observed in the “size” PCA, but *E. legatus* better separated from *E. lamia* and *E. macconnelli* (Fig. 8B). The first 3 principal components (all judged statistically significant according to the Broken Stick test) summarized 68.3% (PC I 34.3%, PC II 19.3%, and PC III 14.8%) of the explained variance. *E. lamia* occupied negative values on PC I (Fig. 7B), being the most specimens characterized by zygomatic plates (ZP) relatively broad and short molar series (MTRL). *E. legatus* mostly occupied the positive side of this PC (comparatively small ZP and large MTRL). PC II once again separated specimens of *E. macconnelli* from other species of *Euryoryzomys*, mostly by the palatal bridge (PB) and the incisive foramina length (IFL).

The “size free” DFA (Table 4) almost completely separated *E. legatus* from *E. macconnelli* and *E. lamia*, slightly overlapped *E. legatus* with *E. emmonsae* and *E. nitidus*, and partially overlapped *E. legatus* with *E. russatus* (Fig. 7C). DF I mostly segregated *E. macconnelli* from all other species on the basis of palatal bridge (PB) and diastema length (DL). DF II separated *E. legatus* from other species of *Euryoryzomys* except *E. russatus.* Incisive foramina length (IFL), interorbital constriction (IOC), and braincase breadth (BB) were important metric characters in this separation. The percentage of correct classifications following the jackknifed confusion matrix in this analysis was high (Table 5). Only 9.6% of the specimens of *E. legatus* were misclassified.

Given all the evidence, we consider *E. legatus* as a valid taxon and provide a taxonomic account and an emended diagnosis.

### Taxonomic Account

**Table:** 

***Euryoryzomys legatus* (Thomas, 1925)**
*Oryzomys legatus*Thomas, 1925:577.
*Oryzomys* (*Oryzomys*) *legatus*: Tate, 1932e:18; name combination.
*Oryzomys laticeps*: Hershkovitz, 1960:544, footnote; part; not *Mus laticeps* Lund (=*Hylaeamys laticeps* [Lund]).
*Oryzomys capito legatus*: Cabrera, 1961:386; name combination.
[*Euryoryzomys*] *legatus*: Weksler, Percequillo & Voss, 2006:11; first use of current name combination.

Holotype: adult male, BMNH 25. 2. 1. 24 (Original number 1777), collected 6th August, 1924.

Type locality: “Carapari [= Caraparí], 1000 m., about 35 kilometers north of Yacuiba, on the way towards Tarija,” Tarija, Bolivia.

Geographic Distribution: eastern Andean slope from southern Bolivia (Chuquisaca, Santa Cruz, and Tarija departments) to northernmost Argentina (Jujuy and Salta provinces), at elevations from 500 to 2,100 m.

Habitat: found in Subtropical mountain forest or Yungas, from the foothills in transition areas with chacoan habitats at 500 m, to the cloud forests in areas of contact with high altitude grasslands, at 2,100 m.

Emended diagnosis: *E. legatus* can be differentiated from the rest of the species of the genus by the following combinations of characters: intermediate size (external and skull measurements for specimens of age class 3: total length 205–315 mm, tail 118–159 mm, maximum skull length 32.45–37.36 mm, maxillary toothrow length 4.87–5.44 mm); skin with dorsum yellowish brown to orange brown; flanks and cheeks bright orange; belly predominantly whitish, with a small white spot (all white hairs) on the chin; eyes surrounded by a thin blackish ring; ears darker than dorsum; tail bicolored; rostrum long and robust; zygomatic arches slightly convergent anteriorly; alisphenoid strut present; capsular projection of the lower incisor forming a conspicuous expansion; presence of a labial cingulum on M3.

Morphological description: External measurements for adults (age classes 4 and 5) are: total length 281–340 mm; tail 132–161 mm; hindfoot 31–37 mm; ears 23–26 mm; body weight 57–102 g (Table 1).

Skin with dorsum yellowish brown to orange brown (Fig. S2); individual hairs plumbeous at base and orange at tip (underfur), or plumbeous at base and black at the tip (guard hairs). Flanks and cheeks clearer than dorsum, bright orange in most of the specimens examined. Belly strongly contrasting with the rest of the body, predominantly whitish, with hairs gray based and tipped whitish; most examined specimens possess a small white spot (all white hairs) on the chin. Eyes surrounded by a thin blackish ring. Ears darker than dorsum, internally and externally covered with brown hairs. Tail bicolored, dark brown above and grayish-white below, with scales evident without magnification. Hand and feet covered dorsally by whitish hairs, with a tuft of white hairs over the claws.

**Table 5:** Results of the jackknifed confusion matrix of the “size free” DFA comparing six species of *Euryoryzomys* including all age classes. Sample: *E. emmonsae*, *N* = 9; *E. lamia*, *N* = 18; *E. legatus*, *N* = 125; *E. macconnelli*, *N* = 102; *E. nitidus*, *N* = 109; and *E. russatus*, *N* = 476. Proportion of correct classifications = 75.5.

	*E. emmonsae*	*E. lamia*	*E. legatus*	*E. macconnelli*	*E. nitidus*	*E. russatus*	Total
*E. emmonsae*	5	0	1	0	1	2	9
*E. lamia*	1	15	0	0	0	2	18
*E. legatus*	1	1	113	0	5	5	125
*E. macconnelli*	6	1	0	94	1	0	102
*E. nitidus*	9	8	2	4	72	14	109
*E. russatus*	37	14	22	4	65	334	476
Total	59	39	138	102	144	357	839

Skull elongated and slightly compressed laterally (Figs. S3 and S4). Rostrum long and robust, nasals extending anteriorly well ahead of the anterior face of upper incisors and the gnathic process, and posteriorly not extending beyond the level of the lacrimals (Figs. 8A and 8B). Nasolacrimal capsules well developed, except in very young specimens. Zygomatic notches excavated, usually as wide as deep, but deeper than wider in some individuals. Lacrimals well visible, generally large and pointy laterally, except in very young and some adult specimens examined (Fig. 8A). Interorbital region posteriorly divergent, with dorsolateral margins beaded showing an overhanging shelf in most specimens examined (even vertically raised in some individuals; Fig. 8C). Frontoparietal suture predominantly U-shaped (Fig. 8C). Interparietal large, approximately 2.5 to 3 times wider than long (Fig. 8D). Zygomatic arches slightly convergent anteriorly and not well expanded laterally. Zygomatic plates comparatively broad and with straight or slightly concave anterior margins (Figs. 8B and 8E). Braincase relatively small, with temporal and lambdoidal crests well visible in most adult specimens (age classes 3 to 5; Fig. 8D). Interpremaxillary foramen is small and rounded (Fig. 8E). Incisive foramina proportionally short, posteriorly not extending beyond the anterior face of the procingulum of M1 (Fig. 8E). Bony palate extending behind the level of the alveolus of M3, with large posteropalatal pits - located slightly ahead, or at the same line, with the anterior margin of the mesopterygoid fossa - and with small palatine excrescences (Fig. 8F). Mesopterygoid fossa with the roof completely ossified, approximately of the same width of the parapterygoid fossae, with a rounded anterior margin, and without a well-developed median spine (Fig. 8F), except for a few specimens examined which shows a small median spine. Parapterygoid fossae somewhat excavated, with straight or divergent backwards lateral margins (Fig. 8F). Tympanic bullae small and with large and flattened Eustachian tubes (Fig. 8G). Alisphenoid strut present in all the specimens examined. Hamular process of the squamosal large, slender in most of the specimens, and distally pointy (Fig. 8H). Postglenoid foramen larger than subsquamosal foramen (Fig. 8H). Carotid arterial supply follows the Patterns 1 of Voss (1988), with the common carotid artery bifurcated behind the auditory bulla to form the external and internal carotid arteries, and the stapedial artery split into infraorbital and supraorbital branches.

Coronoid process of the mandible approximately at the same level as the condyloid process (Fig. 8I), but slightly above or below in some specimens examined. Angular process generally do not extend backward beyond the condylar process. Capsular projection of the lower incisor forming a conspicuous expansion located generally just behind the base of the coronoid process. Sigmoid notch comparatively shallow and lunar notch not well excavated (Fig. 8I). Upper and lower rami of the masseteric crest converging anteriorly, the lower ramus more developed than the upper one, extended approximately to the same level of the anterior border of the m1 (or slightly behind), and ending at level of the mental foramen (or just above) (Fig. 8J).

**Figure 9:**
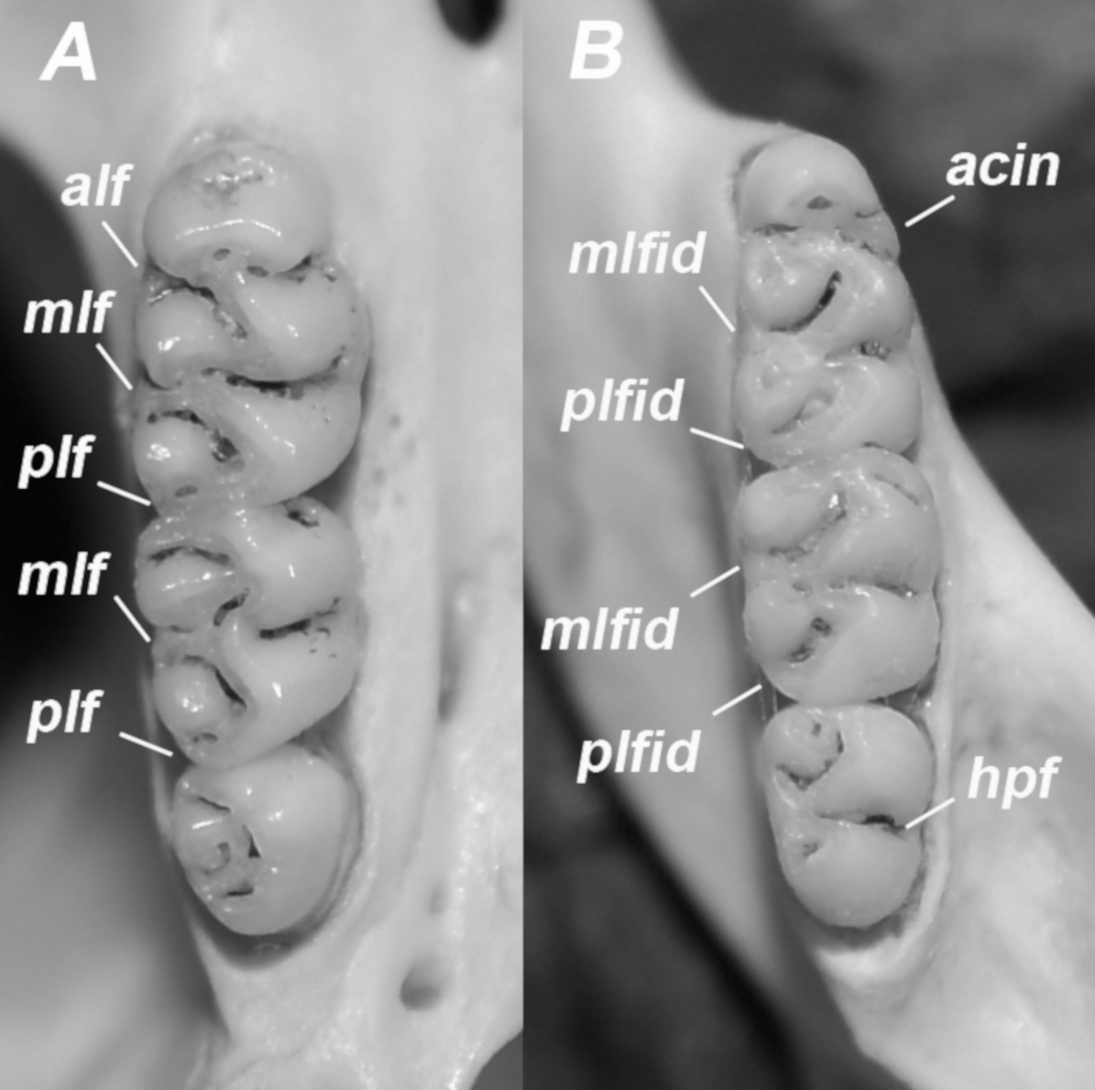
Molar structures in *E. legatus.* from NWA. (A) Occlusal view of the superior molar teeth. (B) Occlusal view of the inferior teeth. acin: anterolabial cingulid; alf: anteroloph; hpf: hypoflexid; mlf: mesoloph; mlfid: mesolophid; plf: posteroloph; plfid: posterolophid.

Upper incisors opisthodont (angular index lower than 90° sensu Thomas, 1919) and faced with orange enamel (Fig. 8B). Molars bunodont and pentalophodont (Fig. 9). M1 with procingulum without anteromedian flexus but with a depression on the base of its anterior face and with an enamel island at the level of the anteroloph; anteroloph, parastyle, and mesoloph well-developed, and posteroloph small (only visible in newly erupted molars) (Fig. 9A). M2 with a reduced procingulum but with mesoloph and posteroloph visible, and with an enamel island between protocone and paracone (just ahead of the median mure). M3 comparatively small, with the same general pattern (although with vestigial structures) as the M2 (Fig. 9A). Procingulum of the m1 without anteromedian flexid but with an enamel island; anterolabial cingulid, mesolophid, and posterolophid well developed (Fig. 9B). The m2 with a reduced procingulum, but mesolophid, and posterolophid distinct, and with a penetrating hypoflexid on the labial side. Although with less developed structures, the m3 shows the same general pattern that m2 (Fig. 9B).

## Discussion

Karyotypes are an important source of information for identification of some rodent species (Gardner & Patton, 1976; Musser et al., 1998; Patton, Silva & Malcolm, 2000; Andrades-Miranda et al., 2000; Di-Nizo et al., 2017). *Euryoryzomys* exhibits variation in diploid number from 2n=58 to 80, but the karyotypes are not species-specific, since different species share the same diploid number (*E. emmonsae*, *E. legatus*, *E. nitidus*, and *E. russatus* - 2n=80), and the same species can show different diploid numbers (*E. lamia* - 2n=58, 60, 64 and FN=84, and *E. macconnelli -* 2n=64, FN=70, 64; and 2n=76, FN=85) (Gardner & Patton, 1976; Bonvicino, Otazu & Weksler, 1998; Musser et al., 1998; Andrades-Miranda et al., 2000; Patton, Silva & Malcolm, 2000). In the case of *E. lamia*, only the cytogenetic information indicates the probable occurrence of cryptic species.

*Euryoryzomys legatus* showed the same diploid and fundamental numbers, as well as similar chromosome morphologies, to three other species of the genus (Musser et al., 1998; Andrades-Miranda et al., 2000). GTG-banding patterns of the karyotype of *E. legatus* showed similarities to those of *E. nitidus* and *E. russatus* (Silva, Percequillo & Yonenaga-Yassuda, 2000; Volobuev & Aniskin, 2000). Therefore, the karyotype is not informative to distinguish the four species with 2n=80, but it can be useful to distinguish them from *E. lamia*, *E. macconnelli*, and *Euryoryzomys* sp.

For *E. macconnelli*, considering only the diploid numbers, we can affirm the existence of cryptic species, given that specimens from Peru present 2n=64, FN=64 when compared to specimens from Venezuela that have a totally different diploid number (2n=76, FN=85); additionally, specimens from Brazil with 2n=64, FN=70, show at least one pericentric inversion leading to the different fundamental numbers (Gardner & Patton, 1976; Musser et al., 1998; Patton, Silva & Malcolm, 2000). Our phylogenetic analyses, although not being based on karyotyped specimens, also corroborates the split of *E. macconnelli* into three distinct lineages: *E. macconnelli* A from Brazil, *E. macconnelli* B from Peru, and the specimen CMNH64561 from Suriname*. E. emmonsae* was also recovered in two major clades. Previous studies indicated that *E. emmonsae* may also be a species complex (Costa, 2003; Percequillo, 2015), although the diploid number is the same for reported exemplars. A thorough review of these species is clearly necessary, one that would combine multiple character sets, much broader geographic sampling, and, for *E. macconnelli*, one that would include samples of all known cytotypes.

Regarding the chromosomal evolution of the genus, we associated the diploid and fundamental numbers into the phylogenetic tree. Independently of the diploid number of the outgroup, the split leading to the *E. macconnelli* clades (2n=76, FN=85;2n=64, FN=64 and 70) and the sister species clade (2n=80, FN=86;2n=76, FN=86;2n=58,60,64, FN=84), can be used to make the following inferences: (i) the difference between the FN=64 and 70 cytotypes in *E. macconnelli* is due to pericentric inversions involving biarmed chromosomes, given that the diploid number is the same; (ii) comparison between 2n=64, FN=70 and 2n=76, FN=85 forms led us to hypothesize that tandem fusions/fissions have occurred involving acrocentric chromosomes, and/or pericentric inversion and/or Robertsonian rearrangements in only one biarmed chromosome pair, since the former has four and the latter presents five biarmed chromosome pairs in the respective karyotypes; (iii) *Euryoryzomys* sp. and *E. lamia* underwent a reduction in the diploid numbers; (iv) the differences between the karyotypes of *Euryoryzoms* sp. and the four species with 2n=80 is due to Robertsonian rearrangements, as reported by Silva, Percequillo and Yonenaga (2000); and (v) karyotype differentiation of *E. lamia* involved complex rearrangements, since the three cytotypes showed an elevated number of biarmed chromosomes comparatively to the 2n=80 karyotypes.

Studies applying FISH techniques using species-specific probes (ZOO-FISH) showed a high number of chromosomal rearrangements even in the species with similar diploid numbers in *Cerradomys*, *Hylaeamys* and *Oligoryzomys*, three other oryzomyine genera (Nagamachi et al., 2013; Di-Nizo et al., 2015; Di-Nizo, Ferguson-Smith & Silva, in press). This could also be the case of *Euryoryzomys* species since herein we hypothesized - based on chromosome number variation, decrease (or increase) of the diploid numbers and phylogeny—the occurrence of complex and specific chromosome rearrangements. However, it is worth considering that only FISH with specific probes associated with differential chromosome staining will provide refined information on the chromosomal evolution of the genus.

Despite the various taxonomic changes since its original description, most authors today agree on the validity of *E. legatus* (e.g., Musser & Carleton, 1993; Musser & Carleton, 2005; Weksler, Percequillo & Voss, 2006; Percequillo, 2015; Pardiñas & Ruelas, 2017), and include it as a component of the mammal fauna of Argentina and Bolivia (e.g., Galliari, Pardiñas & Goin, 1996; Anderson, 1997; Salazar-Bravo et al., 2003; Barquez, Díaz & Ojeda, 2006; Teta et al., 2018). Nevertheless, several authors (including some of those who recognize it as a valid entity) emphasized the need for additional studies on the status of this species, especially with respect to *E. nitidus*. The lack of reciprocal monophyly in previous molecular studies among samples assigned to those nominal forms (in a context of a parapatric distribution) was the main source of uncertainty of its status.

Viewed in an integrated way, our morphometric analyses clearly separated *E. legatus* from other species of the genus. Univariate analysis shows several skull measurements that confidently distinguished this species from *E. nitidus*, *E. macconnelli*, and *E. russatus*, for which there are more than 13 metric characters that are significantly different. Even *E. lamia*, the most similar species according to the univariate analysis, shows seven measurements that significantly separated both species. The multivariate analyses also support the morphometric differentiation of *E. legatus*, not only in size (mainly from *E. macconnelli* and *E. emmonsae*), but also in shape of the skull (mainly from *E. macconnelli* and *E. lamia*, but also from *E. emmonsae* and *E. nitidus*).

The comparison between *E. legatus* and Bolivian populations assigned to *E. nitidus* deserves special attention, because of their close phylogenetic relationships. Musser et al. (1998), mostly based on specimens coming from Bolivia, differentiated *E. legatus* and *E. nitidus* on morphometric grounds. Results of our analyses, including samples of *E. legatus* from NW Argentina and southern Bolivia, confirm the distinctiveness between these two species (see also Figs. S5 and S6, and Tables S5–S8). Specimens of *E. legatus* were, on average, larger than *E. nitidus* for several measurements (Table 1). Total length (TL), tail length (T), hind foot length (HF), ear length (E), condyloincisive length (CIL), palatal bridge (PB), molar toothrow length (MTRL), bullar length less tube (BLLT), alveolar width (AW1), zygomatic breadth (ZB), zygomatic plate (ZP), braincase breadth (BB), interorbital constriction (IOC), mid rostral width (RW2), rostral length (RL), orbital length (OL), and occipital condyle width (OCW) are significantly larger in *E. legatus*. In contrast, *E. nitidus* appears significantly larger than *E. legatus* for the incisive foramina length (IFL). As revealed by the “size free” PCA and DFA, populations of both species also could be separated by the shape of the skull, because only 5% of the specimens of *E. legatus* were misclassified as *E. nitidus* and just 1.8% of the specimens of *E. nitidus* were misclassified as *E. legatus*. Finally, the chromatic differences in pelage corroborate the distinction of the two taxa.

In addition to the morphological evidence, molecular data also corroborate the separation between *E. legatus* and *E. nitidus*. Our phylogenetic results recover *E. nitidus* as paraphyletic, with genetic distance of 4.5% between the clades *E. nitidus* A and *E. nitidus* B, suggesting that two taxonomic entities can actually be considered: one from central Brazil (*E. nitidus* clade A) and another from western Amazon (*E. nitidus* clade B). The phylogenetic results point to the segregation of *E. legatus* from both *E. nitidus* A and *E. nitidus* B, and the genetic distances between *E. legatus* and these two clades are similarly high (*E. nitidus* A - 5.1% and *E. nitidus* B - 4.2%). Nevertheless, despite having expanded the number of specimens for each species, we did not have any *E. legatus* sample from the sympatric area in Bolivia to be compared. Additionally, our analyses lacked sequences from nuclear genes, because we had access only to cyt-b sequences for key samples (*E. nitidus* from Bolivia).

Peculiarly, the individual *E. nitidus* MSB70697 from southern Bolivia was recovered as sister to *E. legatus*. The collecting locality of MSB70697 (Bolivia: Santa Cruz de la Sierra: 1 km NE Estancia Cuevas; Anderson, 1997) is reported as exhibiting sympatry between *E. legatus* and *E. nitidus* (Musser et al., 1998). Although the specimen MSB70697 was identified by Musser et al. (1998) as *E. nitidus*, it was not included in their morphometric analyses (Musser et al., 1998: 212–213) from which the identification was inferred. We also noticed that this record came from the same type of habitat (montane Yungas forest) occupied by *E. legatus* (most additional records referred to *E. nitidus* in Bolivia are from a very different environment, the Chiquitano Forest, pertaining to the Amazonian Domain - see Anderson (1997) and Musser et al. (1998)). Therefore four hypotheses can be postulated for the recovered phylogenetic pattern: (i) MSB70697 is in fact an *E. legatus*, since this sample had higher similarity to *E. legatus* (genetic distance of 1.7% to 1.9%) than to *E. nitidus* (3.7% to 5.3%); (ii) each clade of *E. nitidus* is a different taxon (*E. nitidus* A, *E. nitidus* B, and the MSB70697), rendering *E*. *legatus* and each clade within the *E. nitidus* species complex as monophyletic; (iii) *E. nitidus* is indeed paraphyletic relative to *E. legatus*, which can be explained by a recent speciation event with incomplete lineage sorting; and (iv) *E. legatus* and *E. nitidus* are indeed the same taxon and thus the former is a junior synonym of the latter. If we integrate all the data obtained herein (morphological analysis, phylogeny, and genetic distance), we can consider one of the first three hypotheses and reject the last one. In this context, at this moment, we suggest maintaining the specific status of *E. legatus*.

It is clear that further studies investigating these hypotheses must be carried out. Looking at the problem in a broader perspective, these research lines altogether lead us to reinforce the need for a taxonomic revision of *Euryoryzomys* species based on integrative taxonomy, preferably including broader sampling and particularly sympatric areas, such as the region of Bolivia.

## Conclusions

This is the first study that describes the diploid number of *E. legatus* and integrates cytogenetics, morphology, and molecular phylogeny to infer the taxonomy and the evolutionary history of *Euryoryzomys*. Although *E. legatus* presents 2n=80, FN = 86, the same described for *E. emmonsae*, *E. nitidus*, and *E. russatus*, this karyotype is different from those of *E. lamia*, *E. macconnelli,* and *Euryoryzomys* sp. In addition, *E. emmonsae* and *E. russatus* show a disjointed distribution to *E. legatus*. The species has a close phylogenetic relationship to *E. nitidus*. We consider *E. legatus* as a valid species, due to the integration of phylogenetic information, genetic distances, and morphological data. Additionally, phylogenetic results pointed out that *E. emmonsae*, *E. macconnelli* and *E. nitidus*, as recognized nowadays, are probably species complexes, and the same may be the case for *E. lamia*, if we consider the cytogenetic data available.

##  Supplemental Information

10.7717/peerj.9884/supp-1 Supplemental Information 1 Tooth wear age classes in *E. legatus* from NWA Age class 1 to age class 5 (from left to right). See explanation of the tooth wear pattern in Material & Methods section.

10.7717/peerj.9884/supp-2 Supplemental Information 2 Skin color pattern in *E. legatus* from NWA Images show dorsum, flanks, belly, tail, and fore and hind feet. Details of the blackish eyering and the small white spot on the chin.

10.7717/peerj.9884/supp-3 Supplemental Information 3 Dorsal and ventral view of the skulls of *E. legatus* specimens from NWA Age class 1 to age class 5 (from left to right).

10.7717/peerj.9884/supp-4 Supplemental Information 4 Lateral view of the skulls and mandibles of *E. legatus* specimens from NWA Age class 1 to age class 5 (from top to bottom).

10.7717/peerj.9884/supp-5 Supplemental Information 5 Map with the localities of *Euryoryzomys legatus* and *E. nitidus* samples (see Table S1) Localities numbered from 1 to 82 refer to the *Euryoryzomys legatus* and *E. nitidus* included in the morphological analyses.

10.7717/peerj.9884/supp-6 Supplemental Information 6 Graphic results of the PCA and DFA comparing *Euryoryzomys legatus* (blue) and *E. nitidus* (green) (A) Individual specimen scores based on log-transformed values of 16 cranial measurements projected onto the first and second principal components of the “size” PCA. Character loadings and the variance explained by each of the first 3 principal components are in Table 1. (B) Individual specimen scores based on log-transformed values of 16 cranial measurements (Mosimann shape variables) projected onto the first and second principal components of the “size-free” PCA. Character loadings and the variance explained by each of the first 3 principal components appear in Table 2. (C) Frequency distribution of the specimen onto the first discriminant function of the “size-free” DFA. Character loadings and the variance explained by the DFA are in Table 3.

10.7717/peerj.9884/supp-7 Supplemental Information 7 Specimens of *Euryoryzomys* studied in this work. Localities and map numbers are as indicated in Fig. 1 5, 6, and S5 Taxon, voucher and field number are as indicated in Figures 5 and 6. *E. legatus*’** specimens used in the morphological analyses are indicated. GenBank accession numbers are also indicated. Samples in bold represent DNA sequences generated by this work. ****

10.7717/peerj.9884/supp-8 Supplemental Information 8 Sequences of the species used as the outgroup Taxon, gene, GenBank accession number, voucher/field number, and references are indicated.

10.7717/peerj.9884/supp-9 Supplemental Information 9 Genetic distances obtained by Kimura-2-parameter analyses of cyt-b sequences between *Euryozyomys* clades obtained in the phylogenetic analysis

10.7717/peerj.9884/supp-10 Supplemental Information 10 Genetic distances obtained by Kimura-2-parameter analyses of cyt-b sequences of pairwise *Euryoryzomys* individuals

10.7717/peerj.9884/supp-11 Supplemental Information 11 Results of the “size” PCA comparing two species of *Euryoryzomys* (*E. legatus*, *N* = 58 and *E. nitidus*, *N* = 77) including age classes 2 and 3 Loadings of the variables, eigenvalues, and proportion of the variance explained for the first 3 principal components (PC). Results are based on log10- transformed craniodental variables. See “Material & Methods” for variable abbreviations.

10.7717/peerj.9884/supp-12 Supplemental Information 12 Results of the “size free” PCA comparing two species of *Euryoryzomys* (*E. legatus*, *N* = 125 and *E. nitidus*, *N* = 109) including all age classes Loadings of the variables, eigenvalues, and proportion of the variance explained for the first 3 principal components (PC). Results are based on Mosimann shape craniodental variables. See “Material & Methods” for variable abbreviations.

10.7717/peerj.9884/supp-13 Supplemental Information 13 Results of the “size free” DFA comparing two species of *Euryoryzomys* (*E. legatus*, N=125; *E. nitidus*, N=109) including all age classes Loadings of the variables, eigenvalues, and proportion of the variance explained for the discriminant functions (DF). Results are based on Mosimann shape craniodental variables. See “Material & Methods” for variable abbreviations.

10.7717/peerj.9884/supp-14 Supplemental Information 14 Results of the jackknifed confusion matrix of the “size free” DFA comparing two species of *Euryoryzomys* (*E. legatus*, *N* = 125 and *E. nitidus*, *N* = 109) including all age classes Proportion of correct classifications = 95.7.

10.7717/peerj.9884/supp-15 Supplemental Information 15 Measurement of *Euryoryzomys’* individuals

## Additional Information and Declarations

### Field Study Permissions

The following information was supplied relating to field study approvals (i.e., approving body and any reference numbers): Field study approval #1102-122-2020/SByDS and specimens transportation approval #178/2020-SByDS by Dirección de Recursos Genéticos y Protección de la Biodiversidad del Ministerio de Ambiente of Jujuy. Tissues and cytogenetic preparations transportation approval #19BR032169/DF by IBAMA/CITES.

### DNA Deposition

The following information was supplied regarding the deposition of DNA sequences: The generated sequences are available at GenBank: MT118030–MT118031, MT118033–MT118034, MT118036–MT118042, MT118044–MT118052, MT118054–MT118096.

### Data Availability

The following information was supplied regarding data availability: Raw data are available in a Supplemental File, with species names, vouchers, measurements, and location of the specimens (collections).
