# Supplementary figures and images for: A review of Euryoryzomys legatus (Rodentia, Sigmodontinae): morphological redescription, cytogenetics, and molecular phylogeny
Source: PeerJ. 2020 Oct 29;8:e9884. doi: 10.7717/peerj.9884 (PMC7603791; doi:10.7717/peerj.9884)

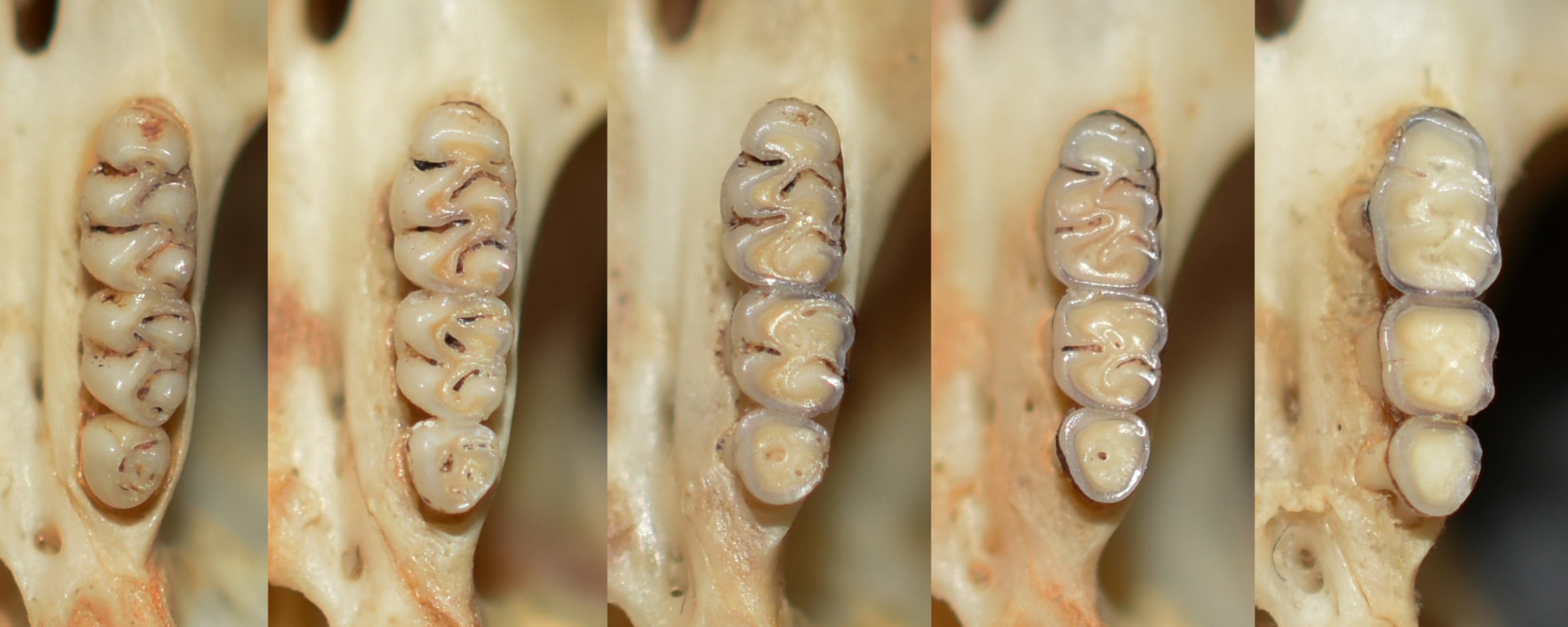

Supplement: Supplemental Information 1 — Age class 1 to age class 5 (from left to right). See explanation of the tooth wear pattern in Material & Methods section. [file peerj-08-9884-s001.png]

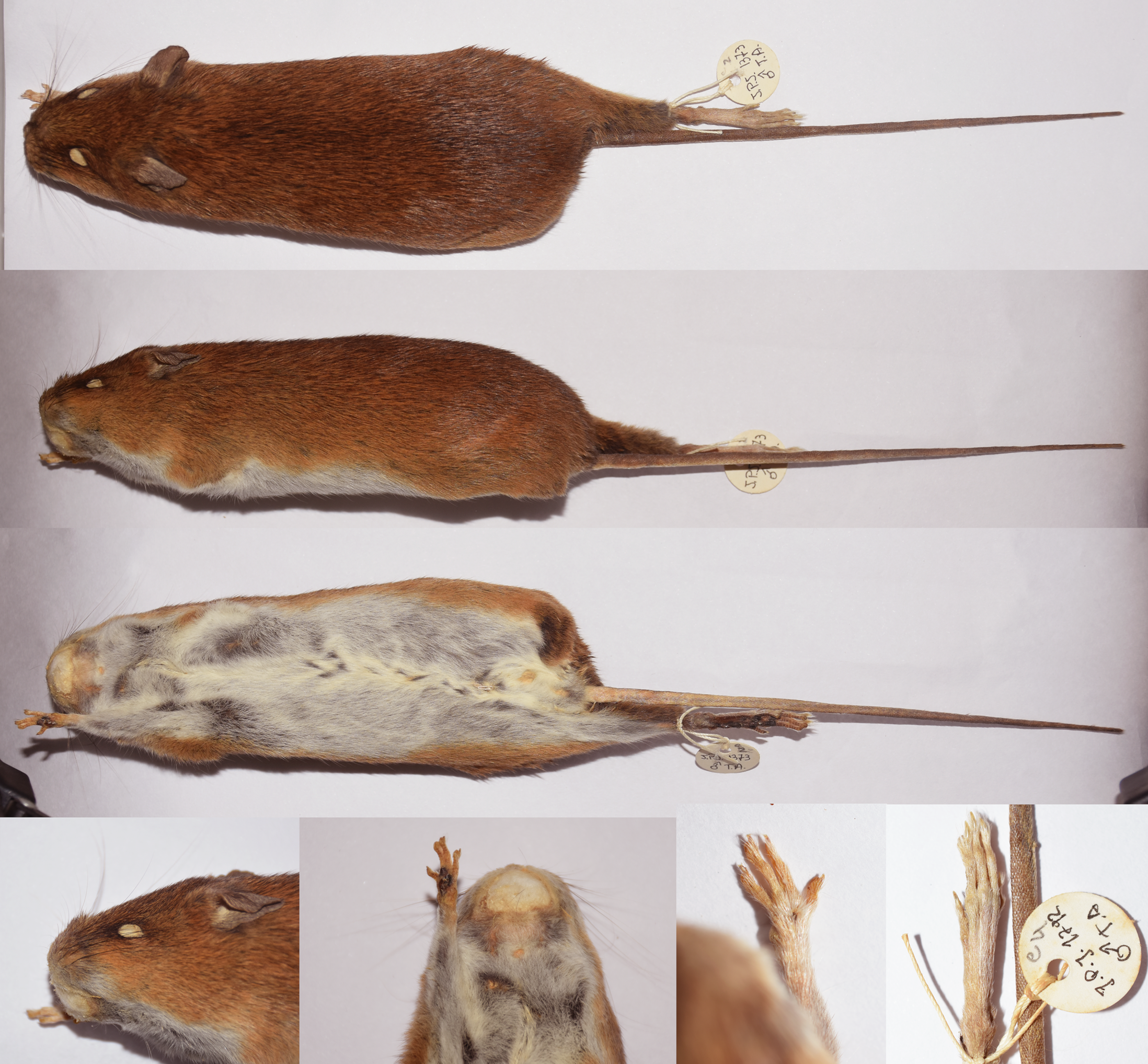

Supplement: Supplemental Information 2 — Images show dorsum, flanks, belly, tail, and fore and hind feet. Details of the blackish eyering and the small white spot on the chin. [file peerj-08-9884-s002.png]

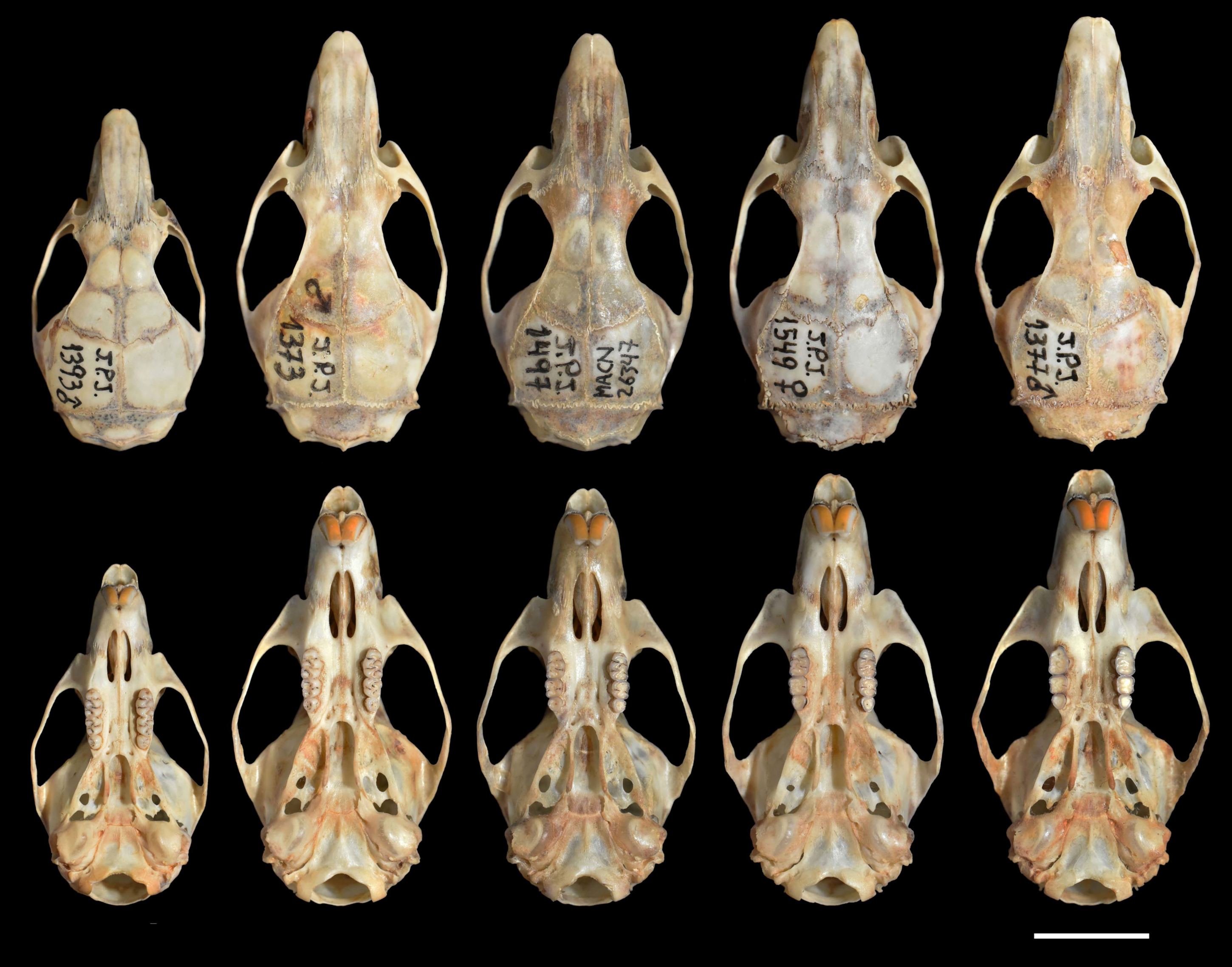

Supplement: Supplemental Information 3 — Age class 1 to age class 5 (from left to right). [file peerj-08-9884-s003.png]

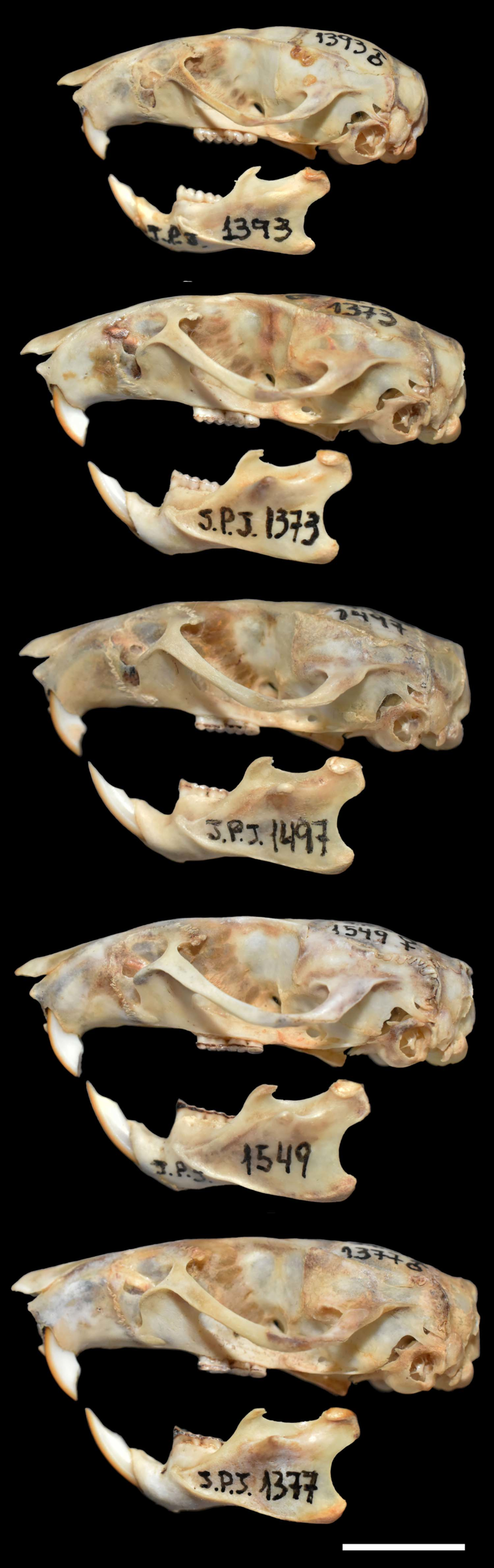

Supplement: Supplemental Information 4 — Age class 1 to age class 5 (from top to bottom). [file peerj-08-9884-s004.png]

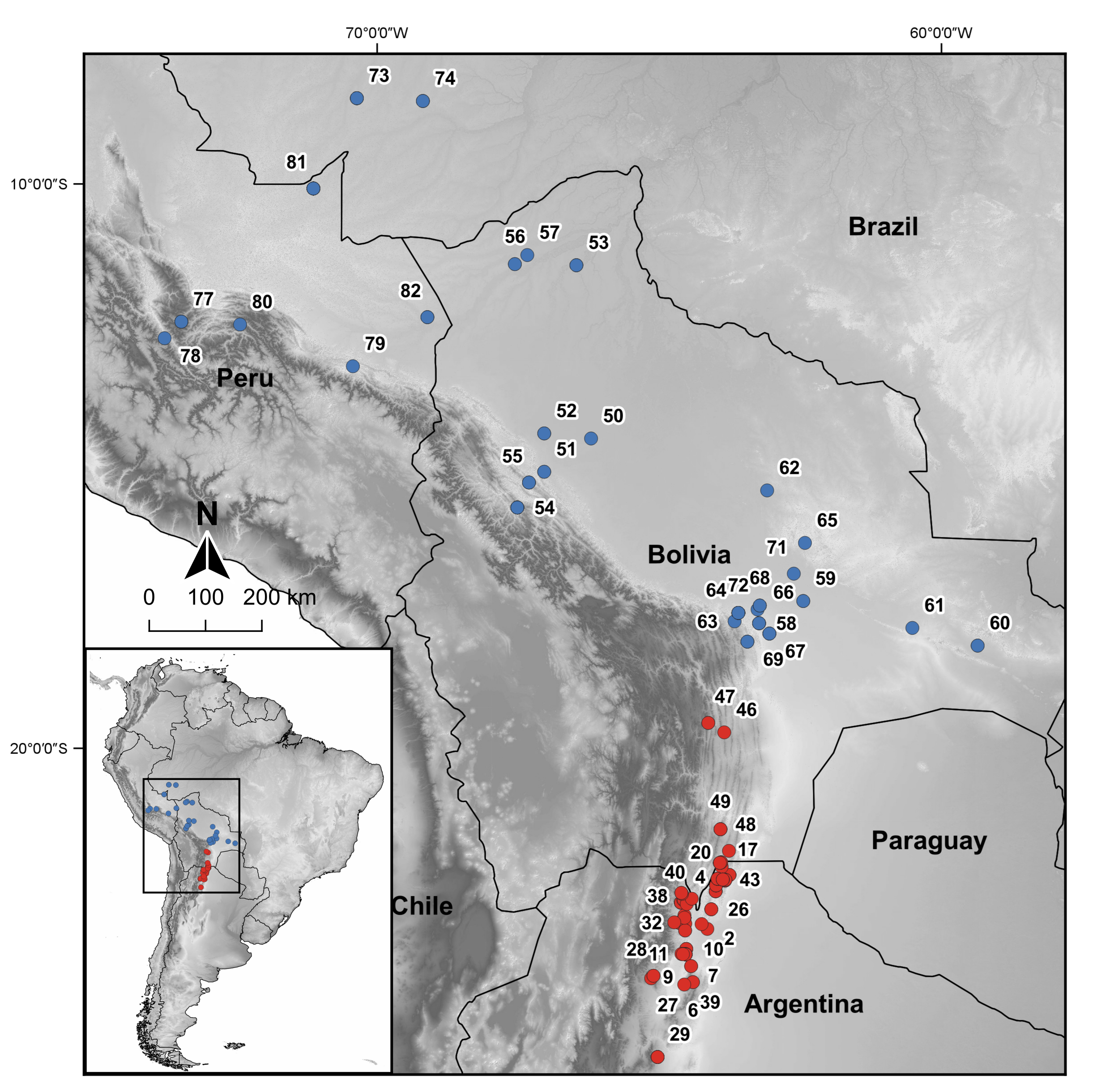

Supplement: Supplemental Information 5 — Localities numbered from 1 to 82 refer to the Euryoryzomys legatus and E. nitidus included in the morphological analyses. [file peerj-08-9884-s005.png]

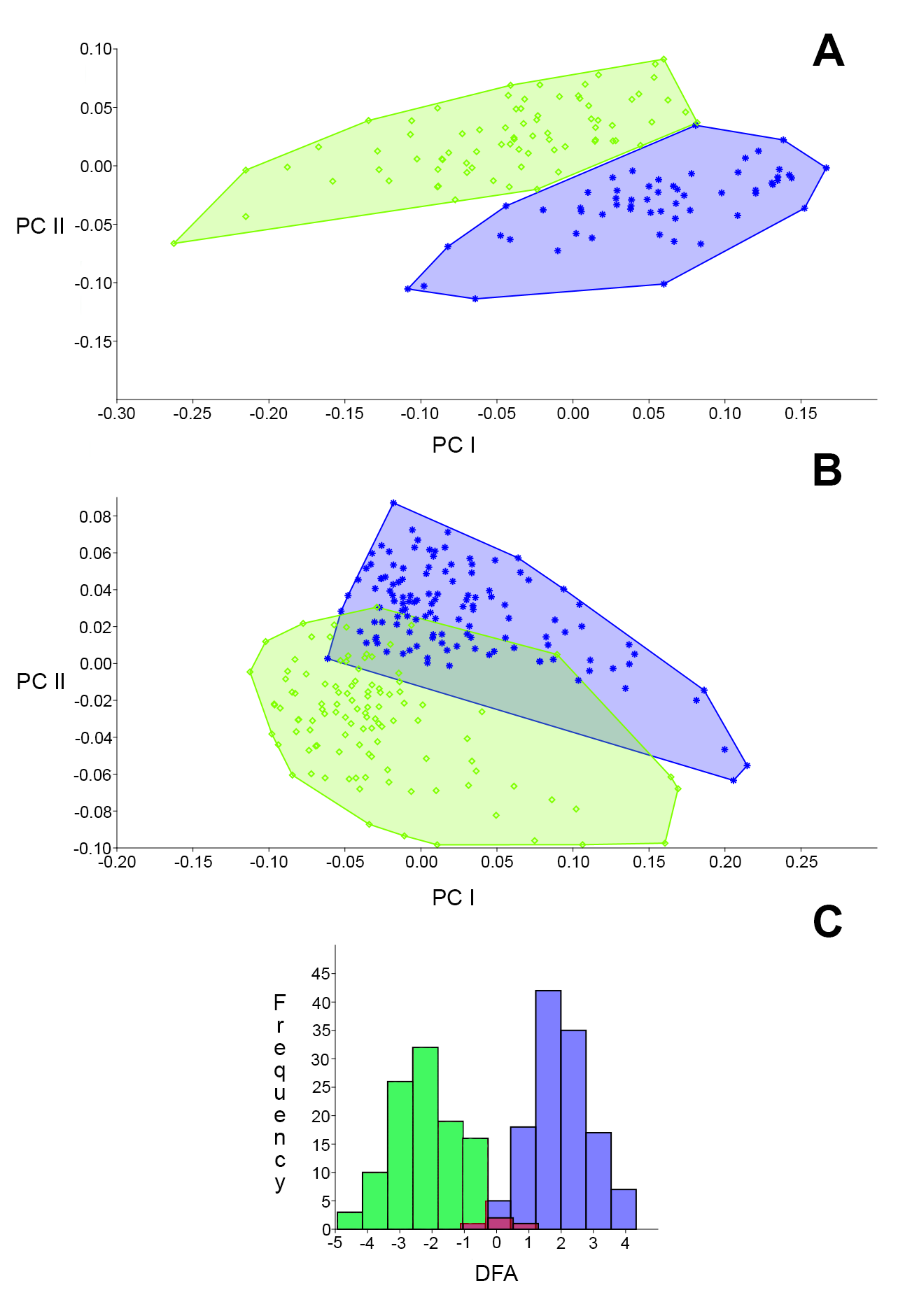

Supplement: Supplemental Information 6 — (A) Individual specimen scores based on log-transformed values of 16 cranial measurements projected onto the first and second principal components of the “size” PCA. Character loadings and the variance explained by each of the first 3 principal components are in Table 1. (B) Individual specimen scores based on log-transformed values of 16 cranial measurements (Mosimann shape variables) projected onto the first and second principal components of the “size-free” PCA. Character loadings and the variance explained by each of the first 3 principal components appear in Table 2. (C) Frequency distribution of the specimen onto the first discriminant function of the “size-free” DFA. Character loadings and the variance explained by the DFA are in Table 3. [file peerj-08-9884-s006.png]
