# Supplementary material for: A review of Euryoryzomys legatus (Rodentia, Sigmodontinae): morphological redescription, cytogenetics, and molecular phylogeny
Source: PeerJ. 2020 Oct 29;8:e9884. doi: 10.7717/peerj.9884 (PMC7603791; doi:10.7717/peerj.9884)
Supplement: Supplemental Information 7 — Taxon, voucher and field number are as indicated in Figures 5 and 6. E. legatus’ specimens used in the morphological analyses are indicated. GenBank accession numbers are also indicated. Samples in bold represent DNA sequences generated by this work. [file peerj-08-9884-s007.docx]

| **Species** | **Voucher** | **Field Number** | **Locality** | **City** | **Department, State or Province** | **Country** | **Latitud** | **Longitud** | **Map#** | **Morphology** | **Cytb** | **GenBank Accession** | **COXI** | **GenBank Accession** | **Reference** |
| --- | --- | --- | --- | --- | --- | --- | --- | --- | --- | --- | --- | --- | --- | --- | --- |
| *E. legatus* | MACN-Ma30.286 |  | Aguaray, 700 M | Aguaray |  | Argentina | -22.2447 | -63.7569 | 1 | X |  |  |  |  |  |
| *E. legatus* | MACN-Ma30.287 |  | Aguaray, 700 M | Aguaray |  | Argentina | -22.2447 | -63.7569 | 1 | X |  |  |  |  |  |
| *E. legatus* | MACN-Ma30.324 |  | Aguaray, 700 M | Aguaray |  | Argentina | -22.2447 | -63.7569 | 1 | X |  |  |  |  |  |
| *E. legatus* | MACN-Ma30.325 |  | Aguaray, 700 M | Aguaray |  | Argentina | -22.2447 | -63.7569 | 1 | X |  |  |  |  |  |
| *E. legatus* | CJC2182 |  | Fronteira Oran | Oran |  | Argentina | -23.2 | -64.15 | 2 | X |  |  |  |  |  |
| *E. legatus* | CEM3986 |  | P.N. Baritú 27 Km No Agua Blanca | Oran |  | Argentina | -22.733 | -64.6175 | 3 | X |  |  |  |  |  |
| *E. legatus* | CEM4110 |  | P.N. Baritú 27 Km No Agua Blanca | Oran |  | Argentina | -22.733 | -64.6175 | 3 | X |  |  |  |  |  |
| *E. legatus* | CEM4259 |  | P.N. Baritú 27 Km No Agua Blanca | Oran |  | Argentina | -22.733 | -64.6175 | 3 | X |  |  |  |  |  |
| *E. legatus* | CEM4290 |  | P.N. Baritú 27 Km No Agua Blanca | Oran |  | Argentina | -22.733 | -64.6175 | 3 | X |  |  |  |  |  |
| *E. legatus* | CEM4304 |  | P.N. Baritú 27 Km No Agua Blanca | Oran |  | Argentina | -22.733 | -64.6175 | 3 | X |  |  |  |  |  |
| *E. legatus* | CEM5864 |  | P.N. Baritú 27 Km No Agua Blanca | Oran |  | Argentina | -22.733 | -64.6175 | 3 | X |  |  |  |  |  |
| *E. legatus* | MACN-Ma17853 |  | P.N. Baritú 27 Km No Agua Blanca | Oran |  | Argentina | -22.733 | -64.6175 | 3 | X |  |  |  |  |  |
| *E. legatus* | MACN-Ma17856 |  | P.N. Baritú 27 Km No Agua Blanca | Oran |  | Argentina | -22.733 | -64.6175 | 3 | X |  |  |  |  |  |
| *E. legatus* | MACN-Ma17857 |  | P.N. Baritú 27 Km No Agua Blanca | Oran |  | Argentina | -22.733 | -64.6175 | 3 | X |  |  |  |  |  |
| *E. legatus* | MACN-Ma17858 |  | P.N. Baritú 27 Km No Agua Blanca | Oran |  | Argentina | -22.733 | -64.6175 | 3 | X |  |  |  |  |  |
| *E. legatus* | MACN-Ma17859 |  | P.N. Baritú 27 Km No Agua Blanca | Oran |  | Argentina | -22.733 | -64.6175 | 3 | X |  |  |  |  |  |
| *E. legatus* | CML48 |  | Rio Pescado Angosto, Aguas Blancas | Oran |  | Argentina | -22.6847 | -64.5764 | 4 | X |  |  |  |  |  |
| *E. legatus* | CML290 |  | Rio Pescado, Finca Ypf | Oran |  | Argentina | -22.683 | -64.566 | 5 | X |  |  |  |  |  |
| *E. legatus* | CML2013 |  | El Simbolar, 25 Km So Palma Sola |  |  | Argentina | -24.1429 | -64.4046 | 6 | X |  |  |  |  |  |
| *E. legatus* | CML2014 |  | El Simbolar, 25 Km So Palma Sola | Santa Barbara |  | Argentina | -24.1429 | -64.4046 | 6 | X |  |  |  |  |  |
| *E. legatus* | CJC2202 |  | Laguna 'La Brea', 25 Km Antes De Palma Sola, Sobre Ruta 1 | Santa Barbara |  | Argentina | -23.8593 | -64.4366 | 7 | X |  |  |  |  |  |
| *E. legatus* | CML9690 |  | A 11 Km Intersección Ruta 34, Camino A Acambuco | Gral. San Martín |  | Argentina | -22.3443 | -63.8354 | 8 | X |  |  |  |  |  |
| *E. legatus* | CML9688 |  | 13 Km Al Nw De Yuto, Sobre Arroyo Yuto | Ledesma |  | Argentina | -23.6431 | -64.5414 | 9 | X |  |  |  |  |  |
| *E. legatus* | CML9689 |  | 13 Km Al Nw De Yuto, Sobre Arroyo Yuto | Ledesma |  | Argentina | -23.6431 | -64.5414 | 9 | X |  |  |  |  |  |
| *E. legatus* | CML8334 |  | 2.5 Km W El Bananal, 437 M | Ledesma |  | Argentina | -23.5547 | -64.5233 | 10 | X |  |  |  |  |  |
| *E. legatus* | CML8335 |  | 2.5 Km W El Bananal, 437 M | Ledesma |  | Argentina | -23.5547 | -64.5233 | 10 | X |  |  |  |  |  |
| *E. legatus* | CML9684 |  | 2.5 Km W El Bananal, 437 M | Ledesma |  | Argentina | -23.5547 | -64.5233 | 10 | X |  |  |  |  |  |
| *E. legatus* | CML9685 |  | 2.5 Km W El Bananal, 437 M | Ledesma |  | Argentina | -23.5547 | -64.5233 | 10 | X |  |  |  |  |  |
| ***E. legatus*** | **CML13250** | **JPJ2681** | **Arroyo Yuto, 13 Km Al So De Yuto** | **Ledesma** |  | **Argentina** | **-23.6428** | **-64.542** | **11** | **X** | **X** | **MT118039** | **X** | **MT118080** | **This study** |
| ***E. legatus*** | **CML13251** | **JPJ2682** | **Arroyo Yuto, 13 Km Al So De Yuto** | **Ledesma** |  | **Argentina** | **-23.6428** | **-64.542** | **11** | **X** | **X** | **MT118040** | **X** | **MT118081** | **This study** |
| *E. legatus* | CML13252 | JPJ1958 | Finca Famat, Aprox. 3 Km Al Sw Del Portón De Entrada De La Finca, 400 M | Ledesma |  | Argentina | -23.6482 | -64.5316 | 12 | X |  |  |  |  |  |
| *E. legatus* | CML13253 | JPJ1562 | Finca Sauzalito, Aprox. 1 Km Al E Del Cruce Entre La Ruta Nacional 34 Y El Río Yuto, 413 M | Ledesma |  | Argentina | -23.6513 | -64.5341 | 13 | X |  |  |  |  |  |
| *E. legatus* | CML13254 | JPJ1564 | Finca Sauzalito, Aprox. 1 Km Al E Del Cruce Entre La Ruta Nacional 34 Y El Río Yuto, 413 M | Ledesma |  | Argentina | -23.6513 | -64.5341 | 13 | X |  |  |  |  |  |
| *E. legatus* | CML13255 | JPJ1565 | Finca Sauzalito, Aprox. 1 Km Al E Del Cruce Entre La Ruta Nacional 34 Y El Río Yuto, 413 M | Ledesma |  | Argentina | -23.6513 | -64.5341 | 13 | X |  |  |  |  |  |
| *E. legatus* | MACN-Ma26338 | JPJ1566 | Finca Sauzalito, Aprox. 1 Km Al E Del Cruce Entre La Ruta Nacional 34 Y El Río Yuto, 413 M | Ledesma |  | Argentina | -23.6513 | -64.5341 | 13 | X |  |  |  |  |  |
| *E. legatus* | MACN-Ma26352 | JPJ1570 | Finca Sauzalito, Aprox. 1 Km Al E Del Cruce Entre La Ruta Nacional 34 Y El Río Yuto, 413 M | Ledesma |  | Argentina | -23.6513 | -64.5341 | 13 | X |  |  |  |  |  |
| *E. legatus* | MACN-Ma26353 | JPJ1558 | Finca Sauzalito, Aprox. 1 Km Al E Del Cruce Entre La Ruta Nacional 34 Y El Río Yuto, 413 M | Ledesma |  | Argentina | -23.6513 | -64.5341 | 13 | X |  |  |  |  |  |
| *E. legatus* | MACN-Ma26354 | JPJ1560 | Finca Sauzalito, Aprox. 1 Km Al E Del Cruce Entre La Ruta Nacional 34 Y El Río Yuto, 413 M | Ledesma |  | Argentina | -23.6513 | -64.5341 | 13 | X |  |  |  |  |  |
| *E. legatus* | MACN-Ma26355 | JPJ1568 | Finca Sauzalito, Aprox. 1 Km Al E Del Cruce Entre La Ruta Nacional 34 Y El Río Yuto, 413 M | Ledesma |  | Argentina | -23.6513 | -64.5341 | 13 | X |  |  |  |  |  |
| *E. legatus* | MACN-Ma27068 | JPJ1563 | Finca Sauzalito, Aprox. 1 Km Al E Del Cruce Entre La Ruta Nacional 34 Y El Río Yuto, 413 M | Ledesma |  | Argentina | -23.6513 | -64.5341 | 13 | X |  |  |  |  |  |
| ***E. legatus*** | **MACN-Ma27069** | **JPJ2050** | **Planta Caimancito, Batería, 535 M** | **Ledesma** |  | **Argentina** | **-23.6447** | **-64.6033** | **14** | **X** | **X** | **MT118042** |  |  | **This study** |
| *E. legatus* | CML13256 | JPJ1974 | Planta Caimancito, Camino Al Pozo 40, 508 M | Ledesma |  | Argentina | -23.6431 | -64.598 | 15 | X |  |  |  |  |  |
| *E. legatus* | CML13257 | JPJ1975 | Planta Caimancito, Camino Al Pozo 40, 508 M | Ledesma |  | Argentina | -23.6431 | -64.598 | 15 | X |  |  |  |  |  |
| *E. legatus* | CML13258 | JPJ2053 | Planta Caimancito, Camino Al Pozo 40, 508 M | Ledesma |  | Argentina | -23.6431 | -64.598 | 15 | X |  |  |  |  |  |
| *E. legatus* | MACN-Ma26357 | JPJ2051 | Planta Caimancito, Pozo 43, 560 M | Ledesma |  | Argentina | -23.6419 | -64.6074 | 16 | X |  |  |  |  |  |
| *E. legatus* | CML13262 | JPJ1353 | Bajo E. Macconnelliueta, Aprox. 10 Km Al Ne De Acambuco, Sobre Quebrada San Francisco, 736 M | Lib. Gral. San Martín |  | Argentina | -22.0993 | -63.8943 | 17 | X |  |  |  |  |  |
| *E. legatus* | CML13264 | JPJ1347 | Campo Largo, Km 74.5 De La Ruta Entre Acambuco Y Campo Largo, 684 M | Lib. Gral. San Martín |  | Argentina | -22.0287 | -63.9266 | 18 | X |  |  |  |  |  |
| *E. legatus* | CML13265 | JPJ1351 | Campo Largo, Km 74.5 De La Ruta Entre Acambuco Y Campo Largo, 684 M | Lib. Gral. San Martín |  | Argentina | -22.0287 | -63.9266 | 18 | X |  |  |  |  |  |
| *E. legatus* | CRILAR-Ma275 | JPJ1350 | Campo Largo, Km 74.5 De La Ruta Entre Acambuco Y Campo Largo, 684 M | Lib. Gral. San Martín |  | Argentina | -22.0287 | -63.9266 | 18 | X |  |  |  |  |  |
| *E. legatus* | CRILAR-Ma279 | JPJ1348 | Campo Largo, Km 74.5 De La Ruta Entre Acambuco Y Campo Largo, 684 M | Lib. Gral. San Martín |  | Argentina | -22.0287 | -63.9266 | 18 | X |  |  |  |  |  |
| *E. legatus* | MACN-Ma26344 | JPJ1349 | Campo Largo, Km 74.5 De La Ruta Entre Acambuco Y Campo Largo, 684 M | Lib. Gral. San Martín |  | Argentina | -22.0287 | -63.9266 | 18 | X |  |  |  |  |  |
| *E. legatus* | MACN-Ma29258 | JPJ1346 | Campo Largo, Km 74.5 De La Ruta Entre Acambuco Y Campo Largo, 684 M | Lib. Gral. San Martín |  | Argentina | -22.0287 | -63.9266 | 18 | X |  |  |  |  |  |
| *E. legatus* | CML13274 | JPJ1340 | E. Macueta Norte, Km 72 De La Ruta Al Pozo 1001 B, 1148 M | Lib. Gral. San Martín |  | Argentina | -22.0205 | -63.9064 | 19 | X |  |  |  |  |  |
| *E. legatus* | CML13275 | JPJ1341 | E. Macueta Norte, Km 72 De La Ruta Al Pozo 1001 B, 1148 M | Lib. Gral. San Martín |  | Argentina | -22.0205 | -63.9064 | 19 | X |  |  |  |  |  |
| *E. legatus* | CML13276 | JPJ1345 | E. Macueta Norte, Km 72 De La Ruta Al Pozo 1001 B, 1148 M | Lib. Gral. San Martín |  | Argentina | -22.0205 | -63.9064 | 19 | X |  |  |  |  |  |
| *E. legatus* | MACN-Ma26340 | JPJ1342 | E. Macueta Norte, Km 72 De La Ruta Al Pozo 1001 B, 1148 M | Lib. Gral. San Martín |  | Argentina | -22.0205 | -63.9064 | 19 | X |  |  |  |  |  |
| *E. legatus* | MACN-Ma26343 | JPJ1343 | E. Macueta Norte, Km 72 De La Ruta Al Pozo 1001 B, 1148 M | Lib. Gral. San Martín |  | Argentina | -22.0205 | -63.9064 | 19 | X |  |  |  |  |  |
| *E. legatus* | CML13266 | JPJ1354 | Finca Falcón, Aprox. 3 Km Al Nw Del Pozo De Agua N° 5, Cercanías Del Río Seco, 704 M. | Lib. Gral. San Martín |  | Argentina | -22.3115 | -63.9686 | 20 | X |  |  |  |  |  |
| *E. legatus* | CML13267 | JPJ1355 | Finca Falcón, Aprox. 3 Km Al Nw Del Pozo De Agua N° 5, Cercanías Del Río Seco, 704 M. | Lib. Gral. San Martín |  | Argentina | -22.3115 | -63.9686 | 20 | X |  |  |  |  |  |
| *E. legatus* | CML13268 | JPJ1357 | Finca Falcón, Aprox. 3 Km Al Nw Del Pozo De Agua N° 5, Cercanías Del Río Seco, 704 M. | Lib. Gral. San Martín |  | Argentina | -22.3115 | -63.9686 | 20 | X |  |  |  |  |  |
| *E. legatus* | CRILAR-Ma278 | JPJ1487 | Aprox. 21 Km (Por Ruta) Al Wnw De Vespucio, Sobre El Río Seco, 532 M. | Libertador General San Martín |  | Argentina | -22.5324 | -64.0032 | 21 | X |  |  |  |  |  |
| *E. legatus* | MACN-Ma26345 | JPJ1484 | Aprox. 21 Km (Por Ruta) Al Wnw De Vespucio, Sobre El Río Seco, 532 M. | Libertador General San Martín |  | Argentina | -22.5324 | -64.0032 | 21 | X |  |  |  |  |  |
| *E. legatus* | MACN-Ma26346 | JPJ1496 | Aprox. 21 Km (Por Ruta) Al Wnw De Vespucio, Sobre El Río Seco, 532 M. | Libertador General San Martín |  | Argentina | -22.5324 | -64.0032 | 21 | X |  |  |  |  |  |
| *E. legatus* | MACN-Ma26347 | JPJ1498 | Aprox. 21 Km (Por Ruta) Al Wnw De Vespucio, Sobre El Río Seco, 532 M. | Libertador General San Martín |  | Argentina | -22.5324 | -64.0032 | 21 | X |  |  |  |  |  |
| *E. legatus* | MACN-Ma26348 |  | Aprox. 21 Km (Por Ruta) Al Wnw De Vespucio, Sobre El Río Seco, 532 M. | Libertador General San Martín |  | Argentina | -22.5324 | -64.0032 | 21 | X |  |  |  |  |  |
| *E. legatus* | CML13260 |  | Aprox. 35 Km (Por Ruta) Al Nw De Vespucio, 635 M | Libertador General San Martín |  | Argentina | -22.4281 | -63.9951 | 22 | X |  |  |  |  |  |
| *E. legatus* | CML13261 |  | Aprox. 35 Km (Por Ruta) Al Nw De Vespucio, 635 M | Libertador General San Martín |  | Argentina | -22.4281 | -63.9951 | 22 | X |  |  |  |  |  |
| *E. legatus* | MACN-Ma27066 |  | Aprox. 35 Km (Por Ruta) Al Nw De Vespucio, 635 M | Libertador General San Martín |  | Argentina | -22.4281 | -63.9951 | 22 | X |  |  |  |  |  |
| *E. legatus* | MACN-Ma27067 |  | Aprox. 35 Km (Por Ruta) Al Nw De Vespucio, 635 M | Libertador General San Martín |  | Argentina | -22.4281 | -63.9951 | 22 | X |  |  |  |  |  |
| *E. legatus* | MACN-Ma29260 | JPJ1557 | Aprox. 35 Km (Por Ruta) Al Nw De Vespucio, 635 M | Libertador General San Martín |  | Argentina | -22.4281 | -63.9951 | 22 | X |  |  |  |  |  |
| *E. legatus* | CML13263 | JPJ1514 | Camino A La Antena Repetidora Yacuy, Altura Del Km 12.5 De La Ruta Provincial Nº 54, Aprox. 13 Km (Por Ruta) Al W De Piquirenda, 837 M | Libertador General San Martín |  | Argentina | -22.3301 | -63.8302 | 23 | X |  |  |  |  |  |
| *E. legatus* | MACN-Ma26341 | JPJ1513 | Camino A La Antena Repetidora Yacuy, Altura Del Km 12.5 De La Ruta Provincial Nº 54, Aprox. 13 Km (Por Ruta) Al W De Piquirenda, 837 M | Libertador General San Martín |  | Argentina | -22.3301 | -63.8302 | 23 | X |  |  |  |  |  |
| *E. legatus* | MACN-Ma26342 | JPJ1511 | Camino A La Antena Repetidora Yacuy, Altura Del Km 12.5 De La Ruta Provincial Nº 54, Aprox. 13 Km (Por Ruta) Al W De Piquirenda, 837 M | Libertador General San Martín |  | Argentina | -22.3301 | -63.8302 | 23 | X |  |  |  |  |  |
| *E. legatus* | MACN-Ma27070 | JPJ1512 | Camino A La Antena Repetidora Yacuy, Altura Del Km 12.5 De La Ruta Provincial Nº 54, Aprox. 13 Km (Por Ruta) Al W De Piquirenda, 837 M | Libertador General San Martín |  | Argentina | -22.3301 | -63.8302 | 23 | X |  |  |  |  |  |
| ***E. legatus*** | **CRILAR-Ma277** | **JPJ1542** | **Campo Largo, Km 74.5 De La Ruta Entre Acambuco Y Campo Largo, 684 M** | **Libertador General San Martín** |  | **Argentina** | **-22.0287** | **-63.9266** | **24** | **X** | **X** | **MT118038** | **X** | **MT118079** | **This study** |
| *E. legatus* | MACN-Ma29259 | JPJ1541 | Campo Largo, Km 74.5 De La Ruta Entre Acambuco Y Campo Largo, 684 M | Libertador General San Martín |  | Argentina | -22.0287 | -63.9266 | 24 | X |  |  |  |  |  |
| *E. legatus* | CML13269 | JPJ1377 | Finca Falcón, Aprox. 3 Km Al Nw Del Pozo De Agua N° 5, Cercanías Del Río Seco, 704 M. | Libertador General San Martín |  | Argentina | -22.3115 | -63.9686 | 25 | X |  |  |  |  |  |
| *E. legatus* | CML13270 | JPJ1380 | Finca Falcón, Aprox. 3 Km Al Nw Del Pozo De Agua N° 5, Cercanías Del Río Seco, 704 M. | Libertador General San Martín |  | Argentina | -22.3115 | -63.9686 | 25 | X |  |  |  |  |  |
| *E. legatus* | CML13271 | JPJ1381 | Finca Falcón, Aprox. 3 Km Al Nw Del Pozo De Agua N° 5, Cercanías Del Río Seco, 704 M. | Libertador General San Martín |  | Argentina | -22.3115 | -63.9686 | 25 | X |  |  |  |  |  |
| *E. legatus* | CML13272 | JPJ1391 | Finca Falcón, Aprox. 3 Km Al Nw Del Pozo De Agua N° 5, Cercanías Del Río Seco, 704 M. | Libertador General San Martín |  | Argentina | -22.3115 | -63.9686 | 25 | X |  |  |  |  |  |
| *E. legatus* | CRILAR-Ma276 | JPJ1373 | Finca Falcón, Aprox. 3 Km Al Nw Del Pozo De Agua N° 5, Cercanías Del Río Seco, 704 M. | Libertador General San Martín |  | Argentina | -22.3115 | -63.9686 | 25 | X |  |  |  |  |  |
| *E. legatus* | MACN-Ma26337 | JPJ1393 | Finca Falcón, Aprox. 3 Km Al Nw Del Pozo De Agua N° 5, Cercanías Del Río Seco, 704 M. | Libertador General San Martín |  | Argentina | -22.3115 | -63.9686 | 25 | X |  |  |  |  |  |
| *E. legatus* | MACN-Ma26349 | JPJ1363 | Finca Falcón, Aprox. 3 Km Al Nw Del Pozo De Agua N° 5, Cercanías Del Río Seco, 704 M. | Libertador General San Martín |  | Argentina | -22.3115 | -63.9686 | 25 | X |  |  |  |  |  |
| *E. legatus* | MACN-Ma26350 | JPJ1382 | Finca Falcón, Aprox. 3 Km Al Nw Del Pozo De Agua N° 5, Cercanías Del Río Seco, 704 M. | Libertador General San Martín |  | Argentina | -22.3115 | -63.9686 | 25 | X |  |  |  |  |  |
| *E. legatus* | MACN-Ma26351 | JPJ1390 | Finca Falcón, Aprox. 3 Km Al Nw Del Pozo De Agua N° 5, Cercanías Del Río Seco, 704 M. | Libertador General San Martín |  | Argentina | -22.3115 | -63.9686 | 25 | X |  |  |  |  |  |
| *E. legatus* | CML13273 | JPJ1832 | Finca Río Seco, 40 Km Al N De Embarcación, 524 M | Libertador General San Martín |  | Argentina | -22.8518 | -64.0793 | 26 | X |  |  |  |  |  |
| ***E. legatus*** | **MACN-Ma26339** | **JPJ1831** | **Finca Río Seco, 40 Km Al N De Embarcación, 524 M** | **Libertador General San Martín** |  | **Argentina** | **-22.8518** | **-64.0793** | **26** | **X** | **X** | **MT118041** | **X** | **MT118082** | **This study** |
| *E. legatus* | MACN-Ma26356 | JPJ1835 | Finca Río Seco, 40 Km Al N De Embarcación, 524 M | Libertador General San Martín |  | Argentina | -22.8518 | -64.0793 | 26 | X |  |  |  |  |  |
| *E. legatus* | CML12383 |  | Arroyo Los Matos, 7 Km Al N De Las Capillas | Manuel Belgrano |  | Argentina | -24.0744 | -65.145 | 27 | X |  |  |  |  |  |
| *E. legatus* | CML8330 |  | Río Las Capillas, 15 Km Al N De Las Capillas, Por Ruta 20, 927 M | Manuel Belgrano |  | Argentina | -24.0296 | -65.1038 | 28 | X |  |  |  |  |  |
| *E. legatus* | CML4327 |  | Río Las Capillas, 15 Km Al N De Las Capillas, Por Ruta 20, 957 M | Manuel Belgrano |  | Argentina | -24.0296 | -65.1038 | 28 | X |  |  |  |  |  |
| *E. legatus* | CML8327 |  | Río De Las Conchas, 2 Km Al N Y 6 Km Al O De Metán, 1027 M | Metán |  | Argentina | -25.4694 | -65.0308 | 29 | X |  |  |  |  |  |
| *E. legatus* | CML8329 |  | Río De Las Conchas, 2 Km Al N Y 6 Km Al O De Metán, 1027 M | Metán |  | Argentina | -25.4694 | -65.0308 | 29 | X |  |  |  |  |  |
| *E. legatus* | CML9714 |  | Río De Las Conchas, 2 Km Al N Y 6 Km Al O De Metán, 1027 M | Metán |  | Argentina | -25.4694 | -65.0308 | 29 | X |  |  |  |  |  |
| *E. legatus* | CML9715 |  | Río De Las Conchas, 2 Km Al N Y 6 Km Al O De Metán, 1027 M | Metán |  | Argentina | -25.4694 | -65.0308 | 29 | X |  |  |  |  |  |
| *E. legatus* | CML9717 |  | Río De Las Conchas, 2 Km Al N Y 6 Km Al O De Metán, 1027 M | Metán |  | Argentina | -25.4694 | -65.0308 | 29 | X |  |  |  |  |  |
| *E. legatus* | CML9349 |  | Río De Las Conchas, 4.7 Km Al W De Metán | Metán |  | Argentina | -25.4694 | -65.0308 | 29 | X |  |  |  |  |  |
| *E. legatus* | CML9350 |  | Río De Las Conchas, 4.7 Km Al W De Metán | Metán |  | Argentina | -25.4694 | -65.0308 | 29 | X |  |  |  |  |  |
| *E. legatus* | CML9351 |  | Río De Las Conchas, 4.7 Km Al W De Metán | Metán |  | Argentina | -25.4694 | -65.0308 | 29 | X |  |  |  |  |  |
| *E. legatus* | CML9352 |  | Río De Las Conchas, 4.7 Km Al W De Metán | Metán |  | Argentina | -25.4694 | -65.0308 | 29 | X |  |  |  |  |  |
| *E. legatus* | CML9708 |  | Río De Las Conchas, 5.7 Km W Metán, Sobre El Río, 996 M | Metán |  | Argentina | -25.4694 | -65.0308 | 29 | X |  |  |  |  |  |
| *E. legatus* | CML9709 |  | Río De Las Conchas, 5.7 Km W Metán, Sobre El Río, 996 M | Metán |  | Argentina | -25.4694 | -65.0308 | 29 | X |  |  |  |  |  |
| *E. legatus* | CML9710 |  | Río De Las Conchas, 5.7 Km W Metán, Sobre El Río, 996 M | Metán |  | Argentina | -25.4694 | -65.0308 | 29 | X |  |  |  |  |  |
| *E. legatus* | CML9711 |  | Río De Las Conchas, 5.7 Km W Metán, Sobre El Río, 996 M | Metán |  | Argentina | -25.4694 | -65.0308 | 29 | X |  |  |  |  |  |
| *E. legatus* | CML9712 |  | Río De Las Conchas, 5.7 Km W Metán, Sobre El Río, 996 M | Metán |  | Argentina | -25.4694 | -65.0308 | 29 | X |  |  |  |  |  |
| *E. legatus* | CML8328 |  | 43,7 Km Al Nw De Cruce De Ruta 50 Y 18, Camino A Isla De Canas | Orán |  | Argentina | -23.0027 | -64.5527 | 30 | X |  |  |  |  |  |
| *E. legatus* | CML9698 |  | 43,7 Km Al Nw De Cruce De Ruta 50 Y 18, Camino A Isla De Canas | Orán |  | Argentina | -23.0027 | -64.5527 | 30 | X |  |  |  |  |  |
| *E. legatus* | CML9700 |  | 43,7 Km Al Nw De Cruce De Ruta 50 Y 18, Camino A Isla De Canas | Orán |  | Argentina | -23.0027 | -64.5527 | 30 | X |  |  |  |  |  |
| *E. legatus* | CML9701 |  | 43,7 Km Al Nw De Cruce De Ruta 50 Y 18, Camino A Isla De Canas | Orán |  | Argentina | -23.0027 | -64.5527 | 30 | X |  |  |  |  |  |
| *E. legatus* | CML9703 |  | 43,7 Km Al Nw De Cruce De Ruta 50 Y 18, Camino A Isla De Canas | Orán |  | Argentina | -23.0027 | -64.5527 | 30 | X |  |  |  |  |  |
| *E. legatus* | CML9704 |  | 43,7 Km Al Nw De Cruce De Ruta 50 Y 18, Camino A Isla De Canas | Orán |  | Argentina | -23.0027 | -64.5527 | 30 | X |  |  |  |  |  |
| *E. legatus* | CML9699 |  | 43,7 Km Al Nw De Cruce De Ruta 50 Y 18, Camino A Isla De Canas, Sobre Ruta 18 | Orán |  | Argentina | -23.0027 | -64.5527 | 30 | X |  |  |  |  |  |
| *E. legatus* | CML9705 |  | 43,7 Km Al Nw De Cruce De Ruta 50 Y 18, Camino A Isla De Canas, Sobre Ruta 18 | Orán |  | Argentina | -23.0027 | -64.5527 | 30 | X |  |  |  |  |  |
| *E. legatus* | CML9706 |  | 43,7 Km Al Nw De Cruce De Ruta 50 Y 18, Camino A Isla De Canas, Sobre Ruta 18 | Orán |  | Argentina | -23.0027 | -64.5527 | 30 | X |  |  |  |  |  |
| *E. legatus* | CML9707 |  | 48,8 Km Nw Cruce Rutas 50 Y 18, Camino A Isla De Cañas, Sobre Ruta 18 | Orán |  | Argentina | -22.9583 | -64.555 | 31 | X |  |  |  |  |  |
| *E. legatus* | CML9695 |  | A 40 Km De Ruta 50 Por Ruta 18, Camino De Inspección Del Gasoducto Paralelo Al Camino De San Andrés, Ent. A La Válvula 3/4/4 Bis | Orán |  | Argentina | -23.0897 | -64.6533 | 32 | X |  |  |  |  |  |
| *E. legatus* | CML9696 |  | A 40 Km De Ruta 50 Por Ruta 18, Camino De Inspección Del Gasoducto Paralelo Al Camino De San Andrés, Ent. A La Válvula 3/4/4 Bis | Orán |  | Argentina | -23.0897 | -64.6533 | 32 | X |  |  |  |  |  |
| *E. legatus* | CML9697 |  | A 40 Km De Ruta 50 Por Ruta 18, Camino De Inspección Del Gasoducto Paralelo Al Camino De San Andrés, Ent. A La Válvula 3/4/4 Bis | Orán |  | Argentina | -23.0897 | -64.6533 | 32 | X |  |  |  |  |  |
| *E. legatus* | CML11598 |  | El Oculto, Ruta Prov. N° 18, Km 25 Sobre Río Blanco | Orán |  | Argentina | -23.1061 | -64.5403 | 33 | X |  |  |  |  |  |
| *E. legatus* | CML13277 | JPJ1792 | Finca Alto Verde, 670 M | Orán |  | Argentina | -23.2273 | -64.5431 | 34 | X |  |  |  |  |  |
| *E. legatus* | CRILAR-Ma280 | JPJ1801 | Finca Alto Verde, 670 M | Orán |  | Argentina | -23.2273 | -64.5431 | 34 | X |  |  |  |  |  |
| *E. legatus* | MACN-Ma28143 | JPJ1182 | Finca El Carmen (De D. Lutaif), 396 M | Orán |  | Argentina | -23.1142 | -64.2509 | 35 | X |  |  |  |  |  |
| *E. legatus* | CML8326 |  | Isla De Cañas, 25 Km Al S Por Ruta 18 | Orán |  | Argentina | -23.0017 | -64.5551 | 36 | X |  |  |  |  |  |
| *E. legatus* | CML11602 |  | Quebrada De Vides, Puesto Viejo, 4 Km Se De Angosto Del Pescado, Parque Provincial Laguna De Pintascayo | Orán |  | Argentina | -22.7575 | -64.5147 | 37 | X |  |  |  |  |  |
| *E. legatus* | CML11603 |  | Río Colorado, 25 Km O Del Oculto (Camino A San Andrés), 1008 M | Orán |  | Argentina | -23.0833 | -64.7364 | 38 | X |  |  |  |  |  |
| ***E. legatus*** | **CML13259** | **JPJ1109** | **Finca El Piquete, Orillas Del Río Volcán, 973 M** | **Santa Bárbara** |  | **Argentina** | **-24.1856** | **-64.5595** | **39** | **X** | **X** | **MT118037** | **X** | **MT118078** | **This study** |
| *E. legatus* | CML9691 |  | Arroyo Arrazayal, 18 Km Al Nw Del Cruce De Rutas 50 Y 19, Por Ruta 19, 450 M | Santa Victoria |  | Argentina | -22.6642 | -64.4253 | 40 | X |  |  |  |  |  |
| *E. legatus* | CML5525 |  | P.N. Baritú, Naciente Arroyo Santa Rosa, 900 M | Santa Victoria |  | Argentina | -22.5779 | -64.5988 | 41 | X |  |  |  |  |  |
| *E. legatus* | CML5518 |  | P.N. Baritú, Naciente Arroyo Santelmita, 900 M | Santa Victoria |  | Argentina | -22.5613 | -64.6104 | 42 | X |  |  |  |  |  |
| *E. legatus* | CML5528 |  | P.N. Baritú, Naciente Arroyo Santelmita, 900 M | Santa Victoria |  | Argentina | -22.5613 | -64.6104 | 42 | X |  |  |  |  |  |
| *E. legatus* | CML8336 |  | 12.6 Km W De Piquirenda Viejo, 750 M |  |  | Argentina | -22.3247 | -63.8761 | 43 | X |  |  |  |  |  |
| *E. legatus* | CML9692 |  | 12.6 Km W De Piquirenda Viejo, 750 M |  |  | Argentina | -22.3247 | -63.8761 | 43 | X |  |  |  |  |  |
| *E. legatus* | CML9693 |  | 12.6 Km W De Piquirenda Viejo, 750 M |  |  | Argentina | -22.3247 | -63.8761 | 43 | X |  |  |  |  |  |
| *E. legatus* | CML9694 |  | 12.6 Km W De Piquirenda Viejo, 750 M |  |  | Argentina | -22.3247 | -63.8761 | 43 | X |  |  |  |  |  |
| *E. legatus* | CML8507 |  | Laguna La Brea |  |  | Argentina | -23.8593 | -64.4366 | 44 | X |  |  |  |  |  |
| *E. legatus* | CML2938 |  | Pn Calie. Legatusua, Arroyo Sauzalito |  |  | Argentina | -23.6479 | -64.5821 | 45 | X |  |  |  |  |  |
| *E. legatus* | MSB63359 |  | 2 Km E Chuhuayacu | Chuquisaca |  | Bolivia | -19.7167 | -63.85 | 46 | X |  |  |  |  |  |
| *E. legatus* | MSB63360 |  | Rio Limon | Chuquisaca |  | Bolivia | -19.55 | -64.1333 | 47 | X |  |  |  |  |  |
| *E. legatus* | MSB63584 |  | Rio Limon | Chuquisaca |  | Bolivia | -19.55 | -64.1333 | 47 | X |  |  |  |  |  |
| *E. legatus* | BMNH 25. 2. 1. 24 | 1777 | Carapari [= Caraparí], 1000 M., About 35 Kilometers North Of Yacuiba, On The Way Towards Tarija | Tarifa |  | Bolivia | -21.8167 | -63.7667 | 48 | X |  |  |  |  |  |
| *E. legatus* | MSB67358 |  | Tapecua 1500 M 21D 26'S. 63D 55' W. | Tarija |  | Bolivia | -21.4333 | -63.9167 | 49 | X |  |  |  |  |  |
| *E. legatus* | MSB67359 |  | Tapecua 1500 M 21D 26'S. 63D 55' W. | Tarija |  | Bolivia | -21.4333 | -63.9167 | 49 | X |  |  |  |  |  |
| *E. legatus* | MSB67318 |  | Tapecua, 1500M. 21D 26'S. 63D 55' W. | Tarija |  | Bolivia | -21.4333 | -63.9167 | 49 | X |  |  |  |  |  |
| *E. legatus* | MSB67319 |  | Tapecua, 1500M. 21D 26'S. 63D 55' W. | Tarija |  | Bolivia | -21.4333 | -63.9167 | 49 | X |  |  |  |  |  |
| *E. legatus* | MSB67320 |  | Tapecua, 1500M. 21D 26'S. 63D 55' W. | Tarija |  | Bolivia | -21.4333 | -63.9167 | 49 | X |  |  |  |  |  |
| *E. nitidus* | MSB56050 |  | 6 Km E Estacion Biologica Del Beni, Rio Matos, 300 M | Beni |  | Bolivia | -14.51 | -66.21 | 50 | X |  |  |  |  |  |
| *E. nitidus* | MSB68448 |  | 35 Km Nw Yucumo, 253 M | Beni |  | Bolivia | -15.1 | -67.04 | 51 | X |  |  |  |  |  |
| *E. nitidus* | MSB68449 |  | 35 Km Nw Yucumo, 253 M | Beni |  | Bolivia | -15.1 | -67.04 | 51 | X |  |  |  |  |  |
| *E. nitidus* | MSB56056 |  | 45 Km N Yacuma, 400 M | Beni |  | Bolivia | -14.42 | -67.04 | 52 | X |  |  |  |  |  |
| ***E. nitidus*** | **MSB56057** |  | **45 Km N Yacuma, 400 M** | **Beni** |  | **Bolivia** | **-14.42** | **-67.04** | **52** | **X** | **X** | **MT118057** |  |  | **This study** |
| *E. nitidus* | MSB57168 |  | Boca Del Rio Biata, 170 M (11 44'S, 66 47'W) | Beni |  | Bolivia | -11.44 | -66.47 | 53 | X |  |  |  |  |  |
| *E. nitidus* | MSB57353 |  | Boca Del Rio Biata, 170M | Beni |  | Bolivia | -11.44 | -66.47 | 53 | X |  |  |  |  |  |
| *E. nitidus* | MSB57354 |  | Boca Del Rio Biata, 170M | Beni |  | Bolivia | -11.44 | -66.47 | 53 | X |  |  |  |  |  |
| *E. nitidus* | MSB68450 |  | La Reserva, 840 M | La Paz |  | Bolivia | -15.7333 | -67.5167 | 54 | X |  |  |  |  |  |
| *E. nitidus* | MSB68451 |  | La Reserva, 840 M | La Paz |  | Bolivia | -15.7333 | -67.5167 | 54 | X |  |  |  |  |  |
| *E. nitidus* | MSB68452 |  | La Reserva, 840 M | La Paz |  | Bolivia | -15.7333 | -67.5167 | 54 | X |  |  |  |  |  |
| *E. nitidus* | MSB68454 |  | La Reserva, 840 M | La Paz |  | Bolivia | -15.7333 | -67.5167 | 54 | X |  |  |  |  |  |
| *E. nitidus* | MSB68456 |  | La Reserva, 840 M | La Paz |  | Bolivia | -15.7333 | -67.5167 | 54 | X |  |  |  |  |  |
| *E. nitidus* | MSB68457 |  | La Reserva, 840 M | La Paz |  | Bolivia | -15.7333 | -67.5167 | 54 | X |  |  |  |  |  |
| *E. nitidus* | MSB68458 |  | La Reserva, 840 M | La Paz |  | Bolivia | -15.7333 | -67.5167 | 54 | X |  |  |  |  |  |
| *E. nitidus* | MSB68459 |  | La Reserva, 840 M | La Paz |  | Bolivia | -15.7333 | -67.5167 | 54 | X |  |  |  |  |  |
| *E. nitidus* | MSB68460 |  | La Reserva, 840 M | La Paz |  | Bolivia | -15.7333 | -67.5167 | 54 | X |  |  |  |  |  |
| *E. nitidus* | MSB68461 |  | La Reserva, 840 M | La Paz |  | Bolivia | -15.7333 | -67.5167 | 54 | X |  |  |  |  |  |
| *E. nitidus* | MSB68462 |  | La Reserva, 840 M | La Paz |  | Bolivia | -15.7333 | -67.5167 | 54 | X |  |  |  |  |  |
| *E. nitidus* | MSB68463 |  | La Reserva, 840 M | La Paz |  | Bolivia | -15.7333 | -67.5167 | 54 | X |  |  |  |  |  |
| *E. nitidus* | MSB68464 |  | La Reserva, 840 M | La Paz |  | Bolivia | -15.7333 | -67.5167 | 54 | X |  |  |  |  |  |
| *E. nitidus* | MSB68465 |  | La Reserva, 840 M | La Paz |  | Bolivia | -15.7333 | -67.5167 | 54 | X |  |  |  |  |  |
| *E. nitidus* | MSB68466 |  | La Reserva, 840 M | La Paz |  | Bolivia | -15.7333 | -67.5167 | 54 | X |  |  |  |  |  |
| *E. nitidus* | MSB68467 |  | La Reserva, 840 M | La Paz |  | Bolivia | -15.7333 | -67.5167 | 54 | X |  |  |  |  |  |
| *E. nitidus* | MSB68468 |  | La Reserva, 840 M | La Paz |  | Bolivia | -15.7333 | -67.5167 | 54 | X |  |  |  |  |  |
| *E. nitidus* | MSB68469 |  | La Reserva, 840 M | La Paz |  | Bolivia | -15.7333 | -67.5167 | 54 | X |  |  |  |  |  |
| *E. nitidus* | MSB68470 |  | La Reserva, 840 M | La Paz |  | Bolivia | -15.7333 | -67.5167 | 54 | X |  |  |  |  |  |
| *E. nitidus* | MSB68471 |  | La Reserva, 840 M | La Paz |  | Bolivia | -15.7333 | -67.5167 | 54 | X |  |  |  |  |  |
| *E. nitidus* | MSB68472 |  | La Reserva, 840 M | La Paz |  | Bolivia | -15.7333 | -67.5167 | 54 | X |  |  |  |  |  |
| *E. nitidus* | TTU34982 |  | 1 Mi W Puerto Liñares | La Paz |  | Bolivia | -15.29 | -67.31 | 55 | X |  |  |  |  |  |
| *E. nitidus* | TTU34983 |  | 1 Mi W Puerto Liñares | La Paz |  | Bolivia | -15.29 | -67.31 | 55 | X |  |  |  |  |  |
| *E. nitidus* | TTU34984 |  | 1 Mi W Puerto Liñares | La Paz |  | Bolivia | -15.29 | -67.31 | 55 | X |  |  |  |  |  |
| *E. nitidus* | TTU34985 |  | 1 Mi W Puerto Liñares | La Paz |  | Bolivia | -15.29 | -67.31 | 55 | X |  |  |  |  |  |
| *E. nitidus* | MSB57116 |  | Palmira | Pando |  | Bolivia | -11.42 | -67.56 | 56 | X |  |  |  |  |  |
| ***E. nitidus*** | **MSB57117** |  | **Palmira** | **Pando** |  | **Bolivia** | **-11.42** | **-67.56** | **56** | **X** | **X** | **MT118058** |  |  | **This study** |
| *E. nitidus* | MSB57169 |  | Independencia | Pando |  | Bolivia | -11.26 | -67.34 | 57 | X |  |  |  |  |  |
| *E. nitidus* | MSB55328 |  | Estancia Cachuela Esperanza | Santa Cruz |  | Bolivia | -17.7833 | -63.2333 | 58 | X |  |  |  |  |  |
| *E. nitidus* | MSB55329 |  | Estancia Cachuela Esperanza | Santa Cruz |  | Bolivia | -17.7833 | -63.2333 | 58 | X |  |  |  |  |  |
| *E. nitidus* | MSB55330 |  | Estancia Cachuela Esperanza | Santa Cruz |  | Bolivia | -17.7833 | -63.2333 | 58 | X |  |  |  |  |  |
| *E. nitidus* | MSB55331 |  | Estancia Cachuela Esperanza | Santa Cruz |  | Bolivia | -17.7833 | -63.2333 | 58 | X |  |  |  |  |  |
| *E. nitidus* | MSB55332 |  | Estancia Cachuela Esperanza | Santa Cruz |  | Bolivia | -17.7833 | -63.2333 | 58 | X |  |  |  |  |  |
| *E. nitidus* | MSB55333 |  | Estancia Cachuela Esperanza | Santa Cruz |  | Bolivia | -17.7833 | -63.2333 | 58 | X |  |  |  |  |  |
| *E. nitidus* | MSB55334 |  | Estancia Cachuela Esperanza | Santa Cruz |  | Bolivia | -17.7833 | -63.2333 | 58 | X |  |  |  |  |  |
| *E. nitidus* | MSB55335 |  | 3.5 Km W Estancion El Pailon | Santa Cruz |  | Bolivia | -17.39 | -62.45 | 59 | X |  |  |  |  |  |
| *E. nitidus* | MSB55336 |  | 3.5 Km W Estancion El Pailon | Santa Cruz |  | Bolivia | -17.39 | -62.45 | 59 | X |  |  |  |  |  |
| *E. nitidus* | MSB55337 |  | 4 Km N, 1 Km W Santiago De Chicuitos, 700 M | Santa Cruz |  | Bolivia | -18.18 | -59.36 | 60 | X |  |  |  |  |  |
| *E. nitidus* | MSB55338 |  | 4 Km N, 1 Km W Santiago De Chicuitos, 700 M | Santa Cruz |  | Bolivia | -18.18 | -59.36 | 60 | X |  |  |  |  |  |
| *E. nitidus* | MSB55339 |  | 4 Km S, 24 Km E San Jose De Chiquitos, 400 M | Santa Cruz |  | Bolivia | -17.8667 | -60.5167 | 61 | X |  |  |  |  |  |
| *E. nitidus* | MSB56062 |  | 6 Km W (By Road) Ascension, 240 M | Santa Cruz |  | Bolivia | -15.43 | -63.09 | 62 | X |  |  |  |  |  |
| *E. nitidus* | MSB56063 |  | 4.5 Km N, 1.5 Km E Cerro Amboro, Rio Pitasama, 620 M | Santa Cruz |  | Bolivia | -17.75 | -63.6667 | 63 | X |  |  |  |  |  |
| *E. nitidus* | MSB56064 |  | San Rafael De Amboro, 400 M | Santa Cruz |  | Bolivia | -17.6 | -63.6 | 64 | X |  |  |  |  |  |
| *E. nitidus* | MSB56065 |  | San Rafael De Amboro, 400 M | Santa Cruz |  | Bolivia | -17.6 | -63.6 | 64 | X |  |  |  |  |  |
| *E. nitidus* | MSB56066 |  | San Rafael De Amboro, 400 M | Santa Cruz |  | Bolivia | -17.6 | -63.6 | 64 | X |  |  |  |  |  |
| *E. nitidus* | MSB56067 |  | San Rafael De Amboro, 400 M | Santa Cruz |  | Bolivia | -17.6 | -63.6 | 64 | X |  |  |  |  |  |
| *E. nitidus* | MSB56068 |  | San Rafael De Amboro, 400 M | Santa Cruz |  | Bolivia | -17.6 | -63.6 | 64 | X |  |  |  |  |  |
| *E. nitidus* | MSB56069 |  | San Rafael De Amboro, 400 M | Santa Cruz |  | Bolivia | -17.6 | -63.6 | 64 | X |  |  |  |  |  |
| *E. nitidus* | MSB56070 |  | San Rafael De Amboro, 400 M | Santa Cruz |  | Bolivia | -17.6 | -63.6 | 64 | X |  |  |  |  |  |
| *E. nitidus* | MSB56071 |  | San Rafael De Amboro, 400 M | Santa Cruz |  | Bolivia | -17.6 | -63.6 | 64 | X |  |  |  |  |  |
| *E. nitidus* | MSB56360 |  | San Rafael De Amboro, 400 M | Santa Cruz |  | Bolivia | -17.6 | -63.6 | 64 | X |  |  |  |  |  |
| *E. nitidus* | MSB56362 |  | San Rafael De Amboro, 400 M | Santa Cruz |  | Bolivia | -17.6 | -63.6 | 64 | X |  |  |  |  |  |
| *E. nitidus* | MSB56073 |  | 10 Km N San Ramon, 250 M | Santa Cruz |  | Bolivia | -16.36 | -62.42 | 65 | X |  |  |  |  |  |
| *E. nitidus* | MSB58644 |  | 15Km S Santa Cruz | Santa Cruz |  | Bolivia | -17.54 | -63.26 | 66 | X |  |  |  |  |  |
| *E. nitidus* | MSB59878 |  | 27 Km Se Santa Cruz 17 Degree 58' S, 63 Degree 03'W, 365M | Santa Cruz |  | Bolivia | -17.9667 | -63.05 | 67 | X |  |  |  |  |  |
| *E. nitidus* | MSB59879 |  | 27 Km Se Santa Cruz 17 Degree 58' S, 63 Degree 03'W, 365M | Santa Cruz |  | Bolivia | -17.9667 | -63.05 | 67 | X |  |  |  |  |  |
| *E. nitidus* | MSB59880 |  | 27 Km Se Santa Cruz 17 Degree 58' S, 63 Degree 03'W, 365M | Santa Cruz |  | Bolivia | -17.9667 | -63.05 | 67 | X |  |  |  |  |  |
| *E. nitidus* | MSB59881 |  | 27 Km Se Santa Cruz 17 Degree 58' S, 63 Degree 03'W, 365M | Santa Cruz |  | Bolivia | -17.9667 | -63.05 | 67 | X |  |  |  |  |  |
| *E. nitidus* | MSB59882 |  | 27 Km Se Santa Cruz 17 Degree 58' S, 63 Degree 03'W, 365M | Santa Cruz |  | Bolivia | -17.9667 | -63.05 | 67 | X |  |  |  |  |  |
| *E. nitidus* | MSB63356 |  | 2 Km Sw Estancia Las Cruces | Santa Cruz |  | Bolivia | -17.47 | -63.22 | 68 | X |  |  |  |  |  |
| *E. nitidus* | MSB63357 |  | 2 Km Sw Estancia Las Cruces | Santa Cruz |  | Bolivia | -17.47 | -63.22 | 68 | X |  |  |  |  |  |
| *E. nitidus* | MSB63358 |  | 3 Km Sw Las Cruces | Santa Cruz |  | Bolivia | -17.47 | -63.22 | 68 | X |  |  |  |  |  |
| *E. nitidus* | MSB63604 |  | La Forestal | Santa Cruz |  | Bolivia |  |  |  | X |  |  |  |  |  |
| *E. nitidus* | MSB63605 |  | La Forestal | Santa Cruz |  | Bolivia |  |  |  | X |  |  |  |  |  |
| *E. nitidus* | MSB67321 |  | 1 Km Ne Estancia Cuevas, Highway Km 101 From Santa Cruz | Santa Cruz |  | Bolivia | -18.11 | -63.44 | 69 | X |  |  |  |  |  |
| *E. nitidus* | MSB67322 |  | 1 Km Ne Estancia Cuevas, Highway Km 101 From Santa Cruz | Santa Cruz |  | Bolivia | -18.11 | -63.44 | 69 | X |  |  |  |  |  |
| *E. nitidus* | MSB67323 |  | 1 Km Ne Estancia Cuevas, Highway Km 101 From Santa Cruz | Santa Cruz |  | Bolivia | -18.11 | -63.44 | 69 | X |  |  |  |  |  |
| ***E. nitidus*** | **MSB70697** | **NK22795** | **1 Km Ne Estancia Cuevas, Santa Cruz, Bolivia** | **Santa Cruz** |  | **Bolivia** | **-17.7833** | **-63.2333** | **70** |  | **X** | **MT118059** |  |  | **This study** |
| *E. nitidus* | CEM6493 |  | San Julian, Nucleo 29 | Santa Cruz De La Sierra |  | Bolivia | -16.9055 | -62.6174 | 71 | X |  |  |  |  |  |
| *E. nitidus* | AMNH262024 |  | San Rafael De Amboro | Santa Cruz De La Sierra |  | Bolivia | -17.6 | -63.6 | 72 | X |  |  |  |  |  |
| *E. nitidus* | AMNH262026 |  | San Rafael De Amboro | Santa Cruz De La Sierra |  | Bolivia | -17.6 | -63.6 | 72 | X |  |  |  |  |  |
| *E. nitidus* | AMNH262028 |  | San Rafael De Amboro | Santa Cruz De La Sierra |  | Bolivia | -17.6 | -63.6 | 72 | X |  |  |  |  |  |
| *E. nitidus* | AMNH262033 |  | San Rafael De Amboro | Santa Cruz De La Sierra |  | Bolivia | -17.6 | -63.6 | 72 | X |  |  |  |  |  |
| *E. nitidus* | AMNH262036 |  | San Rafael De Amboro | Santa Cruz De La Sierra |  | Bolivia | -17.6 | -63.6 | 72 | X |  |  |  |  |  |
| *E. nitidus* | MNFS1147 |  | Igarapé Porongaba, Right Bank Rio Juruá |  | Acre | Brazil | -8.48 | -70.36 | 73 | X |  |  |  |  |  |
| *E. nitidus* | MNFS1223 |  | Igarapé Porongaba, Right Bank Rio Juruá |  | Acre | Brazil | -8.48 | -70.36 | 73 | X |  |  |  |  |  |
| *E. nitidus* | MNFS1309 |  | Igarapé Porongaba, Right Bank Rio Juruá |  | Acre | Brazil | -8.48 | -70.36 | 73 | X |  |  |  |  |  |
| *E. nitidus* | INPA3193 | MNFS1419 | Igarapé Porongaba, Right Bank Rio Juruá |  | Acre | Brazil | -8.48 | -70.36 | 73 | X | X | U58383 |  |  | Patton et al, 2000 |
| ***E. nitidus*** | **MVZ190456** | **MNFS1420** | **Igarapé Porongaba, Right Bank Rio Juruá** |  | **Acre** | **Brazil** | **-8.48** | **-70.36** | **73** | **X** | **X** | **MT118061** |  |  | **This study** |
| *E. nitidus* | MPEG10609 |  | Manoel Urbano, Br 364 ,Km 8 | Sena Madureira | Acre | Brazil | -8.53 | -69.19 | 74 | X |  |  |  |  |  |
| *E. nitidus* | MPEG770 |  | Seringal Oriente, Proximo Vila Taumaturgo |  | Acre | Brazil | -8.53 | -69.19 | 74 | X |  |  |  |  |  |
| *E. nitidus* | MPEG771 |  | Seringal Oriente, Proximo Vila Taumaturgo |  | Acre | Brazil | -8.53 | -69.19 | 74 | X |  |  |  |  |  |
| ***E. nitidus*** |  | **M97008** | **Apiacás** | **Apiacás** |  | **Brazil** | **-9.53** | **-57.45** | **75** |  | **X** | **MT118055** |  |  | **This study** |
| ***E. nitidus*** |  | **M968409** | **Apiacás** | **Apiacás** |  | **Brazil** | **-9.53** | **-57.45** | **75** |  | **X** | **MT118056** | **X** | **MT118086** | **This study** |
| ***E. nitidus*** |  | **APC000153** | **Juruena** | **Juruena** |  | **Brazil** | **-10.32** | **-58.35** | **76** |  | **X** | **MT118052** | **X** | **MT118085** | **This study** |
| *E. nitidus* | LSUMZ16662 |  | Hacienda Luisiana, Rio Apurimac | Ayaucho |  | Peru | -12.44 | -73.47 | 77 | X |  |  |  |  |  |
| *E. nitidus* | LSUMZ16663 |  | Hacienda Luisiana, Rio Apurimac | Ayacucho |  | Peru | -12.44 | -73.47 | 77 | X |  |  |  |  |  |
| *E. nitidus* | LSUMZ16665 |  | Hacienda Luisiana, Rio Apurimac | Ayacucho |  | Peru | -12.44 | -73.47 | 77 | X |  |  |  |  |  |
| *E. nitidus* | LSUMZ16693 |  | Rio Santa Rosa, San Jose | Ayacucho |  | Peru | -12.733 | -73.767 | 78 | X |  |  |  |  |  |
| *E. nitidus* | LSUMZ19276 |  | 10 Km Rd E Quince Mil On Rio Marcapata | Cuzco |  | Peru | -13.23 | -70.43 | 79 | X |  |  |  |  |  |
| *E. nitidus* | LSUMZ19280 |  | Kiteni, 66 Km Rd From Quillabamba | Cuzco |  | Peru | -12.49 | -72.43 | 80 | X |  |  |  |  |  |
| *E. nitidus* | LSUMZ12333 |  | Balta, Rio Caranja | Loreto |  | Peru | -10.08 | -71.13 | 81 | X |  |  |  |  |  |
| *E. nitidus* | LSUMZ12334 |  | Balta, Rio Caranja | Loreto |  | Peru | -10.08 | -71.13 | 81 | X |  |  |  |  |  |
| *E. nitidus* | LSUMZ12335 |  | Balta, Rio Caranja | Loreto |  | Peru | -10.08 | -71.13 | 81 | X |  |  |  |  |  |
| *E. nitidus* | LSUMZ12336 |  | Balta, Rio Caranja | Loreto |  | Peru | -10.08 | -71.13 | 81 | X |  |  |  |  |  |
| *E. nitidus* | LSUMZ12338 |  | Balta, Rio Caranja | Loreto |  | Peru | -10.08 | -71.13 | 81 | X |  |  |  |  |  |
| *E. nitidus* | LSUMZ12339 |  | Balta, Rio Caranja | Loreto |  | Peru | -10.08 | -71.13 | 81 | X |  |  |  |  |  |
| *E. nitidus* | LSUMZ12340 |  | Balta, Rio Caranja | Loreto |  | Peru | -10.08 | -71.13 | 81 | X |  |  |  |  |  |
| *E. nitidus* | LSUMZ12341 |  | Balta, Rio Caranja | Loreto |  | Peru | -10.08 | -71.13 | 81 | X |  |  |  |  |  |
| *E. nitidus* | LSUMZ12342 |  | Balta, Rio Caranja | Loreto |  | Peru | -10.08 | -71.13 | 81 | X |  |  |  |  |  |
| *E. nitidus* | LSUMZ12343 |  | Balta, Rio Caranja | Loreto |  | Peru | -10.08 | -71.13 | 81 | X |  |  |  |  |  |
| *E. nitidus* | LSUMZ12344 |  | Balta, Rio Caranja | Loreto |  | Peru | -10.08 | -71.13 | 81 | X |  |  |  |  |  |
| *E. nitidus* | LSUMZ12345 |  | Balta, Rio Caranja | Loreto |  | Peru | -10.08 | -71.13 | 81 | X |  |  |  |  |  |
| *E. nitidus* | LSUMZ12346 |  | Balta, Rio Caranja | Loreto |  | Peru | -10.08 | -71.13 | 81 | X |  |  |  |  |  |
| *E. nitidus* | LSUMZ12347 |  | Balta, Rio Caranja | Loreto |  | Peru | -10.08 | -71.13 | 81 | X |  |  |  |  |  |
| *E. nitidus* | LSUMZ12413 |  | Balta, Rio Caranja | Loreto |  | Peru | -10.08 | -71.13 | 81 | X |  |  |  |  |  |
| *E. nitidus* | LSUMZ12414 |  | Balta, Rio Caranja | Loreto |  | Peru | -10.08 | -71.13 | 81 | X |  |  |  |  |  |
| *E. nitidus* | LSUMZ14356 |  | Balta, Rio Caranja | Loreto |  | Peru | -10.08 | -71.13 | 81 | X |  |  |  |  |  |
| *E. nitidus* | LSUMZ14357 |  | Balta, Rio Caranja | Loreto |  | Peru | -10.08 | -71.13 | 81 | X |  |  |  |  |  |
| *E. nitidus* | LSUMZ14365 |  | Balta, Rio Caranja | Loreto |  | Peru | -10.08 | -71.13 | 81 | X |  |  |  |  |  |
| *E. nitidus* | LSUMZ16690 |  | Balta, Rio Caranja | Loreto |  | Peru | -10.08 | -71.13 | 81 | X |  |  |  |  |  |
| *E. nitidus* | LSUMZ16691 |  | Balta, Rio Caranja | Loreto |  | Peru | -10.08 | -71.13 | 81 | X |  |  |  |  |  |
| *E. nitidus* | LSUMZ16694 |  | Balta, Rio Caranja | Loreto |  | Peru | -10.08 | -71.13 | 81 | X |  |  |  |  |  |
| *E. nitidus* | MVZ136573 |  | Balta, Rio Caranja | Loreto |  | Peru | -10.08 | -71.13 | 81 | X |  |  |  |  |  |
| *E. nitidus* | MVZ136574 |  | Balta, Rio Caranja | Loreto |  | Peru | -10.08 | -71.13 | 81 | X |  |  |  |  |  |
| *E. nitidus* | MVZ136576 |  | Balta, Rio Caranja | Loreto |  | Peru | -10.08 | -71.13 | 81 | X |  |  |  |  |  |
| *E. nitidus* | MVZ136578 |  | Balta, Rio Caranja | Loreto |  | Peru | -10.08 | -71.13 | 81 | X |  |  |  |  |  |
| *E. nitidus* | MVZ136588 |  | Balta, Rio Caranja | Loreto |  | Peru | -10.08 | -71.13 | 81 | X |  |  |  |  |  |
| *E. nitidus* | MVZ136616 |  | Balta, Rio Caranja | Loreto |  | Peru | -10.08 | -71.13 | 81 | X |  |  |  |  |  |
| *E. nitidus* | MVZ136616 |  | Balta, Rio Caranja | Loreto |  | Peru | -10.08 | -71.13 | 81 | X |  |  |  |  |  |
| *E. nitidus* | MVZ166028 |  | Albergue, 20 Km E Puerto Maldonado | Madre De Dios |  | Peru | -12.36 | -69.11 | 82 | X |  |  |  |  |  |
| *E. nitidus* | MVZ168961 |  | Albergue, 20 Km E Puerto Maldonado | Madre De Dios |  | Peru | -12.36 | -69.11 | 82 | X |  |  |  |  |  |
| *E. nitidus* | MVZ168962 |  | Albergue, 20 Km E Puerto Maldonado | Madre De Dios |  | Peru | -12.36 | -69.11 | 82 | X |  |  |  |  |  |
| *E. nitidus* | MVZ166028 |  | Albergue, 20 Km E Puerto Maldonado | Madre De Dios |  | Peru | -12.36 | -69.11 | 82 | X |  |  |  |  |  |
| *E. nitidus* | MVZ168961 |  | Albergue, 20 Km E Puerto Maldonado | Madre De Dios |  | Peru | -12.36 | -69.11 | 82 | X |  |  |  |  |  |
| *E. nitidus* | MVZ168962 |  | Albergue, 20 Km E Puerto Maldonado | Madre De Dios |  | Peru | -12.36 | -69.11 | 82 | X |  |  |  |  |  |
| ***E. nitidus*** |  | **CR97** | **Reserva Cusco Amazónico, 14 Km E Puerto Maldonado** |  |  | **Peru** | **-12.55** | **-69.05** | **83** |  | **X** | **MT118054** |  |  | **This study** |
| ***E. nitidus*** | **MVZ16627** | **RMW498** | **Reserva Cusco Amazónico, 14 Km E Puerto Maldonado** |  |  | **Peru** | **-12.55** | **-69.05** | **83** |  | **X** | **MT118060** |  |  | **This study** |
| ***E. emmonsae*** |  | **M97018** | **Cláudia** | **Cláudia** |  | **Brazil** | **-11.52** | **-54.88** | **84** |  | **X** | **MT118033** | **X** | **MT118076** | **This study** |
| ***E. emmonsae*** |  | **M97120** | **Cláudia** | **Cláudia** |  | **Brazil** | **-11.52** | **-54.88** | **84** |  | **X** | **MT118034** | **X** | **MT118077** | **This study** |
| ***E. emmonsae*** |  | **APC312** | **Vila Rica** | **Vila Rica** |  | **Brazil** | **-10** | **-51.1** | **85** |  | **X** | **MT118030** | **X** | **MT118074** | **This study** |
| ***E. emmonsae*** |  | **APC318** | **Vila Rica** | **Vila Rica** |  | **Brazil** | **-10** | **-51.1** | **85** |  | **X** | **MT118031** | **X** | **MT118075** | **This study** |
| ***E. emmonsae*** | **USNM549552** | **LHE536** | **Altamira** | **Vila Rica** |  | **Brazil** | **-3.65** | **-52.3667** | **86** |  | **X** | **MT118036** |  |  | **This study** |
| *E. emmonsae* | MZUSP27150 | LHE559 | Altamira | Vila Rica |  | Brazil | -3.65 | -52.3667 | 86 |  | X | AF251526 |  |  | Patton et al., 2000 |
| *E. emmonsae* | MN73840 | FMH44 | Floresta Nacional De Carajás | Parauapebas |  | Brazil |  |  |  | X |  |  |  |  |  |
| *E. emmonsae* | MN73907 | FMH170 | Floresta Nacional De Carajás | Parauapebas |  | Brazil |  |  |  | X |  |  |  |  |  |
| *E. emmonsae* | MN73923 | FMH186 | Floresta Nacional De Carajás | Parauapebas |  | Brazil |  |  |  | X |  |  |  |  |  |
| *E. emmonsae* | MN73941 | FMH204 | Floresta Nacional De Carajás | Parauapebas |  | Brazil |  |  |  | X |  |  |  |  |  |
| *E. emmonsae* | MN73952 | FMH228 | Floresta Nacional De Carajás | Parauapebas |  | Brazil |  |  |  | X |  |  |  |  |  |
| *E. emmonsae* | MN73960 | FMH237 | Floresta Nacional De Carajás | Parauapebas |  | Brazil |  |  |  | X |  |  |  |  |  |
| *E. emmonsae* | MN73856 | FMH69 | Floresta Nacional De Carajás | Parauapebas |  | Brazil |  |  |  | X |  |  |  |  |  |
| *E. emmonsae* | MN73914 | FMH177 | Floresta Nacional De Carajás | Parauapebas |  | Brazil |  |  |  | X |  |  |  |  |  |
| *E. emmonsae* | MN75372 | FMH40 | Floresta Nacional De Carajás | Parauapebas |  | Brazil |  |  |  | X |  |  |  |  |  |
| *E. emmonsae* |  | CS37 | Marabá | Marabá |  | Brazil | -5.8014 | -50.515 | 87 |  | X | AF251525 |  |  | Patton et al., 2000 |
| *E. lamia* | AMNH134644 |  | Anápolis | Anápolis | Goiás | Brazil |  |  |  | X |  |  |  |  |  |
| *E. lamia* | AMNH134663 |  | Anápolis | Anápolis | Goiás | Brazil |  |  |  | X |  |  |  |  |  |
| *E. lamia* | AMNH134664 |  | Fazenda Genipapo | Anápolis | Goiás | Brazil |  |  |  | X |  |  |  |  |  |
| *E. lamia* | AMNH134665 |  | Anápolis | Anápolis | Goiás | Brazil |  |  |  | X |  |  |  |  |  |
| *E. lamia* | AMNH134666 |  | Anápolis | Anápolis | Goiás | Brazil |  |  |  | X |  |  |  |  |  |
| *E. lamia* | AMNH134667 |  | Fazenda Anicuns | Anápolis | Goiás | Brazil |  |  |  | X |  |  |  |  |  |
| *E. lamia* | AMNH134677 |  | Anápolis | Anápolis | Goiás | Brazil |  |  |  | X |  |  |  |  |  |
| *E. lamia* | AMNH134763 |  | Anápolis | Anápolis | Goiás | Brazil |  |  |  | X |  |  |  |  |  |
| *E. lamia* | AMNH134782 |  | Anápolis | Anápolis | Goiás | Brazil |  |  |  | X |  |  |  |  |  |
| *E. lamia* | MNRJ-CRB1024 |  | Pncv, Fazenda Fiandeira | Cavalcante | Goiás | Brazil |  |  |  | X |  |  |  |  |  |
| *E. lamia* | MNRJ-CRB906 |  | Pncv, Fazenda Fiandeira | Cavalcante | Goiás | Brazil |  |  |  | X |  |  |  |  |  |
| *E. lamia* | MNRJ-CRB926 |  | Pncv, Fazenda Fiandeira | Cavalcante | Goiás | Brazil |  |  |  | X |  |  |  |  |  |
| *E. lamia* | MNRJ-CRB964 |  | Pncv, Fazenda Fiandeira | Cavalcante | Goiás | Brazil |  |  |  | X |  |  |  |  |  |
| *E. lamia* | MNRJ-CRB968 |  | Pncv, Fazenda Fiandeira | Cavalcante | Goiás | Brazil |  |  |  | X |  |  |  |  |  |
| *E. lamia* | MNRJ-CRB969 |  | Pncv, Fazenda Fiandeira | Cavalcante | Goiás | Brazil | -13.78 | -47.45 | 88 | X | X | AF181273 |  | Bonvicino et al. (1998) |  |
| *E. lamia* | MNRJ-CRB983 |  | Pncv, Fazenda Fiandeira | Cavalcante | Goiás | Brazil | -13.78 | -47.45 | 88 | X | X | GU126537 |  | Percequillo et al. (2011) |  |
| *E. lamia* | MNRJ3364 |  | Anápolis | Anápolis | Goiás | Brazil |  |  |  | X |  |  |  |  |  |
| *E. lamia* | MNRJ34187 |  | Anápolis | Anápolis | Goiás | Brazil |  |  |  | X |  |  |  |  |  |
| *E. lamia* | MNRJ34188 |  | Anápolis | Anápolis | Goiás | Brazil |  |  |  | X |  |  |  |  |  |
| *E. lamia* | MNRJ4067 |  | Anápolis | Anápolis | Goiás | Brazil |  |  |  | X |  |  |  |  |  |
| *E. lamia* | MNRJ4352 |  | Anápolis | Anápolis | Goiás | Brazil |  |  |  | X |  |  |  |  |  |
| *E. lamia* | MNRJ4354 |  | Anápolis | Anápolis | Goiás | Brazil |  |  |  | X |  |  |  |  |  |
| *E. lamia* | MNRJ4437 |  | Anápolis | Anápolis | Goiás | Brazil |  |  |  | X |  |  |  |  |  |
| *E. lamia* | MNRJ5203 |  | Anápolis | Anápolis | Goiás | Brazil |  |  |  | X |  |  |  |  |  |
| *E. macconnelli* | JLP15548 |  | Condor | Left Jurua | Amazonas | Brazil |  |  |  | X |  |  |  |  |  |
| *E. macconnelli* | JLP15549 |  | Condor | Left Jurua | Amazonas | Brazil |  |  |  | X |  |  |  |  |  |
| *E. macconnelli* | JLP15563 |  | Condor | Left Jurua | Amazonas | Brazil |  |  |  | X |  |  |  |  |  |
| *E. macconnelli* | JLP15600 |  | Condor | Left Jurua | Amazonas | Brazil |  |  |  | X |  |  |  |  |  |
| *E. macconnelli* | JLP15619 |  | Condor | Left Jurua | Amazonas | Brazil |  |  |  | X |  |  |  |  |  |
| *E. macconnelli* | JLP15859 |  | Condor | Left Jurua | Amazonas | Brazil |  |  |  | X |  |  |  |  |  |
| *E. macconnelli* | MNFS529 |  | Condor | Left Jurua | Amazonas | Brazil |  |  |  | X |  |  |  |  |  |
| *E. macconnelli* | MNFS530 |  | Condor | Left Jurua | Amazonas | Brazil |  |  |  | X |  |  |  |  |  |
| *E. macconnelli* | MNFS548 |  | Condor | Left Jurua | Amazonas | Brazil |  |  |  | X |  |  |  |  |  |
| *E. macconnelli* | MNFS549 |  | Condor | Left Jurua | Amazonas | Brazil |  |  |  | X |  |  |  |  |  |
| *E. macconnelli* | MNFS550 |  | Condor | Left Jurua | Amazonas | Brazil |  |  |  | X |  |  |  |  |  |
| *E. macconnelli* | MNFS563 |  | Condor | Left Jurua | Amazonas | Brazil |  |  |  | X |  |  |  |  |  |
| *E. macconnelli* | MNFS747 |  | Condor | Left Jurua | Amazonas | Brazil |  |  |  | X |  |  |  |  |  |
| *E. macconnelli* | JUR355 |  | Lago Vai Quem Quer | Right Jurua | Amazonas | Brazil |  |  |  | X |  |  |  |  |  |
| *E. macconnelli* | JUR393 |  | Lago Vai Quem Quer | Right Jurua | Amazonas | Brazil |  |  |  | X |  |  |  |  |  |
| *E. macconnelli* | MNRJ20887 |  | Manaus | Manaus | Amazonas | Brazil |  |  |  | X |  |  |  |  |  |
| *E. macconnelli* | MNRJ20902 |  | Manaus | Manaus | Amazonas | Brazil |  |  |  | X |  |  |  |  |  |
| *E. macconnelli* | MNRJ20903 |  | Manaus | Manaus | Amazonas | Brazil |  |  |  | X |  |  |  |  |  |
| *E. macconnelli* | MNRJ20906 |  | Manaus | Manaus | Amazonas | Brazil |  |  |  | X |  |  |  |  |  |
| *E. macconnelli* | MNRJ20907 |  | Manaus | Manaus | Amazonas | Brazil |  |  |  | X |  |  |  |  |  |
| *E. macconnelli* | MNRJ20909 |  | Manaus | Manaus | Amazonas | Brazil |  |  |  | X |  |  |  |  |  |
| *E. macconnelli* | MNRJ20915 |  | Manaus | Manaus | Amazonas | Brazil |  |  |  | X |  |  |  |  |  |
| *E. macconnelli* | MNRJ20916 |  | Manaus | Manaus | Amazonas | Brazil |  |  |  | X |  |  |  |  |  |
| *E. macconnelli* | MPEG20517 |  | Manaus | Manaus | Amazonas | Brazil |  |  |  | X |  |  |  |  |  |
| *E. macconnelli* | MPEG15105 |  | Rio Amapari | Serra Do Navio | Amapá | Brazil |  |  |  | X |  |  |  |  |  |
| *E. macconnelli* | MNRJ-M15489 |  | Serra Do Navio | Serra Do Navio | Amapá | Brazil |  |  |  | X |  |  |  |  |  |
| *E. macconnelli* | MNRJ20301 |  | Serra Do Navio | Serra Do Navio | Amapá | Brazil |  |  |  | X |  |  |  |  |  |
| *E. macconnelli* | MNRJ20302 |  | Serra Do Navio | Serra Do Navio | Amapá | Brazil |  |  |  | X |  |  |  |  |  |
| *E. macconnelli* | MNRJ20531 |  | Serra Do Navio | Serra Do Navio | Amapá | Brazil |  |  |  | X |  |  |  |  |  |
| *E. macconnelli* | MNRJ20533 |  | Serra Do Navio | Serra Do Navio | Amapá | Brazil |  |  |  | X |  |  |  |  |  |
| *E. macconnelli* | MNRJ20535 |  | Serra Do Navio | Serra Do Navio | Amapá | Brazil |  |  |  | X |  |  |  |  |  |
| *E. macconnelli* | MNRJ20536 |  | Serra Do Navio | Serra Do Navio | Amapá | Brazil |  |  |  | X |  |  |  |  |  |
| *E. macconnelli* | MNRJ20538 |  | Serra Do Navio | Serra Do Navio | Amapá | Brazil |  |  |  | X |  |  |  |  |  |
| *E. macconnelli* | MPEG15106 |  | Terezinha, Rio Amapari | Serra Do Navio | Amapá | Brazil |  |  |  | X |  |  |  |  |  |
| *E. macconnelli* | MPEG15107 |  | Serra Do Navio | Serra Do Navio | Amapá | Brazil |  |  |  | X |  |  |  |  |  |
| *E. macconnelli* | MPEG15108 |  | Serra Do Navio | Serra Do Navio | Amapá | Brazil |  |  |  | X |  |  |  |  |  |
| *E. macconnelli* | MZUSP20520 |  | Serra Do Navio | Serra Do Navio | Amapá | Brazil |  |  |  | X |  |  |  |  |  |
| *E. macconnelli* | MZUSP20521 |  | Serra Do Navio | Serra Do Navio | Amapá | Brazil |  |  |  | X |  |  |  |  |  |
| *E. macconnelli* | MPEG13163 |  | Terezinha, Rio Amapari | Serra Do Navio | Amapá | Brazil |  |  |  | X |  |  |  |  |  |
| *E. macconnelli* | MPEG15104 |  | Terezinha, Rio Amapari | Serra Do Navio | Amapá | Brazil |  |  |  | X |  |  |  |  |  |
| ***E. macconnelli*** |  | **PEU960004** | **Aripuanã, Mato Grosso, Brazil** | **Mato Grosso** |  | **Brazil** | **-10.16** | **-59.15** | **89** |  | **X** | **MT118049** | **X** | **MT118084** | **This study** |
| ***E. macconnelli*** |  | **M000147** | **Juruena, Mato Grosso, Brazil** | **Mato Grosso** |  | **Brazil** | **-10.32** | **-58.35** | **76** |  | **X** | **MT118048** | **X** | **MT118083** | **This study** |
| *E. macconnelli* | MPEG8781 |  | Altamira | Altamira | Pará | Brazil |  |  |  | X |  |  |  |  |  |
| *E. macconnelli* | MZUSP20522 |  | Br 10, Km 87-94 |  | Pará | Brazil |  |  |  | X |  |  |  |  |  |
| *E. macconnelli* | MZUSP20523 |  | Br 10, Km 87-94 |  | Pará | Brazil |  |  |  | X |  |  |  |  |  |
| *E. macconnelli* | MZUSP20524 |  | Br 10, Km 87-94 |  | Pará | Brazil |  |  |  | X |  |  |  |  |  |
| *E. macconnelli* | MZUSP20525 |  | Br 10, Km 87-94 |  | Pará | Brazil |  |  |  | X |  |  |  |  |  |
| *E. macconnelli* | MZUSP20526 |  | Br 10, Km 87-94 |  | Pará | Brazil |  |  |  | X |  |  |  |  |  |
| *E. macconnelli* | MZUSP20530 |  | Br 10, Km 87-94 |  | Pará | Brazil |  |  |  | X |  |  |  |  |  |
| *E. macconnelli* | MZUSP20531 |  | Br 10, Km 87-94 |  | Pará | Brazil |  |  |  | X |  |  |  |  |  |
| *E. macconnelli* | MZUSP20533 |  | Br 10, Km 87-94 |  | Pará | Brazil |  |  |  | X |  |  |  |  |  |
| *E. macconnelli* | MZUSP20534 |  | Br 10, Km 87-94 |  | Pará | Brazil |  |  |  | X |  |  |  |  |  |
| *E. macconnelli* | MZUSP20537 |  | Br 10, Km 87-94 |  | Pará | Brazil |  |  |  | X |  |  |  |  |  |
| *E. macconnelli* | MZUSP20538 |  | Br 10, Km 87-94 |  | Pará | Brazil |  |  |  | X |  |  |  |  |  |
| *E. macconnelli* | MZUSP21980 |  | Br 10, Km 87-94 | Serra Do Navio | Pará | Brazil |  |  |  | X |  |  |  |  |  |
| *E. macconnelli* | MZUSP21981 |  | Br 10, Km 87-94 |  | Pará | Brazil |  |  |  | X |  |  |  |  |  |
| *E. macconnelli* | MZUSP21982 |  | Br 10, Km 87-94 |  | Pará | Brazil |  |  |  | X |  |  |  |  |  |
| *E. macconnelli* | MZUSP21983 |  | Br 10, Km 87-94 |  | Pará | Brazil |  |  |  | X |  |  |  |  |  |
| *E. macconnelli* | MZUSP21984 |  | Br 10, Km 87-94 |  | Pará | Brazil |  |  |  | X |  |  |  |  |  |
| *E. macconnelli* | MZUSP21985 |  | Br 10, Km 87-94 |  | Pará | Brazil |  |  |  | X |  |  |  |  |  |
| *E. macconnelli* | MZUSP21990 |  | Br 10, Km 87-94 |  | Pará | Brazil |  |  |  | X |  |  |  |  |  |
| *E. macconnelli* | MZUSP21993 |  | Br 10, Km 87-94 |  | Pará | Brazil |  |  |  | X |  |  |  |  |  |
| *E. macconnelli* | MZUSP21995 |  | Br 10, Km 87-94 |  | Pará | Brazil |  |  |  | X |  |  |  |  |  |
| *E. macconnelli* | MZUSP21996 |  | Br 10, Km 87-94 |  | Pará | Brazil |  |  |  | X |  |  |  |  |  |
| *E. macconnelli* | MZUSP22039 |  | Br 10, Km 87-94 |  | Pará | Brazil |  |  |  | X |  |  |  |  |  |
| *E. macconnelli* | MZUSP22043 |  | Br 10, Km 87-94 | Serra Do Navio | Pará | Brazil |  |  |  | X |  |  |  |  |  |
| *E. macconnelli* | MZUSP22947 |  | Br 10, Km 87-94 |  | Pará | Brazil |  |  |  | X |  |  |  |  |  |
| *E. macconnelli* | MZUSP22950 |  | Br 10, Km 87-94 |  | Pará | Brazil |  |  |  | X |  |  |  |  |  |
| *E. macconnelli* | MZUSP22952 |  | Br 10, Km 87-94 |  | Pará | Brazil |  |  |  | X |  |  |  |  |  |
| *E. macconnelli* | MZUSP22962 |  | Br 10, Km 87-94 |  | Pará | Brazil |  |  |  | X |  |  |  |  |  |
| *E. macconnelli* | MZUSP22973 |  | Br 10, Km 87-94 |  | Pará | Brazil |  |  |  | X |  |  |  |  |  |
| *E. macconnelli* | MZUSP22975 |  | Br 10, Km 87-94 |  | Pará | Brazil |  |  |  | X |  |  |  |  |  |
| *E. macconnelli* | MZUSP22985 |  | Br 10, Km 87-94 |  | Pará | Brazil |  |  |  | X |  |  |  |  |  |
| *E. macconnelli* | MZUSP22986 |  | Br 10, Km 87-94 |  | Pará | Brazil |  |  |  | X |  |  |  |  |  |
| *E. macconnelli* | MZUSP22988 |  | Br 10, Km 87-94 |  | Pará | Brazil |  |  |  | X |  |  |  |  |  |
| *E. macconnelli* | MZUSP22990 |  | Br 10, Km 87-94 |  | Pará | Brazil |  |  |  | X |  |  |  |  |  |
| *E. macconnelli* | MZUSP22992 |  | Br 10, Km 87-94 |  | Pará | Brazil |  |  |  | X |  |  |  |  |  |
| *E. macconnelli* | MZUSP22994 |  | Br 10, Km 87-94 |  | Pará | Brazil |  |  |  | X |  |  |  |  |  |
| *E. macconnelli* | MZUSP22996 |  | Br 10, Km 87-94 |  | Pará | Brazil |  |  |  | X |  |  |  |  |  |
| *E. macconnelli* | MZUSP23031 |  | Br 10, Km 87-94 |  | Pará | Brazil |  |  |  | X |  |  |  |  |  |
| *E. macconnelli* | MZUSP23033 |  | Br 10, Km 87-94 |  | Pará | Brazil |  |  |  | X |  |  |  |  |  |
| *E. macconnelli* | MZUSP23034 |  | Br 10, Km 87-94 |  | Pará | Brazil |  |  |  | X |  |  |  |  |  |
| *E. macconnelli* | MZUSP23039 |  | Br 10, Km 87-94 |  | Pará | Brazil |  |  |  | X |  |  |  |  |  |
| *E. macconnelli* | MZUSP23040 |  | Br 10, Km 87-94 |  | Pará | Brazil |  |  |  | X |  |  |  |  |  |
| *E. macconnelli* | MZUSP23041 |  | Br 10, Km 87-94 |  | Pará | Brazil |  |  |  | X |  |  |  |  |  |
| *E. macconnelli* | MZUSP23042 |  | Br 10, Km 87-94 | Serra Do Navio | Pará | Brazil |  |  |  | X |  |  |  |  |  |
| *E. macconnelli* | MZUSP23044 |  | Br 10, Km 87-94 |  | Pará | Brazil |  |  |  | X |  |  |  |  |  |
| *E. macconnelli* | MZUSP23045 |  | Br 10, Km 87-94 |  | Pará | Brazil |  |  |  | X |  |  |  |  |  |
| *E. macconnelli* | MZUSP23046 |  | Br 10, Km 87-94 |  | Pará | Brazil |  |  |  | X |  |  |  |  |  |
| *E. macconnelli* | MZUSP23047 |  | Br 10, Km 87-94 |  | Pará | Brazil |  |  |  | X |  |  |  |  |  |
| *E. macconnelli* | MZUSP23048 |  | Br 10, Km 87-94 |  | Pará | Brazil |  |  |  | X |  |  |  |  |  |
| *E. macconnelli* | MZUSP23049 |  | Br 10, Km 87-94 |  | Pará | Brazil |  |  |  | X |  |  |  |  |  |
| *E. macconnelli* | MZUSP23050 |  | Br 10, Km 87-94 |  | Pará | Brazil |  |  |  | X |  |  |  |  |  |
| *E. macconnelli* | MZUSP23053 |  | Br 10, Km 87-94 |  | Pará | Brazil |  |  |  | X |  |  |  |  |  |
| *E. macconnelli* | MZUSP23054 |  | Br 10, Km 87-94 |  | Pará | Brazil |  |  |  | X |  |  |  |  |  |
| *E. macconnelli* | MZUSP23055 |  | Br 10, Km 87-94 |  | Pará | Brazil |  |  |  | X |  |  |  |  |  |
| *E. macconnelli* | MZUSP23056 |  | Br 10, Km 87-94 |  | Pará | Brazil |  |  |  | X |  |  |  |  |  |
| *E. macconnelli* | MZUSP23057 |  | Br 10, Km 87-94 |  | Pará | Brazil |  |  |  | X |  |  |  |  |  |
| *E. macconnelli* | MZUSP23058 |  | Br 10, Km 87-94 |  | Pará | Brazil |  |  |  | X |  |  |  |  |  |
| *E. macconnelli* | MZUSP23059 |  | Br 10, Km 87-94 |  | Pará | Brazil |  |  |  | X |  |  |  |  |  |
| *E. macconnelli* | MZUSP23060 |  | Br 10, Km 87-94 |  | Pará | Brazil |  |  |  | X |  |  |  |  |  |
| *E. macconnelli* | MZUSP23061 |  | Br 10, Km 87-94 | Serra Do Navio | Pará | Brazil |  |  |  | X |  |  |  |  |  |
| *E. macconnelli* | MZUSP23062 |  | Br 10, Km 87-94 | Serra Do Navio | Pará | Brazil |  |  |  | X |  |  |  |  |  |
| *E. macconnelli* | MZUSP23064 |  | Br 10, Km 87-94 |  | Pará | Brazil |  |  |  | X |  |  |  |  |  |
| *E. macconnelli* | MZUSP23065 |  | Br 10, Km 87-94 |  | Pará | Brazil |  |  |  | X |  |  |  |  |  |
| *E. macconnelli* | MZUSP23066 |  | Br 10, Km 87-94 |  | Pará | Brazil |  |  |  | X |  |  |  |  |  |
| *E. macconnelli* | MZUSP23068 |  | Br 10, Km 87-94 |  | Pará | Brazil |  |  |  | X |  |  |  |  |  |
| *E. macconnelli* | MZUSP23070 |  | Br 10, Km 87-94 |  | Pará | Brazil |  |  |  | X |  |  |  |  |  |
| *E. macconnelli* | MZUSP23072 |  | Br 10, Km 87-94 |  | Pará | Brazil |  |  |  | X |  |  |  |  |  |
| *E. macconnelli* | MZUSP23073 |  | Br 10, Km 87-94 |  | Pará | Brazil |  |  |  | X |  |  |  |  |  |
| *E. macconnelli* | MZUSP23075 |  | Br 10, Km 87-94 |  | Pará | Brazil |  |  |  | X |  |  |  |  |  |
| *E. macconnelli* | MZUSP23076 |  | Br 10, Km 87-94 |  | Pará | Brazil |  |  |  | X |  |  |  |  |  |
| *E. macconnelli* | MZUSP23077 |  | Br 10, Km 87-94 |  | Pará | Brazil |  |  |  | X |  |  |  |  |  |
| *E. macconnelli* | MZUSP23079 |  | Br 10, Km 87-94 |  | Pará | Brazil |  |  |  | X |  |  |  |  |  |
| *E. macconnelli* | MZUSP23080 |  | Br 10, Km 87-94 |  | Pará | Brazil |  |  |  | X |  |  |  |  |  |
| *E. macconnelli* | MZUSP23081 |  | Br 10, Km 87-94 |  | Pará | Brazil |  |  |  | X |  |  |  |  |  |
| *E. macconnelli* | MZUSP23085 |  | Br 10, Km 87-94 | Serra Do Navio | Pará | Brazil |  |  |  | X |  |  |  |  |  |
| *E. macconnelli* | MZUSP23086 |  | Br 10, Km 87-94 |  | Pará | Brazil |  |  |  | X |  |  |  |  |  |
| *E. macconnelli* | MZUSP23087 |  | Br 10, Km 87-94 |  | Pará | Brazil |  |  |  | X |  |  |  |  |  |
| *E. macconnelli* | MZUSP23088 |  | Br 10, Km 87-94 | Serra Do Navio | Pará | Brazil |  |  |  | X |  |  |  |  |  |
| *E. macconnelli* | MZUSP23093 |  | Br 10, Km 87-94 |  | Pará | Brazil |  |  |  | X |  |  |  |  |  |
| *E. macconnelli* | MZUSP23094 |  | Br 10, Km 87-94 |  | Pará | Brazil |  |  |  | X |  |  |  |  |  |
| *E. macconnelli* | MZUSP23095 |  | Br 10, Km 87-94 |  | Pará | Brazil |  |  |  | X |  |  |  |  |  |
| *E. macconnelli* | MZUSP23096 |  | Br 10, Km 87-94 |  | Pará | Brazil |  |  |  | X |  |  |  |  |  |
| *E. macconnelli* | MZUSP23097 |  | Br 10, Km 87-94 |  | Pará | Brazil |  |  |  | X |  |  |  |  |  |
| *E. macconnelli* | MZUSP23098 |  | Br 10, Km 87-94 |  | Pará | Brazil |  |  |  | X |  |  |  |  |  |
| *E. macconnelli* | MZUSP23099 |  | Br 10, Km 87-94 |  | Pará | Brazil |  |  |  | X |  |  |  |  |  |
| *E. macconnelli* | MZUSP23100 |  | Br 10, Km 87-94 |  | Pará | Brazil |  |  |  | X |  |  |  |  |  |
| *E. macconnelli* | MZUSP23101 |  | Br 10, Km 87-94 |  | Pará | Brazil |  |  |  | X |  |  |  |  |  |
| *E. macconnelli* | MZUSP23102 |  | Br 10, Km 87-94 |  | Pará | Brazil |  |  |  | X |  |  |  |  |  |
| *E. macconnelli* | MZUSP23105 |  | Br 10, Km 87-94 |  | Pará | Brazil |  |  |  | X |  |  |  |  |  |
| *E. macconnelli* | MZUSP23106 |  | Br 10, Km 87-94 |  | Pará | Brazil |  |  |  | X |  |  |  |  |  |
| *E. macconnelli* | MZUSP23109 |  | Br 10, Km 87-94 |  | Pará | Brazil |  |  |  | X |  |  |  |  |  |
| *E. macconnelli* | MZUSP23113 |  | Br 10, Km 87-94 |  | Pará | Brazil |  |  |  | X |  |  |  |  |  |
| *E. macconnelli* | MZUSP23126 |  | Br 10, Km 87-94 |  | Pará | Brazil |  |  |  | X |  |  |  |  |  |
| *E. macconnelli* | MZUSP23132 |  | Br 10, Km 87-94 |  | Pará | Brazil |  |  |  | X |  |  |  |  |  |
| *E. macconnelli* | MZUSP23140 |  | Br 10, Km 87-94 |  | Pará | Brazil |  |  |  | X |  |  |  |  |  |
| *E. macconnelli* | MZUSP23142 |  | Br 10, Km 87-94 |  | Pará | Brazil |  |  |  | X |  |  |  |  |  |
| *E. macconnelli* | MZUSP23184 |  | Br 10, Km 87-94 |  | Pará | Brazil |  |  |  | X |  |  |  |  |  |
| *E. macconnelli* | MZUSP23204 |  | Br 10, Km 87-94 |  | Pará | Brazil |  |  |  | X |  |  |  |  |  |
| *E. macconnelli* | MZUSP23246 |  | Br 10, Km 87-94 |  | Pará | Brazil |  |  |  | X |  |  |  |  |  |
| *E. macconnelli* | MZUSP23248 |  | Br 10, Km 87-94 |  | Pará | Brazil |  |  |  | X |  |  |  |  |  |
| *E. macconnelli* | MZUSP23252 |  | Br 10, Km 87-94 |  | Pará | Brazil |  |  |  | X |  |  |  |  |  |
| *E. macconnelli* | MZUSP23256 |  | Br 10, Km 87-94 |  | Pará | Brazil |  |  |  | X |  |  |  |  |  |
| *E. macconnelli* | MZUSP23265 |  | Br 10, Km 87-94 |  | Pará | Brazil |  |  |  | X |  |  |  |  |  |
| *E. macconnelli* | MZUSP23274 |  | Br 10, Km 87-94 |  | Pará | Brazil |  |  |  | X |  |  |  |  |  |
| *E. macconnelli* | MZUSP23293 |  | Br 10, Km 87-94 |  | Pará | Brazil |  |  |  | X |  |  |  |  |  |
| *E. macconnelli* | MZUSP23300 |  | Br 10, Km 87-94 |  | Pará | Brazil |  |  |  | X |  |  |  |  |  |
| *E. macconnelli* | MZUSP23302 |  | Br 10, Km 87-94 |  | Pará | Brazil |  |  |  | X |  |  |  |  |  |
| *E. macconnelli* | MZUSP23332 |  | Br 10, Km 87-94 |  | Pará | Brazil |  |  |  | X |  |  |  |  |  |
| *E. macconnelli* | MZUSP23377 |  | Br 10, Km 87-94 |  | Pará | Brazil |  |  |  | X |  |  |  |  |  |
| *E. macconnelli* | MZUSP23379 |  | Br 10, Km 87-94 |  | Pará | Brazil |  |  |  | X |  |  |  |  |  |
| *E. macconnelli* | MZUSP23425 |  | Br 10, Km 87-94 |  | Pará | Brazil |  |  |  | X |  |  |  |  |  |
| *E. macconnelli* | MZUSP23428 |  | Br 10, Km 87-94 |  | Pará | Brazil |  |  |  | X |  |  |  |  |  |
| *E. macconnelli* | MZUSP23430 |  | Br 10, Km 87-94 |  | Pará | Brazil |  |  |  | X |  |  |  |  |  |
| *E. macconnelli* | MZUSP23431 |  | Br 10, Km 87-94 |  | Pará | Brazil |  |  |  | X |  |  |  |  |  |
| *E. macconnelli* | MZUSP23438 |  | Br 10, Km 87-94 |  | Pará | Brazil |  |  |  | X |  |  |  |  |  |
| *E. macconnelli* | MZUSP23441 |  | Br 10, Km 87-94 |  | Pará | Brazil |  |  |  | X |  |  |  |  |  |
| *E. macconnelli* | MZUSP23445 |  | Br 10, Km 87-94 |  | Pará | Brazil |  |  |  | X |  |  |  |  |  |
| *E. macconnelli* | MZUSP23446 |  | Br 10, Km 87-94 |  | Pará | Brazil |  |  |  | X |  |  |  |  |  |
| *E. macconnelli* | MZUSP23447 |  | Br 10, Km 87-94 |  | Pará | Brazil |  |  |  | X |  |  |  |  |  |
| *E. macconnelli* | MZUSP23450 |  | Br 10, Km 87-94 |  | Pará | Brazil |  |  |  | X |  |  |  |  |  |
| *E. macconnelli* | MZUSP23451 |  | Br 10, Km 87-94 | Serra Do Navio | Pará | Brazil |  |  |  | X |  |  |  |  |  |
| *E. macconnelli* | MZUSP23452 |  | Br 10, Km 87-94 |  | Pará | Brazil |  |  |  | X |  |  |  |  |  |
| *E. macconnelli* | MZUSP23454 |  | Br 10, Km 87-94 |  | Pará | Brazil |  |  |  | X |  |  |  |  |  |
| *E. macconnelli* | MZUSP23455 |  | Br 10, Km 87-94 |  | Pará | Brazil |  |  |  | X |  |  |  |  |  |
| *E. macconnelli* | MZUSP23457 |  | Br 10, Km 87-94 | Serra Do Navio | Pará | Brazil |  |  |  | X |  |  |  |  |  |
| *E. macconnelli* | MZUSP23458 |  | Br 10, Km 87-94 |  | Pará | Brazil |  |  |  | X |  |  |  |  |  |
| *E. macconnelli* | MZUSP23459 |  | Br 10, Km 87-94 | Serra Do Navio | Pará | Brazil |  |  |  | X |  |  |  |  |  |
| *E. macconnelli* | MZUSP23461 |  | Br 10, Km 87-94 |  | Pará | Brazil |  |  |  | X |  |  |  |  |  |
| *E. macconnelli* | MZUSP23464 |  | Br 10, Km 87-94 |  | Pará | Brazil |  |  |  | X |  |  |  |  |  |
| *E. macconnelli* | MZUSP23467 |  | Br 10, Km 87-94 |  | Pará | Brazil |  |  |  | X |  |  |  |  |  |
| *E. macconnelli* | MZUSP23470 |  | Br 10, Km 87-94 |  | Pará | Brazil |  |  |  | X |  |  |  |  |  |
| *E. macconnelli* | MZUSP23475 |  | Br 10, Km 87-94 |  | Pará | Brazil |  |  |  | X |  |  |  |  |  |
| *E. macconnelli* | MZUSP23476 |  | Br 10, Km 87-94 |  | Pará | Brazil |  |  |  | X |  |  |  |  |  |
| *E. macconnelli* | MZUSP23477 |  | Br 10, Km 87-94 |  | Pará | Brazil |  |  |  | X |  |  |  |  |  |
| *E. macconnelli* | MZUSP23480 |  | Br 10, Km 87-94 |  | Pará | Brazil |  |  |  | X |  |  |  |  |  |
| *E. macconnelli* | MPEG8208 |  | Br 14, Km 87-94, Capim |  | Pará | Brazil |  |  |  | X |  |  |  |  |  |
| *E. macconnelli* | MPEG8211 |  | Br 14, Km 87-94, Capim |  | Pará | Brazil |  |  |  | X |  |  |  |  |  |
| *E. macconnelli* | MPEG8212 |  | Br 14, Km 87-94, Capim |  | Pará | Brazil |  |  |  | X |  |  |  |  |  |
| *E. macconnelli* | MPEG8213 |  | Br 14, Km 87-94, Capim |  | Pará | Brazil |  |  |  | X |  |  |  |  |  |
| *E. macconnelli* | MPEG8214 |  | Br 14, Km 87-94, Capim |  | Pará | Brazil |  |  |  | X |  |  |  |  |  |
| *E. macconnelli* | MPEG8215 |  | Br 14, Km 87-94, Capim |  | Pará | Brazil |  |  |  | X |  |  |  |  |  |
| *E. macconnelli* | MPEG8216 |  | Br 14, Km 87-94, Capim |  | Pará | Brazil |  |  |  | X |  |  |  |  |  |
| *E. macconnelli* | MPEG8217 |  | Br 14, Km 87-94, Capim |  | Pará | Brazil |  |  |  | X |  |  |  |  |  |
| *E. macconnelli* | MPEG8218 |  | Br 14, Km 87-94, Capim |  | Pará | Brazil |  |  |  | X |  |  |  |  |  |
| *E. macconnelli* | MPEG8219 |  | Br 14, Km 87-94, Capim |  | Pará | Brazil |  |  |  | X |  |  |  |  |  |
| *E. macconnelli* | MPEG8220 |  | Br 14, Km 87-94, Capim |  | Pará | Brazil |  |  |  | X |  |  |  |  |  |
| *E. macconnelli* | MPEG8221 |  | Br 14, Km 87-94, Capim |  | Pará | Brazil |  |  |  | X |  |  |  |  |  |
| *E. macconnelli* | MPEG8252 |  | Br 14, Km 87-94, Capim |  | Pará | Brazil |  |  |  | X |  |  |  |  |  |
| *E. macconnelli* | MPEG8253 |  | Br 14, Km 87-94, Capim |  | Pará | Brazil |  |  |  | X |  |  |  |  |  |
| *E. macconnelli* | MPEG8256 |  | Br 14, Km 87-94, Capim |  | Pará | Brazil |  |  |  | X |  |  |  |  |  |
| *E. macconnelli* | MPEG8410 |  | Br 14, Km 87-94, Capim |  | Pará | Brazil |  |  |  | X |  |  |  |  |  |
| *E. macconnelli* | MPEG8422 |  | Br 14, Km 87-94, Capim |  | Pará | Brazil |  |  |  | X |  |  |  |  |  |
| *E. macconnelli* | MPEG15109 |  | Br 14, Km 87-94, Capim |  | Pará | Brazil |  |  |  | X |  |  |  |  |  |
| *E. macconnelli* | MPEG15110 |  | Br 14, Km 87-94, Capim |  | Pará | Brazil |  |  |  | X |  |  |  |  |  |
| *E. macconnelli* | MPEG15111 |  | Br 14, Km 87-94, Capim |  | Pará | Brazil |  |  |  | X |  |  |  |  |  |
| *E. macconnelli* | MPEG8224 |  | Br 165, Est. Santarem Cuiaba, Km 216-217 |  | Pará | Brazil |  |  |  | X |  |  |  |  |  |
| *E. macconnelli* | MPEG8225 |  | Br 165, Est. Santarem Cuiaba, Km 216-217 |  | Pará | Brazil |  |  |  | X |  |  |  |  |  |
| *E. macconnelli* | MPEG8226 |  | Br 165, Est. Santarem Cuiaba, Km 216-217 |  | Pará | Brazil |  |  |  | X |  |  |  |  |  |
| *E. macconnelli* | MPEG8227 |  | Br 165, Est. Santarem Cuiaba, Km 216-217 |  | Pará | Brazil |  |  |  | X |  |  |  |  |  |
| *E. macconnelli* | MPEG8228 |  | Br 165, Est. Santarem Cuiaba, Km 216-217 |  | Pará | Brazil |  |  |  | X |  |  |  |  |  |
| *E. macconnelli* | MPEG15113 |  | Br 165, Est. Santarem Cuiaba, Km 216-217 |  | Pará | Brazil |  |  |  | X |  |  |  |  |  |
| *E. macconnelli* | MPEG15114 |  | Br 165, Est. Santarem Cuiaba, Km 216-217 |  | Pará | Brazil |  |  |  | X |  |  |  |  |  |
| *E. macconnelli* | MPEG15115 |  | Br 165, Est. Santarem Cuiaba, Km 216-217 |  | Pará | Brazil |  |  |  | X |  |  |  |  |  |
| *E. macconnelli* | MPEG15116 |  | Br 165, Est. Santarem Cuiaba, Km 216-217 |  | Pará | Brazil |  |  |  | X |  |  |  |  |  |
| *E. macconnelli* | MPEG15117 |  | Br 165, Est. Santarem Cuiaba, Km 216-217 |  | Pará | Brazil |  |  |  | X |  |  |  |  |  |
| *E. macconnelli* | MPEG15118 |  | Br 165, Est. Santarem Cuiaba, Km 216-217 |  | Pará | Brazil |  |  |  | X |  |  |  |  |  |
| *E. macconnelli* | MPEG15122 |  | Itaituba | Itaituba | Pará | Brazil |  |  |  | X |  |  |  |  |  |
| *E. macconnelli* | MPEG20136 |  | Itupiranga, 26Kmn/30Kmw Maraba, Gl.5 Lt.5, 05.06S/49.24W | Maraba | Pará | Brazil |  |  |  | X |  |  |  |  |  |
| *E. macconnelli* | M8271 |  | Utinga | Belém | Pará | Brazil |  |  |  | X |  |  |  |  |  |
| *E. macconnelli* | M8312 |  | Utinga | Belém | Pará | Brazil |  |  |  | X |  |  |  |  |  |
| *E. macconnelli* | M8523 |  | Utinga | Belém | Pará | Brazil |  |  |  | X |  |  |  |  |  |
| *E. macconnelli* | MNRJ29495 |  | Projeto Jari | Monte Dourado | Pará | Brazil |  |  |  | X |  |  |  |  |  |
| *E. macconnelli* | MNRJ29496 |  | Projeto Jari | Monte Dourado | Pará | Brazil |  |  |  | X |  |  |  |  |  |
| *E. macconnelli* | MNRJ29498 |  | Projeto Jari | Monte Dourado | Pará | Brazil |  |  |  | X |  |  |  |  |  |
| *E. macconnelli* | MNRJ29499 |  | Projeto Jari | Monte Dourado | Pará | Brazil |  |  |  | X |  |  |  |  |  |
| *E. macconnelli* | MNRJ29501 |  | Projeto Jari | Monte Dourado | Pará | Brazil |  |  |  | X |  |  |  |  |  |
| *E. macconnelli* | MNRJ29502 |  | Projeto Jari | Monte Dourado | Pará | Brazil |  |  |  | X |  |  |  |  |  |
| *E. macconnelli* | MNRJ29503 |  | Projeto Jari | Monte Dourado | Pará | Brazil |  |  |  | X |  |  |  |  |  |
| *E. macconnelli* | MNRJ29504 |  | Projeto Jari | Monte Dourado | Pará | Brazil |  |  |  | X |  |  |  |  |  |
| *E. macconnelli* | MNRJ29505 |  | Projeto Jari | Monte Dourado | Pará | Brazil |  |  |  | X |  |  |  |  |  |
| *E. macconnelli* | MNRJ29507 |  | Projeto Jari | Monte Dourado | Pará | Brazil |  |  |  | X |  |  |  |  |  |
| *E. macconnelli* | MNRJ29509 |  | Projeto Jari | Monte Dourado | Pará | Brazil |  |  |  | X |  |  |  |  |  |
| *E. macconnelli* | MNRJ29510 |  | Projeto Jari | Monte Dourado | Pará | Brazil |  |  |  | X |  |  |  |  |  |
| *E. macconnelli* | MNRJ29520 |  | Projeto Jari | Monte Dourado | Pará | Brazil |  |  |  | X |  |  |  |  |  |
| *E. macconnelli* | MNRJ29522 |  | Projeto Jari | Monte Dourado | Pará | Brazil |  |  |  | X |  |  |  |  |  |
| *E. macconnelli* | MNRJ29525 |  | Projeto Jari | Monte Dourado | Pará | Brazil |  |  |  | X |  |  |  |  |  |
| *E. macconnelli* | MNRJ29526 |  | Projeto Jari | Monte Dourado | Pará | Brazil |  |  |  | X |  |  |  |  |  |
| *E. macconnelli* | MNRJ29532 |  | Projeto Jari | Monte Dourado | Pará | Brazil |  |  |  | X |  |  |  |  |  |
| *E. macconnelli* | MNRJ29533 |  | Projeto Jari | Monte Dourado | Pará | Brazil |  |  |  | X |  |  |  |  |  |
| *E. macconnelli* | MNRJ29536 |  | Projeto Jari | Monte Dourado | Pará | Brazil |  |  |  | X |  |  |  |  |  |
| *E. macconnelli* | MNRJ29539 |  | Projeto Jari | Monte Dourado | Pará | Brazil |  |  |  | X |  |  |  |  |  |
| *E. macconnelli* | MNRJ29542 |  | Projeto Jari | Monte Dourado | Pará | Brazil |  |  |  | X |  |  |  |  |  |
| *E. macconnelli* | MNRJ29547 |  | Projeto Jari | Monte Dourado | Pará | Brazil |  |  |  | X |  |  |  |  |  |
| *E. macconnelli* | MNRJ29548 |  | Projeto Jari | Monte Dourado | Pará | Brazil |  |  |  | X |  |  |  |  |  |
| *E. macconnelli* | MNRJ29558 |  | Projeto Jari | Monte Dourado | Pará | Brazil |  |  |  | X |  |  |  |  |  |
| *E. macconnelli* | MNRJ29559 |  | Projeto Jari | Monte Dourado | Pará | Brazil |  |  |  | X |  |  |  |  |  |
| *E. macconnelli* | MNRJ29560 |  | Projeto Jari | Monte Dourado | Pará | Brazil |  |  |  | X |  |  |  |  |  |
| *E. macconnelli* | MNRJ29561 |  | Projeto Jari | Monte Dourado | Pará | Brazil |  |  |  | X |  |  |  |  |  |
| *E. macconnelli* | MNRJ29564 |  | Projeto Jari | Monte Dourado | Pará | Brazil |  |  |  | X |  |  |  |  |  |
| *E. macconnelli* | MNRJ29570 |  | Projeto Jari | Monte Dourado | Pará | Brazil |  |  |  | X |  |  |  |  |  |
| *E. macconnelli* | MNRJ29571 |  | Projeto Jari | Monte Dourado | Pará | Brazil |  |  |  | X |  |  |  |  |  |
| *E. macconnelli* | MNRJ29573 |  | Projeto Jari | Monte Dourado | Pará | Brazil |  |  |  | X |  |  |  |  |  |
| *E. macconnelli* | MNRJ29585 |  | Projeto Jari | Monte Dourado | Pará | Brazil |  |  |  | X |  |  |  |  |  |
| *E. macconnelli* | MNRJ29589 |  | Projeto Jari | Monte Dourado | Pará | Brazil |  |  |  | X |  |  |  |  |  |
| *E. macconnelli* | MNRJ29591 |  | Projeto Jari | Monte Dourado | Pará | Brazil |  |  |  | X |  |  |  |  |  |
| *E. macconnelli* | MNRJ29592 |  | Projeto Jari | Monte Dourado | Pará | Brazil |  |  |  | X |  |  |  |  |  |
| *E. macconnelli* | MPEG10111 |  | Porto Trombetas, Rio Saracazinho, Km7 | Oriximina | Pará | Brazil |  |  |  | X |  |  |  |  |  |
| *E. macconnelli* | MPEG15101 |  | Serra Norte | Maraba | Pará | Brazil |  |  |  | X |  |  |  |  |  |
| *E. macconnelli* | MPEG15103 |  | Serra Norte | Maraba | Pará | Brazil |  |  |  | X |  |  |  |  |  |
| *E. macconnelli* | MPEG8184 |  | Serra Norte | Maraba | Pará | Brazil |  |  |  | X |  |  |  |  |  |
| *E. macconnelli* | MPEG8188 |  | Serra Norte | Maraba | Pará | Brazil |  |  |  | X |  |  |  |  |  |
| *E. macconnelli* | MPEG8189 |  | Serra Norte | Maraba | Pará | Brazil |  |  |  | X |  |  |  |  |  |
| *E. macconnelli* | MPEG8190 |  | Serra Norte | Maraba | Pará | Brazil |  |  |  | X |  |  |  |  |  |
| *E. macconnelli* | MPEG8191 |  | Serra Norte | Maraba | Pará | Brazil |  |  |  | X |  |  |  |  |  |
| *E. macconnelli* | MPEG8192 |  | Serra Norte | Maraba | Pará | Brazil |  |  |  | X |  |  |  |  |  |
| *E. macconnelli* | MPEG8193 |  | Serra Norte | Maraba | Pará | Brazil |  |  |  | X |  |  |  |  |  |
| *E. macconnelli* | MPEG8199 |  | Serra Norte | Maraba | Pará | Brazil |  |  |  | X |  |  |  |  |  |
| *E. macconnelli* | MPEG8207 |  | Serra Norte | Maraba | Pará | Brazil |  |  |  | X |  |  |  |  |  |
| *E. macconnelli* | MPEG8655 |  | Serra Norte | Maraba | Pará | Brazil |  |  |  | X |  |  |  |  |  |
| *E. macconnelli* | MPEG8656 |  | Serra Norte | Maraba | Pará | Brazil |  |  |  | X |  |  |  |  |  |
| *E. macconnelli* | MPEG8658 |  | Serra Norte | Maraba | Pará | Brazil |  |  |  | X |  |  |  |  |  |
| *E. macconnelli* | MPEG8659 |  | Serra Norte | Maraba | Pará | Brazil |  |  |  | X |  |  |  |  |  |
| *E. macconnelli* | MPEG8775 |  | Serra Norte | Maraba | Pará | Brazil |  |  |  | X |  |  |  |  |  |
| ***E. macconnelli*** |  | **CS32** | **Marabá** | **Marabá** |  | **Brazil** | **-5.8014** | **-50.515** | **87** |  | **X** | **MT118045** |  |  | **This study** |
| *E. macconnelli* | MPEG2470 |  | Utinga | Belém | Pará | Brazil |  |  |  | X |  |  |  |  |  |
| *E. macconnelli* | MPEG2471 |  | Utinga | Belém | Pará | Brazil |  |  |  | X |  |  |  |  |  |
| *E. macconnelli* | MPEG2476 |  | Utinga | Belém | Pará | Brazil |  |  |  | X |  |  |  |  |  |
| *E. macconnelli* | MPEG2588 |  | Utinga | Belém | Pará | Brazil |  |  |  | X |  |  |  |  |  |
| *E. macconnelli* | MPEG2601 |  | Utinga | Belém | Pará | Brazil |  |  |  | X |  |  |  |  |  |
| *E. macconnelli* | MPEG2611 |  | Utinga | Belém | Pará | Brazil |  |  |  | X |  |  |  |  |  |
| *E. macconnelli* | MPEG2622 |  | Utinga | Belém | Pará | Brazil |  |  |  | X |  |  |  |  |  |
| *E. macconnelli* | MPEG2624 |  | Utinga | Belém | Pará | Brazil |  |  |  | X |  |  |  |  |  |
| *E. macconnelli* | MPEG2633 |  | Utinga | Belém | Pará | Brazil |  |  |  | X |  |  |  |  |  |
| *E. macconnelli* | MPEG2644 |  | Utinga | Belém | Pará | Brazil |  |  |  | X |  |  |  |  |  |
| *E. macconnelli* | MNRJ27957 |  | Rio Jamari, Uhe | Samuel | Rondônia | Brazil |  |  |  | X |  |  |  |  |  |
| *E. macconnelli* | MNRJ11664 |  | Rio Capihuara | Oriente |  | Equador |  |  |  | X |  |  |  |  |  |
| *E. macconnelli* | MVZ153516 |  | 0.5 Mi S Huampani, Rio Cenepa | Amazonas |  | Peru |  |  |  | X |  |  |  |  |  |
| ***E. macconnelli*** |  | **LLW447** | **2 Km Sw Tangoshiari** |  |  | **Peru** | **-11.7797** | **-73.3407** | **90** |  | **X** | **MT118046** |  |  | **This study** |
| ***E. macconnelli*** |  | **LLW462** | **2 Km Sw Tangoshiari** |  |  | **Peru** | **-11.7797** | **-73.3407** | **90** |  | **X** | **MT118047** |  |  | **This study** |
| *E. macconnelli* | LSUMZ14366 |  | Balta, Rio Caranja | Loreto |  | Peru |  |  |  | X |  |  |  |  |  |
| *E. macconnelli* | MVZ154972 |  | Headwaters Of Rio Kagka (Of Rio Cenepa) | Amazonas |  | Peru |  |  |  | X |  |  |  |  |  |
| *E. macconnelli* | MVZ154974 |  | Headwaters Of Rio Kagka (Of Rio Cenepa) | Amazonas |  | Peru |  |  |  | X |  |  |  |  |  |
| ***E. macconnelli*** |  | **RSV2025** | **Nuevo San Juan, Río Gálvez** |  |  | **Peru** | **-5.25** | **-73.16** | **91** |  | **X** | **MT118050** |  |  | **This study** |
| ***E. macconnelli*** |  | **RSV2030** | **Nuevo San Juan, Río Gálvez** |  |  | **Peru** | **-5.25** | **-73.16** | **91** |  | **X** | **MT118051** |  |  | **This study** |
| *E. macconnelli* | AMNH272678 |  | Río Gálvez |  |  | Peru | -5.12 | -72.53 | 92 |  | X | EU579484 |  |  | Hanson and Bradley, unpublished |
| *E. macconnelli* | AMNH130880 |  | Auyantepui | Auyantepui |  | Venezuela |  |  |  | X |  |  |  |  |  |
| *E. macconnelli* | AMNH130908 |  | Auyantepui | Auyantepui |  | Venezuela |  |  |  | X |  |  |  |  |  |
| *E. macconnelli* | AMNH130913 |  | Auyantepui | Auyantepui |  | Venezuela |  |  |  | X |  |  |  |  |  |
| *E. macconnelli* | AMNH130928 |  | Auyantepui | Auyantepui |  | Venezuela |  |  |  | X |  |  |  |  |  |
| *E. macconnelli* | AMNH130975 |  | Auyantepui | Auyantepui |  | Venezuela |  |  |  | X |  |  |  |  |  |
| *E. macconnelli* | AMNH130976 |  | Auyantepui | Auyantepui |  | Venezuela |  |  |  | X |  |  |  |  |  |
| ***E. macconnelli*** | **CMNH64561** | **TK11324** | **1.5 Km W Rudi, Kappel Vliegveld** |  |  | **Suriname** | **3.7833** | **-56.1667** | **93** |  | **X** | **MT118044** |  |  | **This study** |
| *E. russatus* | CEM#49 |  | 15 Km N Ruta 19 Y Rio Uruzu | Iguazu |  | Argentina |  |  |  | X |  |  |  |  |  |
| *E. russatus* | CEM2963 |  | Bonpland |  |  | Argentina |  |  |  | X |  |  |  |  |  |
| *E. russatus* | CEM4533 |  | Cuartel R. Victoria | Guarani |  | Argentina |  |  |  | X |  |  |  |  |  |
| *E. russatus* | CEM4984 |  | Cuartel R. Victoria | Guarani |  | Argentina |  |  |  | X |  |  |  |  |  |
| *E. russatus* | CJC2860 |  | Puerto Peninsula | Iguazu |  | Argentina |  |  |  | X |  |  |  |  |  |
| *E. russatus* | MACN18504 |  | P.N.Iguazu, Estancia San Jorge | Iguazu |  | Argentina |  |  |  | X |  |  |  |  |  |
| *E. russatus* | MACN18888 |  | Santa Victoria | Gral. Belgrano |  | Argentina |  |  |  | X |  |  |  |  |  |
| *E. russatus* | MACN18889 |  | Santa Victoria | Gral. Belgrano |  | Argentina |  |  |  | X |  |  |  |  |  |
| *E. russatus* | MNRJ-M23417 |  | Aritágua | Ilhéus | Bahia | Brazil |  |  |  | X |  |  |  |  |  |
| *E. russatus* | MNRJ-M23721 |  | Aritágua | Ilhéus | Bahia | Brazil |  |  |  | X |  |  |  |  |  |
| *E. russatus* | MNRJ-M23905 |  | Aritágua | Ilhéus | Bahia | Brazil |  |  |  | X |  |  |  |  |  |
| *E. russatus* | UFMG-MAS37 |  | Aritágua | Ilhéus | Bahia | Brazil |  |  |  | X |  |  |  |  |  |
| *E. russatus* | MNRJ30597 |  | Aritágua | Ilhéus | Bahia | Brazil |  |  |  | X |  |  |  |  |  |
| *E. russatus* | MNRJ8886 |  | Aritágua | Ilhéus | Bahia | Brazil |  |  |  | X |  |  |  |  |  |
| *E. russatus* | MNRJ8888 |  | Aritágua | Ilhéus | Bahia | Brazil |  |  |  | X |  |  |  |  |  |
| *E. russatus* | MNRJ8901 |  | Aritágua | Ilhéus | Bahia | Brazil |  |  |  | X |  |  |  |  |  |
| *E. russatus* | MNRJ9003 |  | Aritágua | Ilhéus | Bahia | Brazil |  |  |  | X |  |  |  |  |  |
| *E. russatus* | MNRJ9007 |  | Aritágua | Ilhéus | Bahia | Brazil |  |  |  | X |  |  |  |  |  |
| *E. russatus* | MNRJ9017 |  | Aritágua | Ilhéus | Bahia | Brazil |  |  |  | X |  |  |  |  |  |
| *E. russatus* | MNRJ9018 |  | Aritágua | Ilhéus | Bahia | Brazil |  |  |  | X |  |  |  |  |  |
| *E. russatus* | MNRJ9025 |  | Aritágua | Ilhéus | Bahia | Brazil |  |  |  | X |  |  |  |  |  |
| *E. russatus* | UFRJ9032 |  | Aritágua | Ilhéus | Bahia | Brazil |  |  |  | X |  |  |  |  |  |
| *E. russatus* | MNRJ9077 |  | Aritágua | Ilhéus | Bahia | Brazil |  |  |  | X |  |  |  |  |  |
| *E. russatus* | MNRJ9093 |  | Aritágua | Ilhéus | Bahia | Brazil |  |  |  | X |  |  |  |  |  |
| *E. russatus* | MNRJ9097 |  | Aritágua | Ilhéus | Bahia | Brazil |  |  |  | X |  |  |  |  |  |
| *E. russatus* | MNRJ9099 |  | Aritágua | Ilhéus | Bahia | Brazil |  |  |  | X |  |  |  |  |  |
| *E. russatus* | MNRJ9102 |  | Aritágua | Ilhéus | Bahia | Brazil |  |  |  | X |  |  |  |  |  |
| *E. russatus* | MNRJ9104 |  | Aritágua | Ilhéus | Bahia | Brazil |  |  |  | X |  |  |  |  |  |
| *E. russatus* | MNRJ9105 |  | Aritágua | Ilhéus | Bahia | Brazil |  |  |  | X |  |  |  |  |  |
| *E. russatus* | MNRJ9108 |  | Aritágua | Ilhéus | Bahia | Brazil |  |  |  | X |  |  |  |  |  |
| *E. russatus* | MNRJ9119 |  | Aritágua | Ilhéus | Bahia | Brazil |  |  |  | X |  |  |  |  |  |
| *E. russatus* | MNRJ9184 |  | Aritágua | Ilhéus | Bahia | Brazil |  |  |  | X |  |  |  |  |  |
| *E. russatus* | MNRJ9197 |  | Aritágua | Ilhéus | Bahia | Brazil |  |  |  | X |  |  |  |  |  |
| *E. russatus* | MNRJ9206 |  | Aritágua | Ilhéus | Bahia | Brazil |  |  |  | X |  |  |  |  |  |
| *E. russatus* | MNRJ9267 |  | Aritágua | Ilhéus | Bahia | Brazil |  |  |  | X |  |  |  |  |  |
| *E. russatus* | MNRJ9273 |  | Banco Da Vitória | Ilhéus | Bahia | Brazil |  |  |  | X |  |  |  |  |  |
| *E. russatus* | MNRJ9275 |  | Banco Da Vitória | Ilhéus | Bahia | Brazil |  |  |  | X |  |  |  |  |  |
| *E. russatus* | MNRJ9278 |  | Banco Da Vitória | Ilhéus | Bahia | Brazil |  |  |  | X |  |  |  |  |  |
| *E. russatus* | UFRJ9283 |  | Banco Da Vitória | Ilhéus | Bahia | Brazil |  |  |  | X |  |  |  |  |  |
| *E. russatus* | MNRJ9284 |  | Banco Da Vitória | Ilhéus | Bahia | Brazil |  |  |  | X |  |  |  |  |  |
| *E. russatus* | MNRJ9285 |  | Banco Da Vitória | Ilhéus | Bahia | Brazil |  |  |  | X |  |  |  |  |  |
| *E. russatus* | MNRJ9288 |  | Banco Da Vitória | Ilhéus | Bahia | Brazil |  |  |  | X |  |  |  |  |  |
| *E. russatus* | MNRJ9307 |  | Banco Da Vitória | Ilhéus | Bahia | Brazil |  |  |  | X |  |  |  |  |  |
| *E. russatus* | MNRJ9312 |  | Banco Da Vitória | Ilhéus | Bahia | Brazil |  |  |  | X |  |  |  |  |  |
| *E. russatus* | MNRJ9328 |  | Banco Da Vitória | Ilhéus | Bahia | Brazil |  |  |  | X |  |  |  |  |  |
| *E. russatus* | UFRJ9338 |  | Banco Da Vitória | Ilhéus | Bahia | Brazil |  |  |  | X |  |  |  |  |  |
| *E. russatus* | MNRJ9339 |  | Banco Da Vitória | Ilhéus | Bahia | Brazil |  |  |  | X |  |  |  |  |  |
| *E. russatus* | MNRJ9343 |  | Banco Da Vitória | Ilhéus | Bahia | Brazil |  |  |  | X |  |  |  |  |  |
| *E. russatus* | UFRJ9347 |  | Banco Da Vitória | Ilhéus | Bahia | Brazil |  |  |  | X |  |  |  |  |  |
| *E. russatus* | UFRJ9350 |  | Banco Da Vitória | Ilhéus | Bahia | Brazil |  |  |  | X |  |  |  |  |  |
| *E. russatus* | MNRJ9353 |  | Banco Da Vitória | Ilhéus | Bahia | Brazil |  |  |  | X |  |  |  |  |  |
| *E. russatus* | MNRJ9355 |  | Banco Da Vitória | Ilhéus | Bahia | Brazil |  |  |  | X |  |  |  |  |  |
| *E. russatus* | MNRJ9356 |  | Banco Da Vitória | Ilhéus | Bahia | Brazil |  |  |  | X |  |  |  |  |  |
| *E. russatus* | MNRJ9358 |  | Banco Da Vitória | Ilhéus | Bahia | Brazil |  |  |  | X |  |  |  |  |  |
| *E. russatus* | MNRJ9360 |  | Banco Da Vitória | Ilhéus | Bahia | Brazil |  |  |  | X |  |  |  |  |  |
| *E. russatus* | MNRJ9381 |  | Banco Da Vitória | Ilhéus | Bahia | Brazil |  |  |  | X |  |  |  |  |  |
| *E. russatus* | MNRJ9382 |  | Banco Da Vitória | Ilhéus | Bahia | Brazil |  |  |  | X |  |  |  |  |  |
| *E. russatus* | MNRJ9385 |  | Banco Da Vitória | Ilhéus | Bahia | Brazil |  |  |  | X |  |  |  |  |  |
| *E. russatus* | MNRJ9389 |  | Banco Da Vitória | Ilhéus | Bahia | Brazil |  |  |  | X |  |  |  |  |  |
| *E. russatus* | MNRJ9397 |  | Banco Da Vitória | Ilhéus | Bahia | Brazil |  |  |  | X |  |  |  |  |  |
| *E. russatus* | MNRJ9404 |  | Banco Da Vitória | Ilhéus | Bahia | Brazil |  |  |  | X |  |  |  |  |  |
| *E. russatus* | UFRJ9406 |  | Banco Da Vitória | Ilhéus | Bahia | Brazil |  |  |  | X |  |  |  |  |  |
| *E. russatus* | UFRJ9432 |  | Banco Da Vitória | Ilhéus | Bahia | Brazil |  |  |  | X |  |  |  |  |  |
| *E. russatus* | MNRJ9434 |  | Banco Da Vitória | Ilhéus | Bahia | Brazil |  |  |  | X |  |  |  |  |  |
| *E. russatus* | MNRJ9442 |  | Buerarema | Ilhéus | Bahia | Brazil |  |  |  | X |  |  |  |  |  |
| *E. russatus* | MNRJ9443 |  | Buerarema | Ilhéus | Bahia | Brazil |  |  |  | X |  |  |  |  |  |
| *E. russatus* | MNRJ9445 |  | Buerarema | Ilhéus | Bahia | Brazil |  |  |  | X |  |  |  |  |  |
| *E. russatus* | MNRJ9446 |  | Buerarema | Ilhéus | Bahia | Brazil |  |  |  | X |  |  |  |  |  |
| *E. russatus* | MNRJ9447 |  | Buerarema | Ilhéus | Bahia | Brazil |  |  |  | X |  |  |  |  |  |
| *E. russatus* | MNRJ9454 |  | Buerarema | Ilhéus | Bahia | Brazil |  |  |  | X |  |  |  |  |  |
| *E. russatus* | MNRJ9463 |  | Buerarema | Ilhéus | Bahia | Brazil |  |  |  | X |  |  |  |  |  |
| *E. russatus* | MNRJ9467 |  | Buerarema | Ilhéus | Bahia | Brazil |  |  |  | X |  |  |  |  |  |
| *E. russatus* | MNRJ9478 |  | Buerarema | Ilhéus | Bahia | Brazil |  |  |  | X |  |  |  |  |  |
| *E. russatus* | MNRJ9490 |  | Buerarema | Ilhéus | Bahia | Brazil |  |  |  | X |  |  |  |  |  |
| *E. russatus* | UFRJ9497 |  | Buerarema | Ilhéus | Bahia | Brazil |  |  |  | X |  |  |  |  |  |
| *E. russatus* | MNRJ9499 |  | Buerarema | Ilhéus | Bahia | Brazil |  |  |  | X |  |  |  |  |  |
| *E. russatus* | MNRJ9500 |  | Buerarema | Ilhéus | Bahia | Brazil |  |  |  | X |  |  |  |  |  |
| *E. russatus* | MNRJ9512 |  | Buerarema | Ilhéus | Bahia | Brazil |  |  |  | X |  |  |  |  |  |
| *E. russatus* | MNRJ9514 |  | Buerarema | Ilhéus | Bahia | Brazil |  |  |  | X |  |  |  |  |  |
| *E. russatus* | MNRJ9516 |  | Buerarema | Ilhéus | Bahia | Brazil |  |  |  | X |  |  |  |  |  |
| *E. russatus* | MNRJ9520 |  | Burarema | Ilhéus | Bahia | Brazil |  |  |  | X |  |  |  |  |  |
| *E. russatus* | MNRJ-M10511 |  | Buerarema | Ilhéus | Bahia | Brazil |  |  |  | X |  |  |  |  |  |
| *E. russatus* | MNRJ9521 |  | Rio Do Braço | Ilhéus | Bahia | Brazil |  |  |  | X |  |  |  |  |  |
| *E. russatus* | MNRJ9525 |  | Rio Do Braço | Ilhéus | Bahia | Brazil |  |  |  | X |  |  |  |  |  |
| *E. russatus* | MNRJ9528 |  | Rio Do Braço | Ilhéus | Bahia | Brazil |  |  |  | X |  |  |  |  |  |
| *E. russatus* | MNRJ9530 |  | Rio Do Braço | Ilhéus | Bahia | Brazil |  |  |  | X |  |  |  |  |  |
| *E. russatus* | MNRJ9533 |  | Rio Do Braço | Ilhéus | Bahia | Brazil |  |  |  | X |  |  |  |  |  |
| *E. russatus* | MNRJ9538 |  | Rio Do Braço | Ilhéus | Bahia | Brazil |  |  |  | X |  |  |  |  |  |
| *E. russatus* | MNRJ9539 |  | Rio Do Braço | Ilhéus | Bahia | Brazil |  |  |  | X |  |  |  |  |  |
| *E. russatus* | MNRJ9550 |  | Rio Do Braço | Ilhéus | Bahia | Brazil |  |  |  | X |  |  |  |  |  |
| *E. russatus* | MNRJ9557 |  | Rio Do Braço | Ilhéus | Bahia | Brazil |  |  |  | X |  |  |  |  |  |
| *E. russatus* | MNRJ9567 |  | Rio Do Braço | Ilhéus | Bahia | Brazil |  |  |  | X |  |  |  |  |  |
| *E. russatus* | UFRJ9572 |  | Rio Do Braço | Ilhéus | Bahia | Brazil |  |  |  | X |  |  |  |  |  |
| *E. russatus* | MNRJ9574 |  | Rio Do Braço | Ilhéus | Bahia | Brazil |  |  |  | X |  |  |  |  |  |
| *E. russatus* | UFRJ9579 |  | Rio Do Braço | Ilhéus | Bahia | Brazil |  |  |  | X |  |  |  |  |  |
| *E. russatus* | UFRJ9582 |  | Rio Do Braço | Ilhéus | Bahia | Brazil |  |  |  | X |  |  |  |  |  |
| *E. russatus* | UFRJ9587 |  | Rio Do Braço | Ilhéus | Bahia | Brazil |  |  |  | X |  |  |  |  |  |
| *E. russatus* | MNRJ9591 |  | Rio Do Braço | Ilhéus | Bahia | Brazil |  |  |  | X |  |  |  |  |  |
| *E. russatus* | MNRJ9600 |  | Rio Do Braço | Ilhéus | Bahia | Brazil |  |  |  | X |  |  |  |  |  |
| *E. russatus* | MNRJ9622 |  | Rio Do Braço | Ilhéus | Bahia | Brazil |  |  |  | X |  |  |  |  |  |
| *E. russatus* | MNRJ9625 |  | Rio Do Braço | Ilhéus | Bahia | Brazil |  |  |  | X |  |  |  |  |  |
| *E. russatus* | MNRJ9641 |  | Rio Do Braço | Ilhéus | Bahia | Brazil |  |  |  | X |  |  |  |  |  |
| *E. russatus* | MNRJ9650 |  | Rio Do Braço | Ilhéus | Bahia | Brazil |  |  |  | X |  |  |  |  |  |
| *E. russatus* | MNRJ9659 |  | Rio Do Braço | Ilhéus | Bahia | Brazil |  |  |  | X |  |  |  |  |  |
| *E. russatus* | MNRJ9669 |  | Rio Do Braço | Ilhéus | Bahia | Brazil |  |  |  | X |  |  |  |  |  |
| *E. russatus* | MNRJ9671 |  | Escan | Una | Bahia | Brazil |  |  |  | X |  |  |  |  |  |
| *E. russatus* | MNRJ9676 |  | Fazenda Unacau, 8 Km Se De São José | Una | Bahia | Brazil |  |  |  | X |  |  |  |  |  |
| *E. russatus* | UFPB431 |  | Fazenda Unacau, 8 Km Se De São José | Una | Bahia | Brazil |  |  |  | X |  |  |  |  |  |
| *E. russatus* | UFPB432 |  | Fazenda Unacau, 8 Km Se De São José | Una | Bahia | Brazil |  |  |  | X |  |  |  |  |  |
| *E. russatus* | UFPB433 |  | Fazenda Unacau, 8 Km Se De São José | Una | Bahia | Brazil |  |  |  | X |  |  |  |  |  |
| *E. russatus* | UFPB434 |  | Fazenda Aldeia | Valença | Bahia | Brazil |  |  |  | X |  |  |  |  |  |
| *E. russatus* | UFPB385 |  | Campinho |  | Espírito Santo | Brazil |  |  |  | X |  |  |  |  |  |
| *E. russatus* | MBML#196 |  | Grota, 12 Km E Aracruz | Aracruz | Espírito Santo | Brazil |  |  |  | X |  |  |  |  |  |
| *E. russatus* | UFMG-LC27 |  | 4 Km N Castelinho | Cachoeiro Do Itapemirim | Espírito Santo | Brazil |  |  |  | X |  |  |  |  |  |
| *E. russatus* | UFMG-LC30 |  | R. B. Duas Bocas | Cariacica | Espírito Santo | Brazil |  |  |  | X |  |  |  |  |  |
| *E. russatus* | UFMG-LC31 |  | R. B. Duas Bocas | Cariacica | Espírito Santo | Brazil |  |  |  | X |  |  |  |  |  |
| *E. russatus* | MBML01 |  | R. B. Duas Bocas | Cariacica | Espírito Santo | Brazil |  |  |  | X |  |  |  |  |  |
| *E. russatus* | MBML02 |  | R. B. Duas Bocas | Cariacica | Espírito Santo | Brazil |  |  |  | X |  |  |  |  |  |
| *E. russatus* | MBML05 |  | R. B. Duas Bocas | Cariacica | Espírito Santo | Brazil |  |  |  | X |  |  |  |  |  |
| *E. russatus* | MBML06 |  | R. B. Duas Bocas | Cariacica | Espírito Santo | Brazil |  |  |  | X |  |  |  |  |  |
| *E. russatus* | MBML07 |  | Forno Grande, Faz. Barnabé | Castelo | Espírito Santo | Brazil |  |  |  | X |  |  |  |  |  |
| *E. russatus* | MBML08 |  | Forno Grande, 3Km Ne | Castelo | Espírito Santo | Brazil |  |  |  | X |  |  |  |  |  |
| *E. russatus* | MBML105 |  | Forno Grande, 3Km Ne | Castelo | Espírito Santo | Brazil |  |  |  | X |  |  |  |  |  |
| *E. russatus* | MBML12 |  | Fazenda Santa Terezinha, 33 Km Ne Linhares | Linhares | Espírito Santo | Brazil |  |  |  | X |  |  |  |  |  |
| *E. russatus* | MBML13 |  | Fazenda Santa Terezinha, 33 Km Ne Linhares | Linhares | Espírito Santo | Brazil |  |  |  | X |  |  |  |  |  |
| *E. russatus* | MBML138 |  | Fazenda Santa Terezinha, 33 Km Ne Linhares | Linhares | Espírito Santo | Brazil |  |  |  | X |  |  |  |  |  |
| *E. russatus* | MBML142 |  | Est. Biol. De Santa Lúcia | Santa Teresa | Espírito Santo | Brazil |  |  |  | X |  |  |  |  |  |
| *E. russatus* | MBML15 |  | Caixa D'Agua | Santa Teresa | Espírito Santo | Brazil |  |  |  | X |  |  |  |  |  |
| *E. russatus* | MBML16 |  | Caixa D'Agua | Santa Teresa | Espírito Santo | Brazil |  |  |  | X |  |  |  |  |  |
| *E. russatus* | MBML165 |  | Est. Biol. De Santa Lúcia | Santa Teresa | Espírito Santo | Brazil |  |  |  | X |  |  |  |  |  |
| *E. russatus* | MBML179 |  | Est. Biol. De Santa Lúcia | Santa Teresa | Espírito Santo | Brazil |  |  |  | X |  |  |  |  |  |
| *E. russatus* | MBML192 |  | Caixa D'Agua | Santa Teresa | Espírito Santo | Brazil |  |  |  | X |  |  |  |  |  |
| *E. russatus* | MBML318 |  | R. F. Nova Lombardia | Santa Teresa | Espírito Santo | Brazil |  |  |  | X |  |  |  |  |  |
| *E. russatus* | MBML329 |  | Caixa D'Agua | Santa Teresa | Espírito Santo | Brazil |  |  |  | X |  |  |  |  |  |
| *E. russatus* | MBML386 |  | Caixa D'Agua | Santa Teresa | Espírito Santo | Brazil |  |  |  | X |  |  |  |  |  |
| *E. russatus* | MBML396 |  | Est. Biol. De Santa Lúcia | Santa Teresa | Espírito Santo | Brazil |  |  |  | X |  |  |  |  |  |
| *E. russatus* | MBML50 |  | Caixa D'Agua | Santa Teresa | Espírito Santo | Brazil |  |  |  | X |  |  |  |  |  |
| *E. russatus* | MBML51 |  | Caixa D'Agua | Santa Teresa | Espírito Santo | Brazil |  |  |  | X |  |  |  |  |  |
| *E. russatus* | MBML88 |  | Est. Biol. De Santa Lúcia | Santa Teresa | Espírito Santo | Brazil |  |  |  | X |  |  |  |  |  |
| *E. russatus* | MBML91 |  | Est. Biol. De Santa Lúcia | Santa Teresa | Espírito Santo | Brazil |  |  |  | X |  |  |  |  |  |
| *E. russatus* | UFMG-MF29 |  | Est. Biol. De Santa Lúcia | Santa Teresa | Espírito Santo | Brazil |  |  |  | X |  |  |  |  |  |
| *E. russatus* | MNRJ24537 |  | Est. Biol. De Santa Lúcia | Santa Teresa | Espírito Santo | Brazil |  |  |  | X |  |  |  |  |  |
| *E. russatus* | MNRJ24538 |  | Est. Biol. De Santa Lúcia | Santa Teresa | Espírito Santo | Brazil |  |  |  | X |  |  |  |  |  |
| *E. russatus* | MNRJ24539 |  | Caixa D'Agua | Santa Teresa | Espírito Santo | Brazil |  |  |  | X |  |  |  |  |  |
| *E. russatus* | MNRJ24540 |  | Caixa D'Agua | Santa Teresa | Espírito Santo | Brazil |  |  |  | X |  |  |  |  |  |
| *E. russatus* | MNRJ24541 |  | Caixa D'Agua | Santa Teresa | Espírito Santo | Brazil |  |  |  | X |  |  |  |  |  |
| *E. russatus* | MNRJ31410 |  | Caixa D'Agua | Santa Teresa | Espírito Santo | Brazil |  |  |  | X |  |  |  |  |  |
| *E. russatus* | MNRJ31411 |  | Est. Biol. De Santa Lúcia | Santa Teresa | Espírito Santo | Brazil |  |  |  | X |  |  |  |  |  |
| *E. russatus* | MNRJ32674 |  | Est. Biol. De Santa Lúcia | Santa Teresa | Espírito Santo | Brazil |  |  |  | X |  |  |  |  |  |
| *E. russatus* | MNRJ32675 |  | R.F. Nova Lombardia | Santa Teresa | Espírito Santo | Brazil |  |  |  | X |  |  |  |  |  |
| *E. russatus* | MNRJ32678 |  | R.F. Nova Lombardia | Santa Teresa | Espírito Santo | Brazil |  |  |  | X |  |  |  |  |  |
| *E. russatus* | MNRJ32765 |  | R.F. Nova Lombardia | Santa Teresa | Espírito Santo | Brazil |  |  |  | X |  |  |  |  |  |
| *E. russatus* | MNRJ32766 |  | R.F. Nova Lombardia | Santa Teresa | Espírito Santo | Brazil |  |  |  | X |  |  |  |  |  |
| *E. russatus* | MNRJ32767 |  | R.F. Nova Lombardia | Santa Teresa | Espírito Santo | Brazil |  |  |  | X |  |  |  |  |  |
| *E. russatus* | MNRJ32768 |  | R.F. Nova Lombardia | Santa Teresa | Espírito Santo | Brazil |  |  |  | X |  |  |  |  |  |
| *E. russatus* | MNRJ32769 |  | R.F. Nova Lombardia | Santa Teresa | Espírito Santo | Brazil |  |  |  | X |  |  |  |  |  |
| *E. russatus* | MNRJ32770 |  | R.F. Nova Lombardia | Santa Teresa | Espírito Santo | Brazil |  |  |  | X |  |  |  |  |  |
| *E. russatus* | MNRJ32771 |  | R.F. Nova Lombardia | Santa Teresa | Espírito Santo | Brazil |  |  |  | X |  |  |  |  |  |
| *E. russatus* | MNRJ32772 |  | R.F. Nova Lombardia | Santa Teresa | Espírito Santo | Brazil |  |  |  | X |  |  |  |  |  |
| *E. russatus* | MNRJ32773 |  | R.F. Nova Lombardia | Santa Teresa | Espírito Santo | Brazil |  |  |  | X |  |  |  |  |  |
| *E. russatus* | MNRJ32774 |  | R.F. Nova Lombardia | Santa Teresa | Espírito Santo | Brazil |  |  |  | X |  |  |  |  |  |
| *E. russatus* | MNRJ32775 |  | R.F. Nova Lombardia | Santa Teresa | Espírito Santo | Brazil |  |  |  | X |  |  |  |  |  |
| *E. russatus* | MNRJ32776 |  | R.F. Nova Lombardia | Santa Teresa | Espírito Santo | Brazil |  |  |  | X |  |  |  |  |  |
| *E. russatus* | MNRJ32777 |  | R.F. Nova Lombardia | Santa Teresa | Espírito Santo | Brazil |  |  |  | X |  |  |  |  |  |
| *E. russatus* | MNRJ32778 |  | R.F. Nova Lombardia | Santa Teresa | Espírito Santo | Brazil |  |  |  | X |  |  |  |  |  |
| *E. russatus* | MNRJ32779 |  | R.F. Nova Lombardia | Santa Teresa | Espírito Santo | Brazil |  |  |  | X |  |  |  |  |  |
| *E. russatus* | MNRJ32780 |  | R.F. Nova Lombardia | Santa Teresa | Espírito Santo | Brazil |  |  |  | X |  |  |  |  |  |
| *E. russatus* | MNRJ32781 |  | R.F. Nova Lombardia | Santa Teresa | Espírito Santo | Brazil |  |  |  | X |  |  |  |  |  |
| *E. russatus* | MNRJ32782 |  | R.F. Nova Lombardia | Santa Teresa | Espírito Santo | Brazil |  |  |  | X |  |  |  |  |  |
| *E. russatus* | MNRJ32783 |  | R.F. Nova Lombardia | Santa Teresa | Espírito Santo | Brazil |  |  |  | X |  |  |  |  |  |
| *E. russatus* | MNRJ32785 |  | R.F. Nova Lombardia | Santa Teresa | Espírito Santo | Brazil |  |  |  | X |  |  |  |  |  |
| *E. russatus* | MNRJ32786 |  | R.F. Nova Lombardia | Santa Teresa | Espírito Santo | Brazil |  |  |  | X |  |  |  |  |  |
| *E. russatus* | MNRJ32787 |  | R.F. Nova Lombardia | Santa Teresa | Espírito Santo | Brazil |  |  |  | X |  |  |  |  |  |
| *E. russatus* | MNRJ32788 |  | R.F. Nova Lombardia | Santa Teresa | Espírito Santo | Brazil |  |  |  | X |  |  |  |  |  |
| *E. russatus* | MNRJ32789 |  | R.F. Nova Lombardia | Santa Teresa | Espírito Santo | Brazil |  |  |  | X |  |  |  |  |  |
| *E. russatus* | MNRJ32790 |  | R.F. Nova Lombardia | Santa Teresa | Espírito Santo | Brazil |  |  |  | X |  |  |  |  |  |
| *E. russatus* | MNRJ32791 |  | R.F. Nova Lombardia | Santa Teresa | Espírito Santo | Brazil |  |  |  | X |  |  |  |  |  |
| *E. russatus* | MNRJ32792 |  | R.F. Nova Lombardia | Santa Teresa | Espírito Santo | Brazil |  |  |  | X |  |  |  |  |  |
| *E. russatus* | MNRJ32793 |  | R.F. Nova Lombardia | Santa Teresa | Espírito Santo | Brazil |  |  |  | X |  |  |  |  |  |
| *E. russatus* | MNRJ32794 |  | R.F. Nova Lombardia | Santa Teresa | Espírito Santo | Brazil |  |  |  | X |  |  |  |  |  |
| *E. russatus* | MNRJ32795 |  | R.F. Nova Lombardia | Santa Teresa | Espírito Santo | Brazil |  |  |  | X |  |  |  |  |  |
| *E. russatus* | MNRJ32796 |  | R.F. Nova Lombardia | Santa Teresa | Espírito Santo | Brazil |  |  |  | X |  |  |  |  |  |
| *E. russatus* | MNRJ32797 |  | R.F. Nova Lombardia | Santa Teresa | Espírito Santo | Brazil |  |  |  | X |  |  |  |  |  |
| *E. russatus* | MNRJ32798 |  | R.F. Nova Lombardia | Santa Teresa | Espírito Santo | Brazil |  |  |  | X |  |  |  |  |  |
| *E. russatus* | MNRJ32799 |  | R.F. Nova Lombardia | Santa Teresa | Espírito Santo | Brazil |  |  |  | X |  |  |  |  |  |
| *E. russatus* | MNRJ32800 |  | R.F. Nova Lombardia | Santa Teresa | Espírito Santo | Brazil |  |  |  | X |  |  |  |  |  |
| *E. russatus* | MNRJ32801 |  | R.F. Nova Lombardia | Santa Teresa | Espírito Santo | Brazil |  |  |  | X |  |  |  |  |  |
| *E. russatus* | MNRJ32802 |  | R.F. Nova Lombardia | Santa Teresa | Espírito Santo | Brazil |  |  |  | X |  |  |  |  |  |
| *E. russatus* | MNRJ32803 |  | R.F. Nova Lombardia | Santa Teresa | Espírito Santo | Brazil |  |  |  | X |  |  |  |  |  |
| *E. russatus* | MNRJ32804 |  | Caixa D'Agua, Site A | Santa Teresa | Espírito Santo | Brazil |  |  |  | X |  |  |  |  |  |
| *E. russatus* | MNRJ32805 |  | Caixa D'Agua, Site B | Santa Teresa | Espírito Santo | Brazil |  |  |  | X |  |  |  |  |  |
| *E. russatus* | MNRJ32806 |  | Caixa D'Agua, Site B | Santa Teresa | Espírito Santo | Brazil |  |  |  | X |  |  |  |  |  |
| *E. russatus* | MNRJ32807 |  | Caixa D'Agua, Site A | Santa Teresa | Espírito Santo | Brazil |  |  |  | X |  |  |  |  |  |
| *E. russatus* | MNRJ32808 |  | Caixa D'Agua, Site A | Santa Teresa | Espírito Santo | Brazil |  |  |  | X |  |  |  |  |  |
| *E. russatus* | MNRJ32809 |  | R.F. Goipapoçu | Santa Teresa | Espírito Santo | Brazil |  |  |  | X |  |  |  |  |  |
| *E. russatus* | MNRJ32810 |  | Caixa D'Agua, Site A | Santa Teresa | Espírito Santo | Brazil |  |  |  | X |  |  |  |  |  |
| *E. russatus* | MNRJ32811 |  | Caixa D'Agua | Santa Teresa | Espírito Santo | Brazil |  |  |  | X |  |  |  |  |  |
| *E. russatus* | MNRJ32812 |  | R.F. Goipapoçu | Santa Teresa | Espírito Santo | Brazil |  |  |  | X |  |  |  |  |  |
| *E. russatus* | MNRJ32813 |  | Caixa D'Agua | Santa Teresa | Espírito Santo | Brazil |  |  |  | X |  |  |  |  |  |
| *E. russatus* | MNRJ32814 |  | Caixa D'Agua, Site B | Santa Teresa | Espírito Santo | Brazil |  |  |  | X |  |  |  |  |  |
| *E. russatus* | MNRJ32815 |  | R.F. Goipapoçu | Santa Teresa | Espírito Santo | Brazil |  |  |  | X |  |  |  |  |  |
| *E. russatus* | MNRJ32816 |  | R.F. Goipapoçu | Santa Teresa | Espírito Santo | Brazil |  |  |  | X |  |  |  |  |  |
| *E. russatus* | MNRJ32817 |  | R.F. Goipapoçu | Santa Teresa | Espírito Santo | Brazil |  |  |  | X |  |  |  |  |  |
| *E. russatus* | MNRJ32818 |  | R.F. Goipapoçu | Santa Teresa | Espírito Santo | Brazil |  |  |  | X |  |  |  |  |  |
| *E. russatus* | MNRJ32819 |  | R.F. Nova Lombardia | Santa Teresa | Espírito Santo | Brazil |  |  |  | X |  |  |  |  |  |
| *E. russatus* | MNRJ32820 |  | Caixa D'Agua, Site A | Santa Teresa | Espírito Santo | Brazil |  |  |  | X |  |  |  |  |  |
| *E. russatus* | MNRJ32821 |  | R.F. Nova Lombardia | Santa Teresa | Espírito Santo | Brazil |  |  |  | X |  |  |  |  |  |
| *E. russatus* | MNRJ32822 |  | R.F. Nova Lombardia | Santa Teresa | Espírito Santo | Brazil |  |  |  | X |  |  |  |  |  |
| *E. russatus* | MNRJ32824 |  | Caixa D'Agua, Site B | Santa Teresa | Espírito Santo | Brazil |  |  |  | X |  |  |  |  |  |
| *E. russatus* | MNRJ32825 |  | Caixa D'Agua | Santa Teresa | Espírito Santo | Brazil |  |  |  | X |  |  |  |  |  |
| *E. russatus* | MNRJ32826 |  | Caixa D'Agua | Santa Teresa | Espírito Santo | Brazil |  |  |  | X |  |  |  |  |  |
| *E. russatus* | MNRJ32827 |  | R.F. Nova Lombardia | Santa Teresa | Espírito Santo | Brazil |  |  |  | X |  |  |  |  |  |
| *E. russatus* | MNRJ32828 |  | R.F. Nova Lombardia | Santa Teresa | Espírito Santo | Brazil |  |  |  | X |  |  |  |  |  |
| *E. russatus* | MNRJ32829 |  | Caixa D'Agua | Santa Teresa | Espírito Santo | Brazil |  |  |  | X |  |  |  |  |  |
| *E. russatus* | MNRJ5320 |  | Caixa D'Agua | Santa Teresa | Espírito Santo | Brazil |  |  |  | X |  |  |  |  |  |
| *E. russatus* | MNRJ5323 |  | Caixa D'Agua | Santa Teresa | Espírito Santo | Brazil |  |  |  | X |  |  |  |  |  |
| *E. russatus* | MNRJ7091 |  | Caixa D'Agua | Santa Teresa | Espírito Santo | Brazil |  |  |  | X |  |  |  |  |  |
| *E. russatus* | MNRJ8250 |  | Caixa D'Agua | Santa Tersa | Espírito Santo | Brazil |  |  |  | X |  |  |  |  |  |
| *E. russatus* | MNRJ8291 |  | Hotel Fazenda Monte Verde, 24 Km Se De Venda Nova | Venda Nova ? | Espírito Santo | Brazil |  |  |  | X |  |  |  |  |  |
| *E. russatus* | UFPB368 |  | Hotel Fazenda Monte Verde, 24 Km Se De Venda Nova | Venda Nova ? | Espírito Santo | Brazil |  |  |  | X |  |  |  |  |  |
| *E. russatus* | UFPB369 |  | Hotel Fazenda Monte Verde, 24 Km Se De Venda Nova | Venda Nova ? | Espírito Santo | Brazil |  |  |  | X |  |  |  |  |  |
| *E. russatus* | UFPB370 |  | Hotel Fazenda Monte Verde, 24 Km Se De Venda Nova | Venda Nova ? | Espírito Santo | Brazil |  |  |  | X |  |  |  |  |  |
| *E. russatus* | UFPB371 |  | Hotel Fazenda Monte Verde, 24 Km Se De Venda Nova | Venda Nova ? | Espírito Santo | Brazil |  |  |  | X |  |  |  |  |  |
| *E. russatus* | UFPB373 |  | Hotel Fazenda Monte Verde, 24 Km Se De Venda Nova | Venda Nova ? | Espírito Santo | Brazil |  |  |  | X |  |  |  |  |  |
| *E. russatus* | UFPB374 |  | Hotel Fazenda Monte Verde, 24 Km Se De Venda Nova | Venda Nova ? | Espírito Santo | Brazil |  |  |  | X |  |  |  |  |  |
| *E. russatus* | UFPB375 |  | Hotel Fazenda Monte Verde, 24 Km Se De Venda Nova | Venda Nova ? | Espírito Santo | Brazil |  |  |  | X |  |  |  |  |  |
| *E. russatus* | UFPB376 |  | Hotel Fazenda Monte Verde, 24 Km Se De Venda Nova | Venda Nova ? | Espírito Santo | Brazil |  |  |  | X |  |  |  |  |  |
| *E. russatus* | UFPB377 |  | Hotel Fazenda Monte Verde, 24 Km Se De Venda Nova | Venda Nova ? | Espírito Santo | Brazil |  |  |  | X |  |  |  |  |  |
| *E. russatus* | AMNH61835 |  | Fazenda Paraiso | Alem Paraiba | Minas Gerais | Brazil |  |  |  | X |  |  |  |  |  |
| *E. russatus* | AMNH61837 |  | Fazenda Paraiso | Alem Paraiba | Minas Gerais | Brazil |  |  |  | X |  |  |  |  |  |
| *E. russatus* | CEM3719 |  | Fazenda Sao Geraldo | Alem Paraiba | Minas Gerais | Brazil |  |  |  | X |  |  |  |  |  |
| *E. russatus* | MNRJ11231 |  | Fazenda Paraiso | Alem Paraiba | Minas Gerais | Brazil |  |  |  | X |  |  |  |  |  |
| *E. russatus* | MNRJ11647 |  | Fazenda Sao Geraldo | Alem Paraiba | Minas Gerais | Brazil |  |  |  | X |  |  |  |  |  |
| *E. russatus* | MNRJ11725 |  | Fazenda Sao Geraldo | Alem Paraiba | Minas Gerais | Brazil |  |  |  | X |  |  |  |  |  |
| *E. russatus* | MNRJ11726 |  | Fazenda Paraiso | Alem Paraiba | Minas Gerais | Brazil |  |  |  | X |  |  |  |  |  |
| *E. russatus* | MNRJ11727 |  | Fazenda Sao Geraldo | Alem Paraiba | Minas Gerais | Brazil |  |  |  | X |  |  |  |  |  |
| *E. russatus* | MNRJ11729 |  | Fazenda Sao Geraldo | Alem Paraiba | Minas Gerais | Brazil |  |  |  | X |  |  |  |  |  |
| *E. russatus* | MNRJ11911 |  | Fazenda Sao Geraldo | Alem Paraiba | Minas Gerais | Brazil |  |  |  | X |  |  |  |  |  |
| *E. russatus* | MNRJ32751 |  | Fazenda Sao Geraldo | Alem Paraiba | Minas Gerais | Brazil |  |  |  | X |  |  |  |  |  |
| *E. russatus* | MNRJ32752 |  | Fazenda Sao Geraldo | Alem Paraiba | Minas Gerais | Brazil |  |  |  | X |  |  |  |  |  |
| *E. russatus* | MNRJ32753 |  | Fazenda Sao Geraldo | Alem Paraiba | Minas Gerais | Brazil |  |  |  | X |  |  |  |  |  |
| *E. russatus* | MNRJ32754 |  | Fazenda Paraiso | Alem Paraiba | Minas Gerais | Brazil |  |  |  | X |  |  |  |  |  |
| *E. russatus* | MNRJ32755 |  | Fazenda Sao Geraldo | Alem Paraiba | Minas Gerais | Brazil |  |  |  | X |  |  |  |  |  |
| *E. russatus* | MNRJ32756 |  | Fazenda Sao Geraldo | Alem Paraiba | Minas Gerais | Brazil |  |  |  | X |  |  |  |  |  |
| *E. russatus* | MNRJ32757 |  | Fazenda Paraiso | Alem Paraiba | Minas Gerais | Brazil |  |  |  | X |  |  |  |  |  |
| *E. russatus* | MNRJ32758 |  | Fazenda Sao Geraldo | Alem Paraiba | Minas Gerais | Brazil |  |  |  | X |  |  |  |  |  |
| *E. russatus* | MNRJ32759 |  | Fazenda Sao Geraldo | Alem Paraiba | Minas Gerais | Brazil |  |  |  | X |  |  |  |  |  |
| *E. russatus* | MNRJ32760 |  | Fazenda Sao Geraldo | Alem Paraiba | Minas Gerais | Brazil |  |  |  | X |  |  |  |  |  |
| *E. russatus* | MNRJ32761 |  | Fazenda Sao Geraldo | Alem Paraiba | Minas Gerais | Brazil |  |  |  | X |  |  |  |  |  |
| *E. russatus* | MNRJ32762 |  | Fazenda Sao Geraldo | Alem Paraiba | Minas Gerais | Brazil |  |  |  | X |  |  |  |  |  |
| *E. russatus* | MNRJ32763 |  | Fazenda Sao Geraldo | Alem Paraiba | Minas Gerais | Brazil |  |  |  | X |  |  |  |  |  |
| *E. russatus* | MNRJ5260 |  | Fazenda Sao Geraldo | Alem Paraiba | Minas Gerais | Brazil |  |  |  | X |  |  |  |  |  |
| *E. russatus* | MNRJ5340 |  | Fazenda Sao Geraldo | Alem Paraiba | Minas Gerais | Brazil |  |  |  | X |  |  |  |  |  |
| *E. russatus* | MNRJ5380 |  | Fazenda Sao Geraldo | Alem Paraiba | Minas Gerais | Brazil |  |  |  | X |  |  |  |  |  |
| *E. russatus* | MNRJ7327 |  | Fazenda Sao Geraldo | Alem Paraiba | Minas Gerais | Brazil |  |  |  | X |  |  |  |  |  |
| *E. russatus* | MNRJ7329 |  | Fazenda Sao Geraldo | Alem Paraiba | Minas Gerais | Brazil |  |  |  | X |  |  |  |  |  |
| *E. russatus* | MNRJ7331 |  | Fazenda Sao Geraldo | Alem Paraiba | Minas Gerais | Brazil |  |  |  | X |  |  |  |  |  |
| *E. russatus* | MNRJ7333 |  | Fazenda Sao Geraldo | Alem Paraiba | Minas Gerais | Brazil |  |  |  | X |  |  |  |  |  |
| *E. russatus* | MNRJ7334 |  | Fazenda Sao Geraldo | Alem Paraiba | Minas Gerais | Brazil |  |  |  | X |  |  |  |  |  |
| *E. russatus* | MNRJ7341 |  | Fazenda Sao Geraldo | Alem Paraiba | Minas Gerais | Brazil |  |  |  | X |  |  |  |  |  |
| *E. russatus* | MNRJ7346 |  | Fazenda Sao Geraldo | Alem Paraiba | Minas Gerais | Brazil |  |  |  | X |  |  |  |  |  |
| *E. russatus* | MNRJ7350 |  | Fazenda Sao Geraldo | Alem Paraiba | Minas Gerais | Brazil |  |  |  | X |  |  |  |  |  |
| *E. russatus* | MNRJ7353 |  | Fazenda Sao Geraldo | Alem Paraiba | Minas Gerais | Brazil |  |  |  | X |  |  |  |  |  |
| *E. russatus* | MNRJ7355 |  | Fazenda Sao Geraldo | Alem Paraiba | Minas Gerais | Brazil |  |  |  | X |  |  |  |  |  |
| *E. russatus* | MNRJ7356 |  | Fazenda Paraiso | Alem Paraiba | Minas Gerais | Brazil |  |  |  | X |  |  |  |  |  |
| *E. russatus* | MNRJ7358 |  | Fazenda Sao Geraldo | Alem Paraiba | Minas Gerais | Brazil |  |  |  | X |  |  |  |  |  |
| *E. russatus* | MNRJ7361 |  | Fazenda Paraiso | Alem Paraiba | Minas Gerais | Brazil |  |  |  | X |  |  |  |  |  |
| *E. russatus* | MNRJ7366 |  | Fazenda Sao Geraldo | Alem Paraiba | Minas Gerais | Brazil |  |  |  | X |  |  |  |  |  |
| *E. russatus* | MNRJ7367 |  | Fazenda Sao Geraldo | Alem Paraiba | Minas Gerais | Brazil |  |  |  | X |  |  |  |  |  |
| *E. russatus* | MNRJ7371 |  | Fazenda Sao Geraldo | Alem Paraiba | Minas Gerais | Brazil |  |  |  | X |  |  |  |  |  |
| *E. russatus* | MNRJ7376 |  | Fazenda Sao Geraldo | Alem Paraiba | Minas Gerais | Brazil |  |  |  | X |  |  |  |  |  |
| *E. russatus* | MNRJ7378 |  | Fazenda Sao Geraldo | Alem Paraiba | Minas Gerais | Brazil |  |  |  | X |  |  |  |  |  |
| *E. russatus* | MNRJ7379 |  | Fazenda Sao Geraldo | Alem Paraiba | Minas Gerais | Brazil |  |  |  | X |  |  |  |  |  |
| *E. russatus* | MNRJ7380 |  | Fazenda Sao Geraldo | Alem Paraiba | Minas Gerais | Brazil |  |  |  | X |  |  |  |  |  |
| *E. russatus* | MNRJ7381 |  | Fazenda Sao Geraldo | Alem Paraiba | Minas Gerais | Brazil |  |  |  | X |  |  |  |  |  |
| *E. russatus* | MNRJ7382 |  | Fazenda Sao Geraldo | Alem Paraiba | Minas Gerais | Brazil |  |  |  | X |  |  |  |  |  |
| *E. russatus* | MNRJ7383 |  | Fazenda Sao Geraldo | Alem Paraiba | Minas Gerais | Brazil |  |  |  | X |  |  |  |  |  |
| *E. russatus* | MNRJ7385 |  | Fazenda Sao Geraldo | Alem Paraiba | Minas Gerais | Brazil |  |  |  | X |  |  |  |  |  |
| *E. russatus* | MNRJ7386 |  | Fazenda Sao Geraldo | Alem Paraiba | Minas Gerais | Brazil |  |  |  | X |  |  |  |  |  |
| *E. russatus* | MNRJ7387 |  | Fazenda Sao Geraldo | Alem Paraiba | Minas Gerais | Brazil |  |  |  | X |  |  |  |  |  |
| *E. russatus* | MNRJ7388 |  | Fazenda Sao Geraldo | Alem Paraiba | Minas Gerais | Brazil |  |  |  | X |  |  |  |  |  |
| *E. russatus* | MNRJ7414 |  | Fazenda Sao Geraldo | Alem Paraiba | Minas Gerais | Brazil |  |  |  | X |  |  |  |  |  |
| *E. russatus* | MNRJ7415 |  | Fazenda Sao Geraldo | Alem Paraiba | Minas Gerais | Brazil |  |  |  | X |  |  |  |  |  |
| *E. russatus* | MNRJ7422 |  | Fazenda São Geraldo | Alêm Paraiba | Minas Gerais | Brazil |  |  |  | X |  |  |  |  |  |
| *E. russatus* | MNRJ7424 |  | Fazenda São Geraldo | Alêm Paraíba | Minas Gerais | Brazil |  |  |  | X |  |  |  |  |  |
| *E. russatus* | MNRJ7425 |  | Fazenda São Geraldo | Alêm Paraíba | Minas Gerais | Brazil |  |  |  | X |  |  |  |  |  |
| *E. russatus* | MNRJ7427 |  | Fazenda São Geraldo | Alêm Paraíba | Minas Gerais | Brazil |  |  |  | X |  |  |  |  |  |
| *E. russatus* | MNRJ7429 |  | Fazenda São Geraldo | Alêm Paraíba | Minas Gerais | Brazil |  |  |  | X |  |  |  |  |  |
| *E. russatus* | MNRJ7430 |  | Fazenda São Geraldo | Alêm Paraíba | Minas Gerais | Brazil |  |  |  | X |  |  |  |  |  |
| *E. russatus* | MNRJ7436 |  | Fazenda São Geraldo | Alêm Paraíba | Minas Gerais | Brazil |  |  |  | X |  |  |  |  |  |
| *E. russatus* | MNRJ7441 |  | Fazenda São Geraldo | Alêm Paraíba | Minas Gerais | Brazil |  |  |  | X |  |  |  |  |  |
| *E. russatus* | MNRJ7443 |  | Fazenda São Geraldo | Alêm Paraíba | Minas Gerais | Brazil |  |  |  | X |  |  |  |  |  |
| *E. russatus* | MNRJ7447 |  | Fazenda São Geraldo | Alêm Paraíba | Minas Gerais | Brazil |  |  |  | X |  |  |  |  |  |
| *E. russatus* | MNRJ7449 |  | Caparaó, Faz. Cardoso | Caparaó | Minas Gerais | Brazil |  |  |  | X |  |  |  |  |  |
| *E. russatus* | MNRJ7470 |  | Caparaó, Faz. Cardoso | Caparaó | Minas Gerais | Brazil |  |  |  | X |  |  |  |  |  |
| *E. russatus* | MNRJ7492 |  | Passos | Passos | Minas Gerais | Brazil |  |  |  | X |  |  |  |  |  |
| *E. russatus* | MNRJ7493 |  | Passos | Passos | Minas Gerais | Brazil |  |  |  | X |  |  |  |  |  |
| *E. russatus* | MNRJ7494 |  | Passos | Passos | Minas Gerais | Brazil |  |  |  | X |  |  |  |  |  |
| *E. russatus* | MNRJ7495 |  | Passos | Passos | Minas Gerais | Brazil |  |  |  | X |  |  |  |  |  |
| *E. russatus* | MNRJ7496 |  | Passos | Passos | Minas Gerais | Brazil |  |  |  | X |  |  |  |  |  |
| *E. russatus* | MNRJ7498 |  | Passos | Passos | Minas Gerais | Brazil |  |  |  | X |  |  |  |  |  |
| *E. russatus* | MNRJ7499 |  | Passos | Passos | Minas Gerais | Brazil |  |  |  | X |  |  |  |  |  |
| *E. russatus* | MNRJ7501 |  | Passos | Passos | Minas Gerais | Brazil |  |  |  | X |  |  |  |  |  |
| *E. russatus* | MNRJ7503 |  | Passos | Passos | Minas Gerais | Brazil |  |  |  | X |  |  |  |  |  |
| *E. russatus* | MNRJ7506 |  | Passos | Passos | Minas Gerais | Brazil |  |  |  | X |  |  |  |  |  |
| *E. russatus* | MNRJ7507 |  | Passos | Passos | Minas Gerais | Brazil |  |  |  | X |  |  |  |  |  |
| *E. russatus* | MNRJ7513 |  | Passos | Passos | Minas Gerais | Brazil |  |  |  | X |  |  |  |  |  |
| *E. russatus* | MNRJ7535 |  | Passos | Passos | Minas Gerais | Brazil |  |  |  | X |  |  |  |  |  |
| *E. russatus* | MNRJ7544 |  | Passos | Passos | Minas Gerais | Brazil |  |  |  | X |  |  |  |  |  |
| *E. russatus* | MNRJ7549 |  | Passos | Passos | Minas Gerais | Brazil |  |  |  | X |  |  |  |  |  |
| *E. russatus* | MNRJ7550 |  | Passos | Passos | Minas Gerais | Brazil |  |  |  | X |  |  |  |  |  |
| *E. russatus* | MNRJ9424 |  | Passos | Passos | Minas Gerais | Brazil |  |  |  | X |  |  |  |  |  |
| *E. russatus* | MNRJ9971 |  | Passos | Passos | Minas Gerais | Brazil |  |  |  | X |  |  |  |  |  |
| *E. russatus* | MZUSP20571 |  | Passos | Passos | Minas Gerais | Brazil |  |  |  | X |  |  |  |  |  |
| *E. russatus* | UFPB-AL1412 |  | Mata Do Pau Ferro, 6 Km W De Areias | Areias ? |  | Brazil |  |  |  | X |  |  |  |  |  |
| *E. russatus* | MHNCI1311 |  | Uhe Parigot De Souza, Bairro Alto | Antonina |  | Brazil |  |  |  | X |  |  |  |  |  |
| ***E. russatus*** |  | **IIM076** | **Ortigueira,** | **Ortigueira,** |  | **Brazil** | **-24.13** | **-51.03** | **94** |  | **X** | **MT118066** | **X** | **MT118090** | **This study** |
| ***E. russatus*** |  | **IIM130** | **Ortigueira** | **Ortigueira** |  | **Brazil** | **-24.13** | **-51.03** | **94** |  | **X** | **MT118067** | **X** | **MT118091** | **This study** |
| *E. russatus* | MHNCI1313 |  | R.F. Guaraquçaba | Guaraqueçaba |  | Brazil |  |  |  | X |  |  |  |  |  |
| *E. russatus* | MHNCI1314 |  | R.F. Guaraquçaba, Laranjeiras (Ilha) | Guaraqueçaba |  | Brazil |  |  |  | X |  |  |  |  |  |
| *E. russatus* | MHNCI1319 |  | R.F. Guaraquçaba, Morro Do Tromomo | Guaraqueçaba |  | Brazil |  |  |  | X |  |  |  |  |  |
| *E. russatus* | MHNCI1320 |  | R.F. Guaraquçaba, Laranjeiras (Continente) | Guaraqueçaba |  | Brazil |  |  |  | X |  |  |  |  |  |
| *E. russatus* | MHNCI1324 |  | R.F. Guaraquçaba, Morro Do Tromomo | Guaraqueçaba |  | Brazil |  |  |  | X |  |  |  |  |  |
| *E. russatus* | MHNCI1326 |  | R.F. Guaraquçaba, Morro Do Tromomo | Guaraqueçaba |  | Brazil |  |  |  | X |  |  |  |  |  |
| *E. russatus* | MHNCI1328 |  | R.F. Guaraquçaba, Morro Do Tromomo | Guaraqueçaba |  | Brazil |  |  |  | X |  |  |  |  |  |
| *E. russatus* | MHNCI1331 |  | R.F. Guaraquçaba, Morro Do Tromomo | Guaraqueçaba |  | Brazil |  |  |  | X |  |  |  |  |  |
| *E. russatus* | MHNCI1332 |  | R.F. Guaraquçaba, Morro Do Tromomo | Guaraqueçaba |  | Brazil |  |  |  | X |  |  |  |  |  |
| *E. russatus* | MHNCI1334 |  | R.F. Guaraquçaba, Morro Do Tromomo | Guaraqueçaba |  | Brazil |  |  |  | X |  |  |  |  |  |
| *E. russatus* | MHNCI1337 |  | R.F. Guaraquçaba, Morro Do Tromomo | Guaraqueçaba |  | Brazil |  |  |  | X |  |  |  |  |  |
| *E. russatus* | MHNCI1340 |  | Reserva | Pinhão |  | Brazil |  |  |  | X |  |  |  |  |  |
| *E. russatus* | MHNCI1341 |  | Reserva | Pinhão |  | Brazil |  |  |  | X |  |  |  |  |  |
| *E. russatus* | MHNCI1342 |  | Represa De Guaricana | São José Do Pinhais |  | Brazil |  |  |  | X |  |  |  |  |  |
| *E. russatus* | MHNCI1344 |  | Represa De Guaricana | São José Do Pinhais |  | Brazil |  |  |  | X |  |  |  |  |  |
| *E. russatus* | MHNCI1348 |  | Represa De Guaricana | São José Do Pinhais |  | Brazil |  |  |  | X |  |  |  |  |  |
| *E. russatus* | MHNCI1351 |  | Represa De Guaricana | São José Do Pinhais |  | Brazil |  |  |  | X |  |  |  |  |  |
| *E. russatus* | MHNCI1355 |  | Represa De Guaricana | São José Do Pinhais |  | Brazil |  |  |  | X |  |  |  |  |  |
| *E. russatus* | MHNCI1356 |  | Represa De Guaricana | São José Do Pinhais |  | Brazil |  |  |  | X |  |  |  |  |  |
| *E. russatus* | MHNCI1359 |  | Represa De Guaricana | São José Do Pinhais |  | Brazil |  |  |  | X |  |  |  |  |  |
| *E. russatus* | MHNCI1361 |  | Represa De Guaricana | São José Do Pinhais |  | Brazil |  |  |  | X |  |  |  |  |  |
| *E. russatus* | MHNCI1376 |  | Represa De Guaricana | São José Do Pinhais |  | Brazil |  |  |  | X |  |  |  |  |  |
| *E. russatus* | MHNCI1379 |  | Represa De Guaricana | São José Do Pinhais |  | Brazil |  |  |  | X |  |  |  |  |  |
| *E. russatus* | MHNCI1380 |  | Represa De Guaricana | São José Do Pinhais |  | Brazil |  |  |  | X |  |  |  |  |  |
| *E. russatus* | MHNCI1381 |  | Represa De Guaricana | São José Do Pinhais |  | Brazil |  |  |  | X |  |  |  |  |  |
| *E. russatus* | MHNCI1387 |  | Represa De Guaricana | São José Do Pinhais |  | Brazil |  |  |  | X |  |  |  |  |  |
| *E. russatus* | MHNCI1388 |  | Represa De Guaricana | São José Do Pinhais |  | Brazil |  |  |  | X |  |  |  |  |  |
| *E. russatus* | MHNCI1395 |  | Represa De Guaricana | São José Do Pinhais |  | Brazil |  |  |  | X |  |  |  |  |  |
| *E. russatus* | MHNCI1398 |  | Represa De Guaricana | São José Do Pinhais |  | Brazil |  |  |  | X |  |  |  |  |  |
| *E. russatus* | MHNCI1400 |  | Represa De Guaricana | São José Do Pinhais |  | Brazil |  |  |  | X |  |  |  |  |  |
| *E. russatus* | MHNCI1406 |  | Represa De Guaricana | São José Do Pinhais |  | Brazil |  |  |  | X |  |  |  |  |  |
| *E. russatus* | MHNCI1408 |  | Represa De Guaricana | São José Do Pinhais |  | Brazil |  |  |  | X |  |  |  |  |  |
| *E. russatus* | MHNCI1417 |  | Represa De Guaricana | São José Do Pinhais |  | Brazil |  |  |  | X |  |  |  |  |  |
| *E. russatus* | MHNCI1418 |  | Represa De Guaricana | São José Do Pinhais |  | Brazil |  |  |  | X |  |  |  |  |  |
| *E. russatus* | MHNCI1419 |  | Represa De Guaricana | São José Do Pinhais |  | Brazil |  |  |  | X |  |  |  |  |  |
| *E. russatus* | MHNCI1420 |  | Represa De Guaricana | São José Do Pinhais |  | Brazil |  |  |  | X |  |  |  |  |  |
| *E. russatus* | MHNCI1429 |  | Represa De Guaricana | São José Do Pinhais |  | Brazil |  |  |  | X |  |  |  |  |  |
| *E. russatus* | MHNCI1431 |  | Represa De Guaricana | São José Do Pinhais |  | Brazil |  |  |  | X |  |  |  |  |  |
| *E. russatus* | MHNCI1432 |  | Represa De Guaricana | São José Do Pinhais |  | Brazil |  |  |  | X |  |  |  |  |  |
| *E. russatus* | MHNCI1437 |  | Represa De Guaricana | São José Do Pinhais |  | Brazil |  |  |  | X |  |  |  |  |  |
| *E. russatus* | MHNCI1439 |  | Represa De Guaricana | São José Do Pinhais |  | Brazil |  |  |  | X |  |  |  |  |  |
| *E. russatus* | MHNCI1440 |  | Represa De Guaricana | São José Do Pinhais |  | Brazil |  |  |  | X |  |  |  |  |  |
| *E. russatus* | MHNCI1443 |  | Represa De Guaricana | São José Do Pinhais |  | Brazil |  |  |  | X |  |  |  |  |  |
| *E. russatus* | MHNCI1450 |  | Represa De Guaricana | São José Do Pinhais |  | Brazil |  |  |  | X |  |  |  |  |  |
| *E. russatus* | MHNCI1453 |  | Represa De Guaricana | São José Do Pinhais |  | Brazil |  |  |  | X |  |  |  |  |  |
| *E. russatus* | MHNCI1455 |  | Represa De Guaricana | São José Do Pinhais |  | Brazil |  |  |  | X |  |  |  |  |  |
| *E. russatus* | MHNCI1456 |  | Represa De Guaricana | São José Do Pinhais |  | Brazil |  |  |  | X |  |  |  |  |  |
| *E. russatus* | MHNCI1457 |  | Represa De Guaricana | São José Do Pinhais |  | Brazil |  |  |  | X |  |  |  |  |  |
| *E. russatus* | MHNCI1474 |  | Represa De Guaricana | São José Do Pinhais |  | Brazil |  |  |  | X |  |  |  |  |  |
| *E. russatus* | MHNCI1477 |  | Represa De Guaricana | São José Do Pinhais |  | Brazil |  |  |  | X |  |  |  |  |  |
| *E. russatus* | MHNCI1478 |  | Represa De Guaricana | São José Do Pinhais |  | Brazil |  |  |  | X |  |  |  |  |  |
| *E. russatus* | MHNCI1480 |  | Represa De Guaricana | São José Do Pinhais |  | Brazil |  |  |  | X |  |  |  |  |  |
| *E. russatus* | MHNCI1483 |  | Represa De Guaricana | São José Do Pinhais |  | Brazil |  |  |  | X |  |  |  |  |  |
| *E. russatus* | MHNCI1484 |  | Represa De Guaricana | São José Do Pinhais |  | Brazil |  |  |  | X |  |  |  |  |  |
| *E. russatus* | MHNCI1496 |  | Represa De Guaricana | São José Do Pinhais |  | Brazil |  |  |  | X |  |  |  |  |  |
| *E. russatus* | MHNCI1501 |  | Represa De Guaricana | São José Do Pinhais |  | Brazil |  |  |  | X |  |  |  |  |  |
| *E. russatus* | MHNCI1514 |  | Represa De Guaricana | São José Do Pinhais |  | Brazil |  |  |  | X |  |  |  |  |  |
| *E. russatus* | MHNCI1520 |  | Represa De Guaricana | São José Do Pinhais |  | Brazil |  |  |  | X |  |  |  |  |  |
| *E. russatus* | MHNCI1534 |  | Represa De Guaricana | São José Do Pinhais |  | Brazil |  |  |  | X |  |  |  |  |  |
| *E. russatus* | MHNCI1539 |  | Represa De Guaricana | São José Do Pinhais |  | Brazil |  |  |  | X |  |  |  |  |  |
| *E. russatus* | MHNCI1543 |  | Represa De Guaricana | São José Do Pinhais |  | Brazil |  |  |  | X |  |  |  |  |  |
| *E. russatus* | MHNCI1554 |  | Represa De Guaricana | São José Do Pinhais |  | Brazil |  |  |  | X |  |  |  |  |  |
| *E. russatus* | MHNCI1555 |  | Represa De Guaricana | São José Do Pinhais |  | Brazil |  |  |  | X |  |  |  |  |  |
| *E. russatus* | MHNCI1557 |  | Represa De Guaricana | São José Do Pinhais |  | Brazil |  |  |  | X |  |  |  |  |  |
| *E. russatus* | MHNCI1560 |  | Represa De Guaricana | São José Do Pinhais |  | Brazil |  |  |  | X |  |  |  |  |  |
| *E. russatus* | MHNCI1567 |  | Represa De Guaricana | São José Do Pinhais |  | Brazil |  |  |  | X |  |  |  |  |  |
| *E. russatus* | MHNCI1569 |  | Represa De Guaricana | São José Do Pinhais |  | Brazil |  |  |  | X |  |  |  |  |  |
| *E. russatus* | MHNCI1575 |  | Represa De Guaricana | São José Do Pinhais |  | Brazil |  |  |  | X |  |  |  |  |  |
| *E. russatus* | MHNCI1579 |  | Represa De Guaricana | São José Do Pinhais |  | Brazil |  |  |  | X |  |  |  |  |  |
| *E. russatus* | MHNCI1580 |  | Represa De Guaricana | São José Do Pinhais |  | Brazil |  |  |  | X |  |  |  |  |  |
| *E. russatus* | MHNCI1584 |  | Represa De Guaricana | São José Do Pinhais |  | Brazil |  |  |  | X |  |  |  |  |  |
| *E. russatus* | MHNCI1591 |  | Represa De Guaricana | São José Do Pinhais |  | Brazil |  |  |  | X |  |  |  |  |  |
| *E. russatus* | MHNCI1592 |  | Represa De Guaricana | São José Do Pinhais |  | Brazil |  |  |  | X |  |  |  |  |  |
| *E. russatus* | MHNCI1594 |  | Represa De Guaricana | São José Do Pinhais |  | Brazil |  |  |  | X |  |  |  |  |  |
| *E. russatus* | MHNCI1596 |  | Represa De Guaricana | São José Do Pinhais |  | Brazil |  |  |  | X |  |  |  |  |  |
| *E. russatus* | MHNCI1598 |  | Represa De Guaricana | São José Do Pinhais |  | Brazil |  |  |  | X |  |  |  |  |  |
| *E. russatus* | MHNCI1599 |  | Represa De Guaricana | São José Do Pinhais |  | Brazil |  |  |  | X |  |  |  |  |  |
| *E. russatus* | MHNCI1600 |  | Represa De Guaricana | São José Do Pinhais |  | Brazil |  |  |  | X |  |  |  |  |  |
| *E. russatus* | MHNCI1601 |  | Represa De Guaricana | São José Do Pinhais |  | Brazil |  |  |  | X |  |  |  |  |  |
| *E. russatus* | MHNCI1603 |  | Represa De Guaricana | São José Do Pinhais |  | Brazil |  |  |  | X |  |  |  |  |  |
| *E. russatus* | MHNCI1604 |  | Represa De Guaricana | São José Do Pinhais |  | Brazil |  |  |  | X |  |  |  |  |  |
| *E. russatus* | MHNCI1606 |  | Represa De Guaricana | São José Do Pinhais |  | Brazil |  |  |  | X |  |  |  |  |  |
| *E. russatus* | MHNCI1610 |  | Represa De Guaricana | São José Do Pinhais |  | Brazil |  |  |  | X |  |  |  |  |  |
| *E. russatus* | MHNCI1615 |  | Represa De Guaricana | São José Do Pinhais |  | Brazil |  |  |  | X |  |  |  |  |  |
| *E. russatus* | MHNCI1616 |  | Represa De Guaricana | São José Do Pinhais |  | Brazil |  |  |  | X |  |  |  |  |  |
| *E. russatus* | MHNCI1617 |  | Represa De Guaricana | São José Do Pinhais |  | Brazil |  |  |  | X |  |  |  |  |  |
| *E. russatus* | MHNCI1619 |  | Represa De Guaricana | São José Do Pinhais |  | Brazil |  |  |  | X |  |  |  |  |  |
| *E. russatus* | MHNCI1621 |  | Represa De Guaricana | São José Do Pinhais |  | Brazil |  |  |  | X |  |  |  |  |  |
| *E. russatus* | MHNCI1623 |  | Represa De Guaricana | São José Do Pinhais |  | Brazil |  |  |  | X |  |  |  |  |  |
| *E. russatus* | MHNCI1629 |  | Represa De Guaricana | São José Do Pinhais |  | Brazil |  |  |  | X |  |  |  |  |  |
| *E. russatus* | MHNCI1632 |  | Represa De Guaricana | São José Do Pinhais |  | Brazil |  |  |  | X |  |  |  |  |  |
| *E. russatus* | MHNCI1636 |  | Represa De Guaricana | São José Do Pinhais |  | Brazil |  |  |  | X |  |  |  |  |  |
| *E. russatus* | MHNCI1637 |  | Represa De Guaricana | São José Do Pinhais |  | Brazil |  |  |  | X |  |  |  |  |  |
| *E. russatus* | MHNCI1638 |  | Represa De Guaricana | São José Do Pinhais |  | Brazil |  |  |  | X |  |  |  |  |  |
| *E. russatus* | MHNCI1640 |  | Represa De Guaricana | São José Do Pinhais |  | Brazil |  |  |  | X |  |  |  |  |  |
| *E. russatus* | MHNCI1641 |  | Represa De Guaricana | São José Do Pinhais |  | Brazil |  |  |  | X |  |  |  |  |  |
| *E. russatus* | MHNCI1644 |  | Represa De Guaricana | São José Do Pinhais |  | Brazil |  |  |  | X |  |  |  |  |  |
| *E. russatus* | MHNCI1648 |  | Represa De Guaricana | São José Do Pinhais |  | Brazil |  |  |  | X |  |  |  |  |  |
| *E. russatus* | MHNCI1650 |  | Represa De Guaricana | São José Do Pinhais |  | Brazil |  |  |  | X |  |  |  |  |  |
| *E. russatus* | MHNCI1651 |  | Represa De Guaricana | São José Do Pinhais |  | Brazil |  |  |  | X |  |  |  |  |  |
| *E. russatus* | MHNCI1652 |  | Represa De Guaricana | São José Do Pinhais |  | Brazil |  |  |  | X |  |  |  |  |  |
| *E. russatus* | MHNCI1656 |  | Represa De Guaricana | São José Do Pinhais |  | Brazil |  |  |  | X |  |  |  |  |  |
| *E. russatus* | MHNCI1659 |  | Represa De Guaricana | São José Do Pinhais |  | Brazil |  |  |  | X |  |  |  |  |  |
| *E. russatus* | MHNCI1663 |  | Represa De Guaricana | São José Do Pinhais |  | Brazil |  |  |  | X |  |  |  |  |  |
| *E. russatus* | MHNCI1664 |  | Represa De Guaricana | São José Do Pinhais |  | Brazil |  |  |  | X |  |  |  |  |  |
| *E. russatus* | MHNCI1665 |  | Represa De Guaricana | São José Do Pinhais |  | Brazil |  |  |  | X |  |  |  |  |  |
| *E. russatus* | MHNCI2005 |  | Represa De Guaricana | São José Do Pinhais |  | Brazil |  |  |  | X |  |  |  |  |  |
| *E. russatus* | MHNCI2431 |  | Represa De Guaricana | São José Do Pinhais |  | Brazil |  |  |  | X |  |  |  |  |  |
| *E. russatus* | MHNCI2446 |  | Represa De Guaricana | São José Do Pinhais |  | Brazil |  |  |  | X |  |  |  |  |  |
| *E. russatus* | UFPR-P276 |  | Represa De Guaricana | São José Do Pinhais |  | Brazil |  |  |  | X |  |  |  |  |  |
| *E. russatus* | UFPR-P667 |  | Represa De Guaricana | São José Do Pinhais |  | Brazil |  |  |  | X |  |  |  |  |  |
| *E. russatus* | UFPR-P703 |  | Represa De Guaricana | São José Do Pinhais |  | Brazil |  |  |  | X |  |  |  |  |  |
| *E. russatus* | UFPR-P753 |  | Represa De Guaricana | São José Do Pinhais |  | Brazil |  |  |  | X |  |  |  |  |  |
| *E. russatus* | UFPR-P759 |  | Represa De Guaricana | São José Do Pinhais |  | Brazil |  |  |  | X |  |  |  |  |  |
| *E. russatus* | UFPR-P767 |  | Represa De Guaricana | São José Do Pinhais |  | Brazil |  |  |  | X |  |  |  |  |  |
| *E. russatus* | UFPR-P770 |  | Represa De Guaricana | São José Do Pinhais |  | Brazil |  |  |  | X |  |  |  |  |  |
| *E. russatus* | UFPR-P772 |  | Represa De Guaricana | São José Do Pinhais |  | Brazil |  |  |  | X |  |  |  |  |  |
| *E. russatus* | UFPR-P774 |  | Represa De Guaricana | São José Do Pinhais |  | Brazil |  |  |  | X |  |  |  |  |  |
| *E. russatus* | UFPR-P803 |  | Represa De Guaricana | São José Do Pinhais |  | Brazil |  |  |  | X |  |  |  |  |  |
| *E. russatus* | UFPR-P804 |  | Represa De Guaricana | São José Do Pinhais |  | Brazil |  |  |  | X |  |  |  |  |  |
| ***E. russatus*** |  | **IIM298** | **Wenceslau Brás** | **Wenceslau Brás** |  | **Brazil** | **-23.85** | **-49.8** | **95** |  | **X** | **MT118068** | **X** | **MT118092** | **This study** |
| ***E. russatus*** |  | **IIM300** | **Wenceslau Brás** | **Wenceslau Brás** |  | **Brazil** | **-23.85** | **-49.8** | **95** |  | **X** | **MT118069** | **X** | **MT118093** | **This study** |
| *E. russatus* | LP31 |  | Ilha Grande | Angra Dos Reis | Rio de Janeiro | Brazil |  |  |  | X |  |  |  |  |  |
| *E. russatus* | LP32 |  | Ilha Grande | Angra Dos Reis | Rio de Janeiro | Brazil |  |  |  | X |  |  |  |  |  |
| *E. russatus* | LV-MAM12 |  | Ilha Grande, P. Vermelha | Angra Dos Reis | Rio de Janeiro | Brazil |  |  |  | X |  |  |  |  |  |
| *E. russatus* | LV-MAM23 |  | Ilha Grande, P Vermelha | Angra Dos Reis | Rio de Janeiro | Brazil |  |  |  | X |  |  |  |  |  |
| *E. russatus* | LV-MAM26 |  | Ilha Grande, P Vermelha | Angra Dos Reis | Rio de Janeiro | Brazil |  |  |  | X |  |  |  |  |  |
| *E. russatus* | LV-MAM28 |  | Ilha Grande, P. Vermelha | Angra Dos Reis | Rio de Janeiro | Brazil |  |  |  | X |  |  |  |  |  |
| *E. russatus* | LV-MAM3 |  | Ilha Grande, P Vermelha | Angra Dos Reis | Rio de Janeiro | Brazil |  |  |  | X |  |  |  |  |  |
| *E. russatus* | MNRJ24074 |  | Ilha Grande, P Vermelha | Angra Dos Reis | Rio de Janeiro | Brazil |  |  |  | X |  |  |  |  |  |
| *E. russatus* | MNRJ24075 |  | Ilha Grande, P Vermelha | Angra Dos Reis | Rio de Janeiro | Brazil |  |  |  | X |  |  |  |  |  |
| *E. russatus* | MNRJ24364 |  | Ilha Grande, P Vermelha | Angra Dos Reis | Rio de Janeiro | Brazil |  |  |  | X |  |  |  |  |  |
| *E. russatus* | MNRJ24370 |  | Ilha Grande | Angra Dos Reis | Rio de Janeiro | Brazil |  |  |  | X |  |  |  |  |  |
| *E. russatus* | MNRJ24373 |  | Ilha Grande | Angra Dos Reis | Rio de Janeiro | Brazil |  |  |  | X |  |  |  |  |  |
| *E. russatus* | MNRJ24374 |  | Ilha Grande | Angra Dos Reis | Rio de Janeiro | Brazil |  |  |  | X |  |  |  |  |  |
| *E. russatus* | LP14 |  | Mambucaba, Rio Do Coronel | Angra Dos Reis | Rio de Janeiro | Brazil |  |  |  | X |  |  |  |  |  |
| *E. russatus* | LP18 |  | Mambucaba, Casa Ibama | Angra Dos Reis | Rio de Janeiro | Brazil |  |  |  | X |  |  |  |  |  |
| *E. russatus* | LP19 |  | Mambucaba, Rio Do Coronel | Angra Dos Reis | Rio de Janeiro | Brazil |  |  |  | X |  |  |  |  |  |
| *E. russatus* | LP23 |  | Mambucaba, Rio Mambucaba | Angra Dos Reis | Rio de Janeiro | Brazil |  |  |  | X |  |  |  |  |  |
| *E. russatus* | LP29 |  | Mambucaba, Casa Ibama | Angra Dos Reis | Rio de Janeiro | Brazil |  |  |  | X |  |  |  |  |  |
| *E. russatus* | LV-CRB850 |  | Vila Dois Rios, Ilha Grande | Angra Dos Reis | Rio de Janeiro | Brazil |  |  |  | X |  |  |  |  |  |
| *E. russatus* | LV-CRB851 |  | Vila Dois Rios, Ilha Grande | Angra Dos Reis | Rio de Janeiro | Brazil |  |  |  | X |  |  |  |  |  |
| *E. russatus* | LV-GL30 |  | Vila Dois Rios, Ilha Grande | Angra Dos Reis | Rio de Janeiro | Brazil |  |  |  | X |  |  |  |  |  |
| *E. russatus* | LV-GL31 |  | Vila Dois Rios, Ilha Grande | Angra Dos Reis | Rio de Janeiro | Brazil |  |  |  | X |  |  |  |  |  |
| *E. russatus* | LV-GL34 |  | Vila Dois Rios, Ilha Grande | Angra Dos Reis | Rio de Janeiro | Brazil |  |  |  | X |  |  |  |  |  |
| *E. russatus* | LBCE311 |  | Vila Dois Rios, Ilha Grande | Angra Dos Reis | Rio de Janeiro | Brazil |  |  |  | X |  |  |  |  |  |
| *E. russatus* | LBCE313 |  | Vila Dois Rios, Ilha Grande | Angra Dos Reis | Rio de Janeiro | Brazil |  |  |  | X |  |  |  |  |  |
| *E. russatus* | LP09 |  | Vila Dois Rios, Ilha Grande | Angra Dos Reis | Rio de Janeiro | Brazil |  |  |  | X |  |  |  |  |  |
| *E. russatus* | LP11 |  | Vila Dois Rios, Ilha Grande | Angra Dos Reis | Rio de Janeiro | Brazil |  |  |  | X |  |  |  |  |  |
| *E. russatus* | LP13 |  | Vila Dois Rios, Ilha Grande | Angra Dos Reis | Rio de Janeiro | Brazil |  |  |  | X |  |  |  |  |  |
| *E. russatus* | MNRJ24375 |  | Glicerio | Macae | Rio de Janeiro | Brazil |  |  |  | X |  |  |  |  |  |
| *E. russatus* | MNRJ24390 |  | Glicerio | Macae | Rio de Janeiro | Brazil |  |  |  | X |  |  |  |  |  |
| *E. russatus* | MNRJ24391 |  | Glicerio | Macae | Rio de Janeiro | Brazil |  |  |  | X |  |  |  |  |  |
| *E. russatus* | MNRJ24392 |  | Centro De Primatologia | Magé | Rio de Janeiro | Brazil |  |  |  | X |  |  |  |  |  |
| *E. russatus* | MNRJ25030 |  | Centro De Primatologia | Magé | Rio de Janeiro | Brazil |  |  |  | X |  |  |  |  |  |
| *E. russatus* | MNRJ35891 |  | Centro De Primatologia | Magé | Rio de Janeiro | Brazil |  |  |  | X |  |  |  |  |  |
| *E. russatus* | MNRJ35892 |  | Centro De Primatologia | Magé | Rio de Janeiro | Brazil |  |  |  | X |  |  |  |  |  |
| *E. russatus* | MNRJ5296 |  | Serra Do Tinguá | Nova Iguaçu | Rio de Janeiro | Brazil |  |  |  | X |  |  |  |  |  |
| *E. russatus* | MNRJ5824 |  | Pedra Branca | Parati | Rio de Janeiro | Brazil |  |  |  | X |  |  |  |  |  |
| *E. russatus* | MNRJ6210 |  | Pedra Branca | Parati | Rio de Janeiro | Brazil |  |  |  | X |  |  |  |  |  |
| *E. russatus* | MNRJ6321 |  | Pedra Branca | Parati | Rio de Janeiro | Brazil |  |  |  | X |  |  |  |  |  |
| *E. russatus* | MNRJ6322 |  | Pedra Branca | Parati | Rio de Janeiro | Brazil |  |  |  | X |  |  |  |  |  |
| *E. russatus* | MNRJ6462 |  | Pedra Branca | Parati | Rio de Janeiro | Brazil |  |  |  | X |  |  |  |  |  |
| *E. russatus* | MNRJ7070 |  | Pedra Branca | Parati | Rio de Janeiro | Brazil |  |  |  | X |  |  |  |  |  |
| *E. russatus* | MNRJ7071 |  | Pedra Branca | Parati | Rio de Janeiro | Brazil |  |  |  | X |  |  |  |  |  |
| *E. russatus* | MNRJ7073 |  | Pedra Branca | Parati | Rio de Janeiro | Brazil |  |  |  | X |  |  |  |  |  |
| *E. russatus* | MNRJ7074 |  | Pedra Branca | Parati | Rio de Janeiro | Brazil |  |  |  | X |  |  |  |  |  |
| *E. russatus* | MNRJ8155 |  | Pedra Branca | Parati | Rio de Janeiro | Brazil |  |  |  | X |  |  |  |  |  |
| *E. russatus* | MNRJ8156 |  | Pedra Branca | Parati | Rio de Janeiro | Brazil |  |  |  | X |  |  |  |  |  |
| *E. russatus* | MNRJ8157 |  | Pedra Branca | Paratí | Rio de Janeiro | Brazil |  |  |  | X |  |  |  |  |  |
| *E. russatus* | MNRJ8158 |  | Pedra Branca | Paratí | Rio de Janeiro | Brazil |  |  |  | X |  |  |  |  |  |
| *E. russatus* | MNRJ8215 |  | Desengano, Rib Vermelho | Santa Maria Madalena | Rio de Janeiro | Brazil |  |  |  | X |  |  |  |  |  |
| *E. russatus* | MNRJ8248 |  | Desengano, Mata Da Rita | Santa Maria Madalena | Rio de Janeiro | Brazil |  |  |  | X |  |  |  |  |  |
| *E. russatus* | MNRJ8421 |  | Desengano, Mata Da Rita | Santa Maria Madalena | Rio de Janeiro | Brazil |  |  |  | X |  |  |  |  |  |
| *E. russatus* | MNRJ8422 |  | Faz. Tenente | São João Marcos | Rio de Janeiro | Brazil |  |  |  | X |  |  |  |  |  |
| *E. russatus* | MNRJ8423 |  | Fazenda Guinle | Teresópolis | Rio de Janeiro | Brazil |  |  |  | X |  |  |  |  |  |
| *E. russatus* | MNRJ8424 |  | Fazenda Guinle | Teresópolis | Rio de Janeiro | Brazil |  |  |  | X |  |  |  |  |  |
| *E. russatus* | MNRJ8425 |  | Fazenda Guinle | Teresópolis | Rio de Janeiro | Brazil |  |  |  | X |  |  |  |  |  |
| *E. russatus* | MZUSP1969 |  | Fazenda Guinle | Teresópolis | Rio de Janeiro | Brazil |  |  |  | X |  |  |  |  |  |
| *E. russatus* | MZUSP1970 |  | Fazenda Guinle | Teresópolis | Rio de Janeiro | Brazil |  |  |  | X |  |  |  |  |  |
| *E. russatus* | MZUSP1971 |  | Fazenda Guinle | Teresópolis | Rio de Janeiro | Brazil |  |  |  | X |  |  |  |  |  |
| *E. russatus* | MPEG23540 |  | Barracão |  | Rio Grande do Sul | Brazil |  |  |  | X |  |  |  |  |  |
| *E. russatus* | MPEG22249 |  | Lagoa Do Jacaré | Torres | Rio Grande do Sul | Brazil |  |  |  | X |  |  |  |  |  |
| *E. russatus* | MZUSP22513 |  | Corupa |  | Santa Catarina | Brazil |  |  |  | X |  |  |  |  |  |
| *E. russatus* | MZUSP586 |  | Corupa |  | Santa Catarina | Brazil |  |  |  | X |  |  |  |  |  |
| *E. russatus* | MZUSP853 |  | Corupa |  | Santa Catarina | Brazil |  |  |  | X |  |  |  |  |  |
| *E. russatus* | MZUSP854 |  | Corupa |  | Santa Catarina | Brazil |  |  |  | X |  |  |  |  |  |
| *E. russatus* | UFSC713 |  | Parque Estadual Da Serra Do Tabuleiro | Caldas Da Imperatriz | Santa Catarina | Brazil |  |  |  | X |  |  |  |  |  |
| *E. russatus* | UFSC714 |  | Parque Estadual Da Serra Do Tabuleiro | Caldas Da Imperatriz | Santa Catarina | Brazil |  |  |  | X |  |  |  |  |  |
| *E. russatus* | UFSC717 |  | Parque Estadual Da Serra Do Tabuleiro | Caldas Da Imperatriz | Santa Catarina | Brazil |  |  |  | X |  |  |  |  |  |
| *E. russatus* | UFSC719 |  | Parque Estadual Da Serra Do Tabuleiro | Caldas Da Imperatriz | Santa Catarina | Brazil |  |  |  | X |  |  |  |  |  |
| *E. russatus* | UFSC720 |  | Parque Estadual Da Serra Do Tabuleiro | Caldas Da Imperatriz | Santa Catarina | Brazil |  |  |  | X |  |  |  |  |  |
| *E. russatus* | UFSC729 |  | Parque Estadual Da Serra Do Tabuleiro | Caldas Da Imperatriz | Santa Catarina | Brazil |  |  |  | X |  |  |  |  |  |
| *E. russatus* | UFSC511 |  | Rio Cubatão | Joinvile | Santa Catarina | Brazil |  |  |  | X |  |  |  |  |  |
| *E. russatus* | UFSC512 |  | Rio Cubatão | Joinvile | Santa Catarina | Brazil |  |  |  | X |  |  |  |  |  |
| *E. russatus* | UFSC513 |  | Rio Cubatão | Joinvile | Santa Catarina | Brazil |  |  |  | X |  |  |  |  |  |
| *E. russatus* | UFSC514 |  | Rio Cubatão | Joinvile | Santa Catarina | Brazil |  |  |  | X |  |  |  |  |  |
| *E. russatus* | UFSC515 |  | Rio Cubatão | Joinvile | Santa Catarina | Brazil |  |  |  | X |  |  |  |  |  |
| *E. russatus* | UFSC516 |  | Rio Cubatão | Joinvile | Santa Catarina | Brazil |  |  |  | X |  |  |  |  |  |
| *E. russatus* | CEM3717 |  | Bauru | Bauru | São Paulo | Brazil |  |  |  | X |  |  |  |  |  |
| ***E. russatus*** |  | **CIT1755** | **Biritiba-Mirim** | **Biritiba-Mirim** |  | **Brazil** | **-23.58** | **-46.03** | **96** |  | **X** | **MT118064** | **X** | **MT118089** | **This study** |
| ***E. russatus*** |  | **UNIBAN2022** | **Biritiba-Mirim** | **Biritiba-Mirim** |  | **Brazil** | **-23.58** | **-46.03** | **96** |  | **X** | **MT118071** | **X** | **MT118095** | **This study** |
| *E. russatus* | CEM729 |  | Cananeia | Cananeia | São Paulo | Brazil |  |  |  | X |  |  |  |  |  |
| *E. russatus* | UFMG-MAM112 |  | Cananéia | Cananéia | São Paulo | Brazil |  |  |  | X |  |  |  |  |  |
| *E. russatus* | UFMG-MAM113 |  | Cananéia | Cananéia | São Paulo | Brazil |  |  |  | X |  |  |  |  |  |
| *E. russatus* | UFMG-MAM114 |  | Cananéia | Cananéia | São Paulo | Brazil |  |  |  | X |  |  |  |  |  |
| *E. russatus* | UFMG-MAM129 |  | Cananéia | Cananéia | São Paulo | Brazil |  |  |  | X |  |  |  |  |  |
| *E. russatus* | UFMG-MAM130 |  | Cananéia | Cananéia | São Paulo | Brazil |  |  |  | X |  |  |  |  |  |
| *E. russatus* | UFMG-MAM131 |  | Cananéia | Cananéia | São Paulo | Brazil |  |  |  | X |  |  |  |  |  |
| *E. russatus* | UFMG-MAM144 |  | Cananéia | Cananéia | São Paulo | Brazil |  |  |  | X |  |  |  |  |  |
| *E. russatus* | UFMG-MAM172 |  | Cananéia | Cananéia | São Paulo | Brazil |  |  |  | X |  |  |  |  |  |
| *E. russatus* | UFMG-MAM175 |  | Cananéia | Cananéia | São Paulo | Brazil |  |  |  | X |  |  |  |  |  |
| *E. russatus* | UFMG-MAM176 |  | Cananéia | Cananéia | São Paulo | Brazil |  |  |  | X |  |  |  |  |  |
| *E. russatus* | UFMG-MAM178 |  | Fazenda Intervales, Base Do Carmo | Capao Bonito | São Paulo | Brazil |  |  |  | X |  |  |  |  |  |
| *E. russatus* | UFMG-MAM180 |  | Fazenda Intervales, Base Do Carmo | Capao Bonito | São Paulo | Brazil |  |  |  | X |  |  |  |  |  |
| *E. russatus* | UFMG-MAM181 |  | Fazenda Intervales, Base Do Carmo | Capao Bonito | São Paulo | Brazil |  |  |  | X |  |  |  |  |  |
| *E. russatus* | UFMG-MAM183 |  | Fazenda Intervales, Base Do Carmo | Capao Bonito | São Paulo | Brazil |  |  |  | X |  |  |  |  |  |
| *E. russatus* | UFMG-MAM184 |  | Fazenda Intervales, Base Do Carmo | Capao Bonito | São Paulo | Brazil |  |  |  | X |  |  |  |  |  |
| *E. russatus* | MVZ-MAM261 |  | Fazenda Intervales, Base Do Carmo | Capao Bonito | São Paulo | Brazil |  |  |  | X |  |  |  |  |  |
| *E. russatus* | MVZ-MAM264 |  | Fazenda Intervales, Base Do Carmo | Capao Bonito | São Paulo | Brazil |  |  |  | X |  |  |  |  |  |
| *E. russatus* | UFMG-MAM28 |  | Fazenda Intervales, Base Do Carmo | Capao Bonito | São Paulo | Brazil |  |  |  | X |  |  |  |  |  |
| *E. russatus* | MVZ-MAM286 |  | Fazenda Intervales, Base Do Carmo | Capao Bonito | São Paulo | Brazil |  |  |  | X |  |  |  |  |  |
| *E. russatus* | MVZ-MAM294 |  | Fazenda Intervales, Base Do Carmo | Capao Bonito | São Paulo | Brazil |  |  |  | X |  |  |  |  |  |
| *E. russatus* | MVZ-MAM295 |  | Fazenda Intervales, Base Saibadela | Capao Bonito | São Paulo | Brazil |  |  |  | X |  |  |  |  |  |
| *E. russatus* | MVZ-MAM296 |  | Fazenda Intervales, Base Saibadela | Capao Bonito | São Paulo | Brazil |  |  |  | X |  |  |  |  |  |
| *E. russatus* | MVZ-MAM324 |  | Fazenda Intervales, Base Saibadela | Capao Bonito | São Paulo | Brazil |  |  |  | X |  |  |  |  |  |
| *E. russatus* | MVZ-MAM325 |  | Fazenda Intervales, Base Saibadela | Capao Bonito | São Paulo | Brazil |  |  |  | X |  |  |  |  |  |
| *E. russatus* | MVZ-MAM341 |  | Fazenda Intervales, Base Do Carmo | Capao Bonito | São Paulo | Brazil |  |  |  | X |  |  |  |  |  |
| *E. russatus* | MVZ-MAM345 |  | Fazenda Intervales, Base Do Carmo | Capao Bonito | São Paulo | Brazil |  |  |  | X |  |  |  |  |  |
| *E. russatus* | MVZ-MAM390 |  | Fazenda Intervales, Base Do Carmo | Capao Bonito | São Paulo | Brazil |  |  |  | X |  |  |  |  |  |
| *E. russatus* | MVZ-MAM394 |  | Fazenda Intervales, Base Do Carmo | Capao Bonito | São Paulo | Brazil |  |  |  | X |  |  |  |  |  |
| *E. russatus* | MVZ-MAM419 |  | Fazenda Intervales, Base Do Carmo | Capao Bonito | São Paulo | Brazil |  |  |  | X |  |  |  |  |  |
| *E. russatus* | MVZ-MAM420 |  | Fazenda Intervales, Base Do Carmo | Capao Bonito | São Paulo | Brazil |  |  |  | X |  |  |  |  |  |
| *E. russatus* | MVZ-MAM433 |  | Fazenda Intervales, Base Do Carmo | Capao Bonito | São Paulo | Brazil |  |  |  | X |  |  |  |  |  |
| *E. russatus* | MVZ-MAM438 |  | Fazenda Intervales, Base Do Carmo | Capao Bonito | São Paulo | Brazil |  |  |  | X |  |  |  |  |  |
| *E. russatus* | MVZ-MAM444 |  | Fazenda Intervales, Base Do Carmo | Capao Bonito | São Paulo | Brazil |  |  |  | X |  |  |  |  |  |
| *E. russatus* | MVZ-MAM456 |  | Fazenda Intervales, Base Do Carmo | Capao Bonito | São Paulo | Brazil |  |  |  | X |  |  |  |  |  |
| *E. russatus* | UFMG-MAM67 |  | Fazenda Intervales, Base Do Carmo | Capao Bonito | São Paulo | Brazil |  |  |  | X |  |  |  |  |  |
| *E. russatus* | UFMG-MAM68 |  | Fazenda Intervales, Base Do Carmo | Capao Bonito | São Paulo | Brazil |  |  |  | X |  |  |  |  |  |
| *E. russatus* | UFMG-MAM69 |  | Fazenda Intervales | Capao Bonito | São Paulo | Brazil |  |  |  | X |  |  |  |  |  |
| *E. russatus* | UFMG-MAM70 |  | Fazenda Intervales | Capao Bonito | São Paulo | Brazil |  |  |  | X |  |  |  |  |  |
| *E. russatus* | UFMG-MAM86 |  | Fazenda Intervales | Capao Bonito | São Paulo | Brazil |  |  |  | X |  |  |  |  |  |
| *E. russatus* | UFMG-MAM874 |  | Fazenda Intervales | Capao Bonito | São Paulo | Brazil |  |  |  | X |  |  |  |  |  |
| *E. russatus* | MNRJ24413 |  | Fazenda Intervales | Capao Bonito | São Paulo | Brazil |  |  |  | X |  |  |  |  |  |
| *E. russatus* | MNRJ24415 |  | Fazenda Intervales | Capao Bonito | São Paulo | Brazil |  |  |  | X |  |  |  |  |  |
| *E. russatus* | MNRJ24417 |  | Fazenda Intervales | Capao Bonito | São Paulo | Brazil |  |  |  | X |  |  |  |  |  |
| *E. russatus* | MNRJ24441 |  | Fazenda Intervales | Capao Bonito | São Paulo | Brazil |  |  |  | X |  |  |  |  |  |
| *E. russatus* | MNRJ32449 |  | Fazenda Intervales | Capao Bonito | São Paulo | Brazil |  |  |  | X |  |  |  |  |  |
| *E. russatus* | MNRJ32460 |  | Fazenda Intervales | Capao Bonito | São Paulo | Brazil |  |  |  | X |  |  |  |  |  |
| *E. russatus* | MNRJ32461 |  | Cotia | Cotia | São Paulo | Brazil |  |  |  | X |  |  |  |  |  |
| *E. russatus* | MNRJ32463 |  | Cotia | Cotia | São Paulo | Brazil |  |  |  | X |  |  |  |  |  |
| *E. russatus* | MNRJ32466 |  | Cotia | Cotia | São Paulo | Brazil |  |  |  | X |  |  |  |  |  |
| *E. russatus* | MNRJ32736 |  | Cotia | Cotia | São Paulo | Brazil |  |  |  | X |  |  |  |  |  |
| *E. russatus* | MNRJ32740 |  | Cotia | Cotia | São Paulo | Brazil |  |  |  | X |  |  |  |  |  |
| *E. russatus* | MNRJ32741 |  | Iguape | Iguape | São Paulo | Brazil |  |  |  | X |  |  |  |  |  |
| *E. russatus* | MNRJ32742 |  | Iguape (Costão Dos Engenhos) | Iguape | São Paulo | Brazil |  |  |  | X |  |  |  |  |  |
| *E. russatus* | MNRJ5281 |  | Iguape (Costão Dos Engenhos) | Iguape | São Paulo | Brazil |  |  |  | X |  |  |  |  |  |
| *E. russatus* | MNRJ5283 |  | Iguape (Costão Dos Engenhos) | Iguape | São Paulo | Brazil |  |  |  | X |  |  |  |  |  |
| *E. russatus* | MNRJ5287 |  | Iguape | Iguape | São Paulo | Brazil |  |  |  | X |  |  |  |  |  |
| *E. russatus* | MNRJ5302 |  | Iguape | Iguape | São Paulo | Brazil |  |  |  | X |  |  |  |  |  |
| *E. russatus* | MNRJ5307 |  | Iguape (Costão Dos Engenhos) | Iguape | São Paulo | Brazil |  |  |  | X |  |  |  |  |  |
| *E. russatus* | MVZ182809 |  | Fazenda Da Jaca, Ilha De São Sebastiao | Ilhabela | São Paulo | Brazil |  |  |  | X |  |  |  |  |  |
| *E. russatus* | MVZ182810 |  | Fazenda Da Jaca, Ilha De São Sebastiao | Ilhabela | São Paulo | Brazil |  |  |  | X |  |  |  |  |  |
| *E. russatus* | MVZ182811 |  | Fazenda Da Jaca, Ilha De São Sebastiao | Ilhabela | São Paulo | Brazil |  |  |  | X |  |  |  |  |  |
| *E. russatus* | MVZ182812 |  | Fazenda Da Jaca, Ilha De São Sebastiao | Ilhabela | São Paulo | Brazil |  |  |  | X |  |  |  |  |  |
| *E. russatus* | MVZ182813 |  | Ilha De São Sebastiao | Ilhabela | São Paulo | Brazil |  |  |  | X |  |  |  |  |  |
| *E. russatus* | MVZ182814 |  | Ilha De São Sebastiao | Ilhabela | São Paulo | Brazil |  |  |  | X |  |  |  |  |  |
| *E. russatus* | MVZ182815 |  | Ilha De São Sebastiao | Ilhabela | São Paulo | Brazil |  |  |  | X |  |  |  |  |  |
| *E. russatus* | MVZ182816 |  | Ilha De São Sebastiao | Ilhabela | São Paulo | Brazil |  |  |  | X |  |  |  |  |  |
| *E. russatus* | MVZ182817 |  | Ilha De São Sebastiao | Ilhabela | São Paulo | Brazil |  |  |  | X |  |  |  |  |  |
| *E. russatus* | MVZ183099 |  | Ilha De São Sebastiao | Ilhabela | São Paulo | Brazil |  |  |  | X |  |  |  |  |  |
| *E. russatus* | MVZ183100 |  | Ilha De São Sebastiao | Ilhabela | São Paulo | Brazil |  |  |  | X |  |  |  |  |  |
| *E. russatus* | MZUSP9581 |  | Ilha Dos Búzios |  | São Paulo | Brazil |  |  |  | X |  |  |  |  |  |
| *E. russatus* | MZUSP9744 |  | Ilha Dos Búzios |  | São Paulo | Brazil |  |  |  | X |  |  |  |  |  |
| *E. russatus* | MZUSP9834 |  | Ilha Dos Búzios |  | São Paulo | Brazil |  |  |  | X |  |  |  |  |  |
| *E. russatus* | MZUSP9845 |  | Ilha Dos Búzios |  | São Paulo | Brazil |  |  |  | X |  |  |  |  |  |
| *E. russatus* | MZUSP9861 |  | Ilha Dos Búzios |  | São Paulo | Brazil |  |  |  | X |  |  |  |  |  |
| *E. russatus* | MZUSP9878 |  | Ilha Dos Búzios |  | São Paulo | Brazil |  |  |  | X |  |  |  |  |  |
| *E. russatus* | MNRJ-M492 |  | Ilha Dos Búzios |  | São Paulo | Brazil |  |  |  | X |  |  |  |  |  |
| *E. russatus* | UFPB1134 |  | Ilha Dos Búzios |  | São Paulo | Brazil |  |  |  | X |  |  |  |  |  |
| *E. russatus* | UFPB1135 |  | Ilha Dos Búzios |  | São Paulo | Brazil |  |  |  | X |  |  |  |  |  |
| *E. russatus* | UFPB1136 |  | Ilha Dos Búzios |  | São Paulo | Brazil |  |  |  | X |  |  |  |  |  |
| *E. russatus* | UFPB1137 |  | Ilha Dos Búzios |  | São Paulo | Brazil |  |  |  | X |  |  |  |  |  |
| *E. russatus* | UFPB1138 |  | Ilha Dos Búzios |  | São Paulo | Brazil |  |  |  | X |  |  |  |  |  |
| *E. russatus* | UFPB1139 |  | Ilha Vitória |  | São Paulo | Brazil |  |  |  | X |  |  |  |  |  |
| *E. russatus* | UFPB1140 |  | Ilha Vitória |  | São Paulo | Brazil |  |  |  | X |  |  |  |  |  |
| *E. russatus* | UFPB-ZVEC65 |  | Ilha Vitória |  | São Paulo | Brazil |  |  |  | X |  |  |  |  |  |
| *E. russatus* | UFPB-ZVEC66 |  | Ilha Vitória |  | São Paulo | Brazil |  |  |  | X |  |  |  |  |  |
| *E. russatus* | UFPB-ZVEC75 |  | Lins (Campestre) |  | São Paulo | Brazil |  |  |  | X |  |  |  |  |  |
| *E. russatus* | MNRJ5310 |  | Ilha Do Cardoso | Ilha Do Cardoso | São Paulo | Brazil |  |  |  | X |  |  |  |  |  |
| *E. russatus* | MNRJ5324 |  | Ilha Do Cardoso | Ilha Do Cardoso | São Paulo | Brazil |  |  |  | X |  |  |  |  |  |
| *E. russatus* | MNRJ5368 |  | Ilha Do Cardoso | Ilha Do Cardoso | São Paulo | Brazil |  |  |  | X |  |  |  |  |  |
| *E. russatus* | MNRJ5394 |  | Ilha Do Cardoso | Ilha Do Cardoso | São Paulo | Brazil |  |  |  | X |  |  |  |  |  |
| *E. russatus* | MNRJ5413 |  | Ilha Do Cardoso | Ilha Do Cardoso | São Paulo | Brazil |  |  |  | X |  |  |  |  |  |
| *E. russatus* | MNRJ5481 |  | Ilha Do Cardoso | Ilha Do Cardoso | São Paulo | Brazil |  |  |  | X |  |  |  |  |  |
| *E. russatus* | MVZ182082 |  | Ilha Do Cardoso | Ilha Do Cardoso | São Paulo | Brazil |  |  |  | X |  |  |  |  |  |
| *E. russatus* | MVZ182085 |  | Ilha Do Cardoso | Ilha Do Cardoso | São Paulo | Brazil |  |  |  | X |  |  |  |  |  |
| *E. russatus* | MVZ182798 |  | Ilha Do Cardoso | Ilha Do Cardoso | São Paulo | Brazil |  |  |  | X |  |  |  |  |  |
| *E. russatus* | MVZ182799 |  | Ilha Do Cardoso | Ilha Do Cardoso | São Paulo | Brazil |  |  |  | X |  |  |  |  |  |
| *E. russatus* | MVZ182800 |  | Ilha Do Cardoso | Ilha Do Cardoso | São Paulo | Brazil |  |  |  | X |  |  |  |  |  |
| *E. russatus* | MVZ182801 |  | Ilha Do Cardoso | Ilha Do Cardoso | São Paulo | Brazil |  |  |  | X |  |  |  |  |  |
| *E. russatus* | MVZ182802 |  | Ilha Do Cardoso | Ilha Do Cardoso | São Paulo | Brazil |  |  |  | X |  |  |  |  |  |
| *E. russatus* | MVZ182803 |  | Ilha Do Cardoso | Ilha Do Cardoso | São Paulo | Brazil |  |  |  | X |  |  |  |  |  |
| *E. russatus* | MVZ182804 |  | Ilha Do Cardoso | Ilha Do Cardoso | São Paulo | Brazil |  |  |  | X |  |  |  |  |  |
| *E. russatus* | MVZ182805 |  | Ilha Do Cardoso | Ilha Do Cardoso | São Paulo | Brazil |  |  |  | X |  |  |  |  |  |
| *E. russatus* | MVZ182807 |  | Ilha Do Cardoso | Ilha Do Cardoso | São Paulo | Brazil |  |  |  | X |  |  |  |  |  |
| *E. russatus* | MVZ182808 |  | Ilha Do Cardoso | Ilha Do Cardoso | São Paulo | Brazil |  |  |  | X |  |  |  |  |  |
| *E. russatus* | MZUSP8859 |  | Ilha Vitória |  | São Paulo | Brazil |  |  |  | X |  |  |  |  |  |
| *E. russatus* | MVZ183101 |  | Iporanga (Bairro Da Serra) | Iporanga | São Paulo | Brazil |  |  |  | X |  |  |  |  |  |
| *E. russatus* | MVZ183103 |  | Iporanga | Iporanga | São Paulo | Brazil |  |  |  | X |  |  |  |  |  |
| ***E. russatus*** |  | **CIT1796** | **Juquitiba** | **Juquitiba** |  | **Brazil** | **-23.92** | **-47.07** | **97** |  | **X** | **MT118065** |  |  | **This study** |
| *E. russatus* | MVZ183105 |  | Praia Da Juréia | Juréia | São Paulo | Brazil |  |  |  | X |  |  |  |  |  |
| *E. russatus* | MVZ183106 |  | Fazenda Sete Lagoas | Mogi Guaçu | São Paulo | Brazil |  |  |  | X |  |  |  |  |  |
| *E. russatus* | MVZ183107 |  | Onça Parda | Onça Parda | São Paulo | Brazil |  |  |  | X |  |  |  |  |  |
| *E. russatus* | MVZ183108 |  | Paranapiacaba | Paranapiacaba | São Paulo | Brazil |  |  |  | X |  |  |  |  |  |
| *E. russatus* | MVZ183109 |  | Piquete | Piquete | São Paulo | Brazil |  |  |  | X |  |  |  |  |  |
| *E. russatus* | MVZ183110 |  | Rio Feio | Rio Feio | São Paulo | Brazil |  |  |  | X |  |  |  |  |  |
| *E. russatus* | MVZ183111 |  | Boraceia | Salesopolis | São Paulo | Brazil |  |  |  | X |  |  |  |  |  |
| *E. russatus* | MVZ183112 |  | Boraceia | Salesopolis | São Paulo | Brazil |  |  |  | X |  |  |  |  |  |
| *E. russatus* | MVZ183114 |  | Boraceia | Salesopolis | São Paulo | Brazil |  |  |  | X |  |  |  |  |  |
| *E. russatus* | MVZ183116 |  | Boraceia | Salesopolis | São Paulo | Brazil |  |  |  | X |  |  |  |  |  |
| *E. russatus* | MVZ183117 |  | Boraceia | Salesopolis | São Paulo | Brazil |  |  |  | X |  |  |  |  |  |
| *E. russatus* | MVZ183118 |  | Boraceia | Salesopolis | São Paulo | Brazil |  |  |  | X |  |  |  |  |  |
| *E. russatus* | MVZ183119 |  | Boraceia | Salesopolis | São Paulo | Brazil |  |  |  | X |  |  |  |  |  |
| *E. russatus* | MVZ183120 |  | Boraceia | Salesopolis | São Paulo | Brazil |  |  |  | X |  |  |  |  |  |
| *E. russatus* | MVZ183121 |  | Boraceia | Salesopolis | São Paulo | Brazil |  |  |  | X |  |  |  |  |  |
| *E. russatus* | MVZ183122 |  | Casa Grande | Salesopolis | São Paulo | Brazil |  |  |  | X |  |  |  |  |  |
| *E. russatus* | MVZ183123 |  | Boraceia, 3Km E, 28 Km Se Biritiba Mirim | Salesopolis | São Paulo | Brazil |  |  |  | X |  |  |  |  |  |
| *E. russatus* | MVZ183124 |  | Boraceia, 3Km E, 28 Km Se Biritiba Mirim | Salesopolis | São Paulo | Brazil |  |  |  | X |  |  |  |  |  |
| *E. russatus* | MZUSP10161 |  | Boraceia, 3Km E, 28 Km Se Biritiba Mirim | Salesopolis | São Paulo | Brazil |  |  |  | X |  |  |  |  |  |
| *E. russatus* | MZUSP10162 |  | Boraceia, 3Km E, 28 Km Se Biritiba Mirim | Salesopolis | São Paulo | Brazil |  |  |  | X |  |  |  |  |  |
| *E. russatus* | MZUSP10169 |  | Boraceia, 3Km E, 28 Km Se Biritiba Mirim | Salesopolis | São Paulo | Brazil |  |  |  | X |  |  |  |  |  |
| *E. russatus* | MZUSP10170 |  | Boraceia, 3Km E, 28 Km Se Biritiba Mirim | Salesopolis | São Paulo | Brazil |  |  |  | X |  |  |  |  |  |
| *E. russatus* | MZUSP10171 |  | Boraceia, 3Km E, 28 Km Se Biritiba Mirim | Salesopolis | São Paulo | Brazil |  |  |  | X |  |  |  |  |  |
| *E. russatus* | MZUSP10172 |  | Boraceia, 3Km E, 28 Km Se Biritiba Mirim | Salesopolis | São Paulo | Brazil |  |  |  | X |  |  |  |  |  |
| *E. russatus* | MZUSP10174 |  | Boraceia, 3Km E, 28 Km Se Biritiba Mirim | Salesopolis | São Paulo | Brazil |  |  |  | X |  |  |  |  |  |
| *E. russatus* | MZUSP10175 |  | Boraceia, 3Km E, 28 Km Se Biritiba Mirim | Salesopolis | São Paulo | Brazil |  |  |  | X |  |  |  |  |  |
| *E. russatus* | MZUSP10215 |  | Boraceia, 3Km E, 28 Km Se Biritiba Mirim | Salesopolis | São Paulo | Brazil |  |  |  | X |  |  |  |  |  |
| *E. russatus* | MZUSP1024 |  | Boraceia, 3Km E, 28 Km Se Biritiba Mirim | Salesopolis | São Paulo | Brazil |  |  |  | X |  |  |  |  |  |
| *E. russatus* | MZUSP10372 |  | Boraceia, 3Km E, 28 Km Se Biritiba Mirim | Salesopolis | São Paulo | Brazil |  |  |  | X |  |  |  |  |  |
| *E. russatus* | MZUSP10418 |  | Boraceia | Salesopolis | São Paulo | Brazil |  |  |  | X |  |  |  |  |  |
| *E. russatus* | MZUSP10422 |  | Boraceia | Salesopolis | São Paulo | Brazil |  |  |  | X |  |  |  |  |  |
| *E. russatus* | MZUSP10612 |  | Boraceia | Salesopolis | São Paulo | Brazil |  |  |  | X |  |  |  |  |  |
| *E. russatus* | MZUSP10677 |  | Boraceia | Salesopolis | São Paulo | Brazil |  |  |  | X |  |  |  |  |  |
| *E. russatus* | MZUSP10678 |  | Boraceia | Salesopolis | São Paulo | Brazil |  |  |  | X |  |  |  |  |  |
| *E. russatus* | MZUSP10679 |  | Boraceia | Salesopolis | São Paulo | Brazil |  |  |  | X |  |  |  |  |  |
| *E. russatus* | MZUSP10722 |  | Boraceia | Salesopolis | São Paulo | Brazil |  |  |  | X |  |  |  |  |  |
| *E. russatus* | MZUSP10724 |  | Boraceia | Salesopolis | São Paulo | Brazil |  |  |  | X |  |  |  |  |  |
| *E. russatus* | MZUSP10726 |  | Boraceia | Salesopolis | São Paulo | Brazil |  |  |  | X |  |  |  |  |  |
| *E. russatus* | MZUSP10728 |  | Boraceia | Salesopolis | São Paulo | Brazil |  |  |  | X |  |  |  |  |  |
| *E. russatus* | MZUSP10818 |  | Boraceia | Salesopolis | São Paulo | Brazil |  |  |  | X |  |  |  |  |  |
| *E. russatus* | MZUSP10942 |  | Boraceia | Salesopolis | São Paulo | Brazil |  |  |  | X |  |  |  |  |  |
| *E. russatus* | MZUSP11008 |  | Boraceia | Salesopolis | São Paulo | Brazil |  |  |  | X |  |  |  |  |  |
| *E. russatus* | MZUSP11010 |  | Boraceia | Salesopolis | São Paulo | Brazil |  |  |  | X |  |  |  |  |  |
| *E. russatus* | MZUSP11013 |  | Boraceia | Salesopolis | São Paulo | Brazil |  |  |  | X |  |  |  |  |  |
| *E. russatus* | MZUSP11027 |  | Boraceia | Salesopolis | São Paulo | Brazil |  |  |  | X |  |  |  |  |  |
| *E. russatus* | MZUSP11028 |  | Boraceia | Salesopolis | São Paulo | Brazil |  |  |  | X |  |  |  |  |  |
| *E. russatus* | MZUSP11038 |  | Boraceia | Salesopolis | São Paulo | Brazil |  |  |  | X |  |  |  |  |  |
| *E. russatus* | MZUSP11161 |  | Boraceia | Salesopolis | São Paulo | Brazil |  |  |  | X |  |  |  |  |  |
| *E. russatus* | MZUSP11419 |  | Boraceia | Salesopolis | São Paulo | Brazil |  |  |  | X |  |  |  |  |  |
| *E. russatus* | MZUSP11431 |  | Boraceia | Salesopolis | São Paulo | Brazil |  |  |  | X |  |  |  |  |  |
| *E. russatus* | MZUSP11441 |  | Boraceia | Salesopolis | São Paulo | Brazil |  |  |  | X |  |  |  |  |  |
| *E. russatus* | MZUSP11442 |  | Boraceia | Salesopolis | São Paulo | Brazil |  |  |  | X |  |  |  |  |  |
| *E. russatus* | MZUSP1178 |  | Boraceia | Salesopolis | São Paulo | Brazil |  |  |  | X |  |  |  |  |  |
| *E. russatus* | MZUSP13635 |  | Boraceia | Salesopolis | São Paulo | Brazil |  |  |  | X |  |  |  |  |  |
| *E. russatus* | MZUSP139 |  | Boraceia | Salesopolis | São Paulo | Brazil |  |  |  | X |  |  |  |  |  |
| *E. russatus* | MZUSP1791 |  | Boraceia | Salesopolis | São Paulo | Brazil |  |  |  | X |  |  |  |  |  |
| *E. russatus* | MZUSP1841 |  | Boraceia | Salesopolis | São Paulo | Brazil |  |  |  | X |  |  |  |  |  |
| *E. russatus* | MZUSP1842 |  | Boraceia | Salesopolis | São Paulo | Brazil |  |  |  | X |  |  |  |  |  |
| *E. russatus* | MZUSP1843 |  | Boraceia | Salesopolis | São Paulo | Brazil |  |  |  | X |  |  |  |  |  |
| *E. russatus* | MZUSP1844 |  | Boraceia | Salesopolis | São Paulo | Brazil |  |  |  | X |  |  |  |  |  |
| *E. russatus* | MZUSP1846 |  | Boraceia | Salesopolis | São Paulo | Brazil |  |  |  | X |  |  |  |  |  |
| *E. russatus* | MZUSP1894 |  | Boraceia | Salesopolis | São Paulo | Brazil |  |  |  | X |  |  |  |  |  |
| *E. russatus* | MZUSP1895 |  | Boraceia | Salesopolis | São Paulo | Brazil |  |  |  | X |  |  |  |  |  |
| *E. russatus* | MZUSP1912 |  | Boraceia | Salesopolis | São Paulo | Brazil |  |  |  | X |  |  |  |  |  |
| *E. russatus* | MZUSP20186 |  | Boraceia | Salesopolis | São Paulo | Brazil |  |  |  | X |  |  |  |  |  |
| *E. russatus* | MZUSP20540 |  | Boraceia | Salesopolis | São Paulo | Brazil |  |  |  | X |  |  |  |  |  |
| *E. russatus* | MZUSP20542 |  | Boraceia | Salesopolis | São Paulo | Brazil |  |  |  | X |  |  |  |  |  |
| *E. russatus* | MZUSP20546 |  | Boraceia | Salesopolis | São Paulo | Brazil |  |  |  | X |  |  |  |  |  |
| *E. russatus* | MZUSP20547 |  | Boraceia (Barragem Do Rio Do Campo) | Salesopolis | São Paulo | Brazil |  |  |  | X |  |  |  |  |  |
| *E. russatus* | MZUSP20548 |  | Boraceia | Salesopolis | São Paulo | Brazil |  |  |  | X |  |  |  |  |  |
| *E. russatus* | MZUSP20549 |  | Boraceia | Salesopolis | São Paulo | Brazil |  |  |  | X |  |  |  |  |  |
| *E. russatus* | MZUSP20550 |  | Boraceia | Salesopolis | São Paulo | Brazil |  |  |  | X |  |  |  |  |  |
| *E. russatus* | MZUSP20551 |  | Boraceia | Salesopolis | São Paulo | Brazil |  |  |  | X |  |  |  |  |  |
| *E. russatus* | MZUSP20552 |  | Casa Grande | Salesópolis | São Paulo | Brazil |  |  |  | X |  |  |  |  |  |
| *E. russatus* | MZUSP20553 |  | Casa Grande | Salesópolis | São Paulo | Brazil |  |  |  | X |  |  |  |  |  |
| *E. russatus* | MZUSP20554 |  | Casa Grande | Salesópolis | São Paulo | Brazil |  |  |  | X |  |  |  |  |  |
| *E. russatus* | MZUSP20555 |  | Casa Grande | Salesópolis | São Paulo | Brazil |  |  |  | X |  |  |  |  |  |
| *E. russatus* | MZUSP20556 |  | Casa Grande | Salesópolis | São Paulo | Brazil |  |  |  | X |  |  |  |  |  |
| *E. russatus* | MZUSP20557 |  | Casa Grande | Salesópolis | São Paulo | Brazil |  |  |  | X |  |  |  |  |  |
| *E. russatus* | MZUSP20558 |  | Casa Grande | Salesópolis | São Paulo | Brazil |  |  |  | X |  |  |  |  |  |
| *E. russatus* | MZUSP20559 |  | Casa Grande | Salesópolis | São Paulo | Brazil |  |  |  | X |  |  |  |  |  |
| *E. russatus* | MZUSP20560 |  | Casa Grande | Salesópolis | São Paulo | Brazil |  |  |  | X |  |  |  |  |  |
| *E. russatus* | MZUSP20561 |  | Casa Grande | Salesópolis | São Paulo | Brazil |  |  |  | X |  |  |  |  |  |
| *E. russatus* | MZUSP20563 |  | Casa Grande | Salesópolis | São Paulo | Brazil |  |  |  | X |  |  |  |  |  |
| *E. russatus* | MZUSP20564 |  | Casa Grande | Salesópolis | São Paulo | Brazil |  |  |  | X |  |  |  |  |  |
| *E. russatus* | MZUSP2091 |  | Casa Grande | Salesópolis | São Paulo | Brazil |  |  |  | X |  |  |  |  |  |
| *E. russatus* | MZUSP2092 |  | Casa Grande | Salesópolis | São Paulo | Brazil |  |  |  | X |  |  |  |  |  |
| *E. russatus* | MZUSP2093 |  | Casa Grande | Salesópolis | São Paulo | Brazil |  |  |  | X |  |  |  |  |  |
| *E. russatus* | MZUSP21117 |  | Casa Grande | Salesópolis | São Paulo | Brazil |  |  |  | X |  |  |  |  |  |
| *E. russatus* | MZUSP2147 |  | Casa Grande | Salesópolis | São Paulo | Brazil |  |  |  | X |  |  |  |  |  |
| *E. russatus* | MZUSP21883 |  | Casa Grande | Salesópolis | São Paulo | Brazil |  |  |  | X |  |  |  |  |  |
| *E. russatus* | MZUSP22807 |  | Casa Grande | Salesópolis | São Paulo | Brazil |  |  |  | X |  |  |  |  |  |
| *E. russatus* | MZUSP23935 |  | Casa Grande, Biritiba Mirim | Salesópolis | São Paulo | Brazil |  |  |  | X |  |  |  |  |  |
| *E. russatus* | MZUSP23936 |  | Casa Grande | Salesópolis | São Paulo | Brazil |  |  |  | X |  |  |  |  |  |
| *E. russatus* | MZUSP23937 |  | Casa Grande | Salesópolis | São Paulo | Brazil |  |  |  | X |  |  |  |  |  |
| *E. russatus* | MZUSP23950 |  | Casa Grande | Salesópolis | São Paulo | Brazil |  |  |  | X |  |  |  |  |  |
| *E. russatus* | MZUSP23951 |  | Casa Grande | Salesópolis | São Paulo | Brazil |  |  |  | X |  |  |  |  |  |
| *E. russatus* | MZUSP23955 |  | Casa Grande | Salesópolis | São Paulo | Brazil |  |  |  | X |  |  |  |  |  |
| *E. russatus* | MZUSP23956 |  | Casa Grande | Salesópolis | São Paulo | Brazil |  |  |  | X |  |  |  |  |  |
| *E. russatus* | MZUSP23957 |  | Casa Grande | Salesópolis | São Paulo | Brazil |  |  |  | X |  |  |  |  |  |
| *E. russatus* | MZUSP25400 |  | Casa Grande | Salesópolis | São Paulo | Brazil |  |  |  | X |  |  |  |  |  |
| *E. russatus* | MZUSP25676 |  | Casa Grande | Salesópolis | São Paulo | Brazil |  |  |  | X |  |  |  |  |  |
| *E. russatus* | MZUSP25681 |  | Casa Grande | Salesópolis | São Paulo | Brazil |  |  |  | X |  |  |  |  |  |
| *E. russatus* | MZUSP25692 |  | Casa Grande | Salesópolis | São Paulo | Brazil |  |  |  | X |  |  |  |  |  |
| *E. russatus* | MZUSP26773 |  | Casa Grande | Salesópolis | São Paulo | Brazil |  |  |  | X |  |  |  |  |  |
| *E. russatus* | MZUSP27277 |  | Casa Grande | Salesópolis | São Paulo | Brazil |  |  |  | X |  |  |  |  |  |
| *E. russatus* | MZUSP27278 |  | Casa Grande | Salesópolis | São Paulo | Brazil |  |  |  | X |  |  |  |  |  |
| *E. russatus* | MZUSP27279 |  | Casa Grande | Salesópolis | São Paulo | Brazil |  |  |  | X |  |  |  |  |  |
| *E. russatus* | MZUSP27280 |  | Casa Grande | Salesópolis | São Paulo | Brazil |  |  |  | X |  |  |  |  |  |
| *E. russatus* | MZUSP27281 |  | Casa Grande | Salesópolis | São Paulo | Brazil |  |  |  | X |  |  |  |  |  |
| *E. russatus* | MZUSP27282 |  | Casa Grande | Salesópolis | São Paulo | Brazil |  |  |  | X |  |  |  |  |  |
| *E. russatus* | MZUSP27283 |  | Casa Grande | Salesópolis | São Paulo | Brazil |  |  |  | X |  |  |  |  |  |
| *E. russatus* | MZUSP27284 |  | Casa Grande | Salesópolis | São Paulo | Brazil |  |  |  | X |  |  |  |  |  |
| *E. russatus* | MZUSP27285 |  | Casa Grande | Salesópolis | São Paulo | Brazil |  |  |  | X |  |  |  |  |  |
| *E. russatus* | MZUSP27286 |  | Casa Grande | Salesópolis | São Paulo | Brazil |  |  |  | X |  |  |  |  |  |
| *E. russatus* | MZUSP27469 |  | Casa Grande | Salesópolis | São Paulo | Brazil |  |  |  | X |  |  |  |  |  |
| *E. russatus* | MZUSP28356 |  | Casa Grande | Salesópolis | São Paulo | Brazil |  |  |  | X |  |  |  |  |  |
| ***E. russatus*** |  | **CIT09** | **Fazenda Intervales** | **Sete Barras** |  | **Brazil** | **-24.22** | **-48.08** | **98** |  | **X** | **MT118062** | **X** | **MT118087** | **This study** |
| ***E. russatus*** |  | **CIT77** | **Fazenda Intervales** | **Sete Barras** |  | **Brazil** | **-24.22** | **-48.08** | **98** |  | **X** | **MT118063** | **X** | **MT118088** | **This study** |
| *E. russatus* | MZUSP28369 |  | São Carlos | São Carlos | São Paulo | Brazil |  |  |  | X |  |  |  |  |  |
| *E. russatus* | MZUSP28372 |  | São Carlos | São Carlos | São Paulo | Brazil |  |  |  | X |  |  |  |  |  |
| *E. russatus* | MZUSP28373 |  | São Carlos | São Carlos | São Paulo | Brazil |  |  |  | X |  |  |  |  |  |
| ***E. russatus*** |  | **ROD139** | **Horto Zona Norte, São Paulo, São Paulo, Brazil** | **São Paulo** |  | **Brazil** | **-23.45** | **-46.63** | **99** |  | **X** | **MT118070** | **X** | **MT118094** | **This study** |
| *E. russatus* | MZUSP28374 |  | Teodoro Sampaio | Teodoro Sampaio | São Paulo | Brazil |  |  |  | X |  |  |  |  |  |
| *E. russatus* | MZUSP28375 |  | Serra D'Agua, Praia Dura | Ubatuba | São Paulo | Brazil |  |  |  | X |  |  |  |  |  |
| *E. russatus* | MZUSP28376 |  | Serra D'Agua | Ubatuba | São Paulo | Brazil |  |  |  | X |  |  |  |  |  |
| *E. russatus* | MZUSP28377 |  | Serra D'Agua, Praia Dura | Ubatuba | São Paulo | Brazil |  |  |  | X |  |  |  |  |  |
| *E. russatus* | MZUSP28382 |  | Serra D'Agua, Praia Dura | Ubatuba | São Paulo | Brazil |  |  |  | X |  |  |  |  |  |
| *E. russatus* | MZUSP28383 |  | Corrego Do Cemiterio | Ubatuba | São Paulo | Brazil |  |  |  | X |  |  |  |  |  |
| *E. russatus* | MZUSP28384 |  | Corrego Do Cemiterio | Ubatuba | São Paulo | Brazil |  |  |  | X |  |  |  |  |  |
| *E. russatus* | MZUSP28385 |  | Corrego Do Cemitério | Ubatuba | São Paulo | Brazil |  |  |  | X |  |  |  |  |  |
| *E. russatus* | MZUSP28387 |  | Estação Experimental | Ubatuba | São Paulo | Brazil |  |  |  | X |  |  |  |  |  |
| *E. russatus* | MZUSP28389 |  | Estação Experimental | Ubatuba | São Paulo | Brazil |  |  |  | X |  |  |  |  |  |
| *E. russatus* | MZUSP28390 |  | Estação Experimental | Ubatuba | São Paulo | Brazil |  |  |  | X |  |  |  |  |  |
| *E. russatus* | MZUSP28391 |  | Corrego Do Cemiterio | Ubatuba | São Paulo | Brazil |  |  |  | X |  |  |  |  |  |
| *E. russatus* | MZUSP28392 |  | Estação Experimental | Ubatuba | São Paulo | Brazil |  |  |  | X |  |  |  |  |  |
| *E. russatus* | MZUSP28393 |  | Corrego Do Cemitério | Ubatuba | São Paulo | Brazil |  |  |  | X |  |  |  |  |  |
| *E. russatus* | MZUSP28397 |  | Corrego Cachoeira Grande | Ubatuba | São Paulo | Brazil |  |  |  | X |  |  |  |  |  |
| *E. russatus* | MZUSP3205 |  | Corrego Cachoeira Grande | Ubatuba | São Paulo | Brazil |  |  |  | X |  |  |  |  |  |
| *E. russatus* | MZUSP3206 |  | Estação Experimental | Ubatuba | São Paulo | Brazil |  |  |  | X |  |  |  |  |  |
| *E. russatus* | MZUSP574 |  | Fazenda Capricornio, 5.5 Km N, 1.2 Km W Ubatuba | Ubatuba | São Paulo | Brazil |  |  |  | X |  |  |  |  |  |
| *E. russatus* | MZUSP59 |  | Fazenda Capricornio, 5.5 Km N, 1.2 Km W Ubatuba | Ubatuba | São Paulo | Brazil |  |  |  | X |  |  |  |  |  |
| *E. russatus* | MZUSP6160 |  | Ubatuba | Ubatuba | São Paulo | Brazil |  |  |  | X |  |  |  |  |  |
| *E. russatus* | MZUSP8203 |  | Ubatuba | Ubatuba | São Paulo | Brazil |  |  |  | X |  |  |  |  |  |
| *E. russatus* | MZUSP8204 |  | Ubatuba | Ubatuba | São Paulo | Brazil |  |  |  | X |  |  |  |  |  |
| *E. russatus* | MZUSP8206 |  | Ubatuba | Ubatuba | São Paulo | Brazil |  |  |  | X |  |  |  |  |  |
| *E. russatus* | MZUSP8208 |  | Ubatuba | Ubatuba | São Paulo | Brazil |  |  |  | X |  |  |  |  |  |
| *E. russatus* | MZUSP8209 |  | Ubatuba | Ubatuba | São Paulo | Brazil |  |  |  | X |  |  |  |  |  |
| *E. russatus* | MZUSP8210 |  | Ubatuba | Ubatuba | São Paulo | Brazil |  |  |  | X |  |  |  |  |  |
| *E. russatus* | MNRJ32764 |  | Sommerfeld Colony # Ii | Caaguazu |  | Paraguai |  |  |  | X |  |  |  |  |  |
| *E. russatus* | TX60660 |  | Estancia Santa Clara | Concepcion |  | Paraguai |  |  |  | X |  |  |  |  |  |
| *E. russatus* | LP16 |  |  |  |  |  |  |  |  | X |  |  |  |  |  |
| *E. russatus* | LP21 |  |  |  |  |  |  |  |  | X |  |  |  |  |  |
| ***Euryoryzomys* sp.** |  | **MTR046** | **Pacoti** | **Pacoti** |  | **Brazil** | **-4.21** | **-38.91** | **100** |  | **X** | **MT118072** | **X** | **MT118096** | **This study** |
| ***Euryoryzomys* sp.** |  | **MTR048** | **Pacoti** | **Pacoti** |  | **Brazil** | **-4.21** | **-38.91** | **10** |  | **X** | **MT118073** |  |  | **This study** |
