# Supplementary material for: A review of Euryoryzomys legatus (Rodentia, Sigmodontinae): morphological redescription, cytogenetics, and molecular phylogeny
Source: PeerJ. 2020 Oct 29;8:e9884. doi: 10.7717/peerj.9884 (PMC7603791; doi:10.7717/peerj.9884)
Supplement: Supplemental Information 8 — Taxon, gene, GenBank accession number, voucher/field number, and references are indicated. [file peerj-08-9884-s008.docx]

| **Taxon** | **Voucher Number** | **Cytb**  **GenBank Accession** | **COXI**  **GenBank Accession** | **Reference** |
| --- | --- | --- | --- | --- |
| *Handleyomys chapmani* | ROM YHM191 | KP778464 | JF491812 | Almendra et al., 2018; Engstrom et al., 2012 |
| *Handleyomys chapmani* | ROM:YHM223 | KP778338 | JF491800 | Almendra et al., 2018; Engstrom et al., 2012 |
| *Nephelomys devius* | ROM:97316 | KP778411 | JF491746 | Almendra et al., 2018; Engstrom et al., 2012 |
| *Nephelomys devius* | ROM:97301 | KP778209 | EU095459 | Almendra et al., 2018; Borisenko et al., 2008 |
| *Oecomys auyantepui* | ROM:114316 | KP778287 | JQ601049 | Almendra et al., 2018, International Barcode of Life (iBOL), 2012 |
| *Oecomys roberti* | MN71210 | KF815442 | KF815402 | Vilela et al., 2014 |
| *Hylaeamys megacephalus* | ROM:100908 | KP778270 | JF492135 | Almendra et al., 2018; Engstrom et al., 2012 |
| *Hylaeamys megacephalus* | MN7000 | KF815441 | KF815399 | Vilela et al., 2014 |
| *Wiedomys cerradensis* | MN:71267 | KF815445 | KF815395 | Vilela et al., 2014 |

**REFERENCES**

Almendra AL, González-Cózatl FX, Engstrom MD, Rogers DS. 2018. Evolutionary relationships and climatic niche evolution in the genus *Handleyomys* (Sigmodontinae: Oryzomyini). Molecular Phylogenetics and Evolution 128: 12–25. DOI 10.1016/j.ympev.2018.06.018.

Borisenko AV, Lim BK, Ivanova NV, Hanner RH, Hebert PDN. 2008. DNA barcoding in surveys of small mammal communities: a field study in Suriname. Molecular Ecology Resources 8(3): 471–479 DOI 10.1111/j.1471-8286.2007.01998.x.

Engstrom MD, Lim BK, Eger JL, Borisenko AV. 2012. ROM Mammals –Sigmodontinae. Unpublished.

International Barcode of Life (iBOL).2012. iBOL Data Release. Unpublished.

Vilela JF, Mello B, Voloch CM, Schrago CG. 2014. Sigmodontine rodents diversified in South American prior to the complete rise of the Panamanian Isthmus. Journal of Zoological Systematics and Evolutionary Research 52(3): 249–256. DOI 10.1111/jzs.12057.
