# Supplementary material for: A review of Euryoryzomys legatus (Rodentia, Sigmodontinae): morphological redescription, cytogenetics, and molecular phylogeny
Source: PeerJ. 2020 Oct 29;8:e9884. doi: 10.7717/peerj.9884 (PMC7603791; doi:10.7717/peerj.9884)
Supplement: Supplemental Information 9 [file peerj-08-9884-s009.docx]

|  |  | **1** | **2** | **3** | **4** | **5** | **6** | **7** | **8** | **9** |
| --- | --- | --- | --- | --- | --- | --- | --- | --- | --- | --- |
| **1** | ***E. legatus*** |  |  |  |  |  |  |  |  |  |
| **2** | ***E. nitidus* B** | 0.042 |  |  |  |  |  |  |  |  |
| **3** | **E. nitidus A** | 0.051 | 0.045 |  |  |  |  |  |  |  |
| **4** | ***E. lamia*** | 0.075 | 0.063 | 0.071 |  |  |  |  |  |  |
| **5** | ***Euryoryzomys* sp.** | 0.065 | 0.059 | 0.066 | 0.043 |  |  |  |  |  |
| **6** | ***E. russatus*** | 0.16 | 0.169 | 0.16 | 0.166 | 0.149 |  |  |  |  |
| **7** | ***E. emmonsae* B** | 0.142 | 0.141 | 0.141 | 0.143 | 0.131 | 0.145 |  |  |  |
| **8** | ***E. emmonsae* A** | 0.16 | 0.166 | 0.16 | 0.15 | 0.146 | 0.14 | 0.111 |  |  |
| **9** | ***E. macconnelli* B** | 0.15 | 0.157 | 0.158 | 0.165 | 0.146 | 0.151 | 0.15 | 0.147 |  |
| **10** | ***E. macconnelli* A** | 0.147 | 0.14 | 0.145 | 0.151 | 0.144 | 0.159 | 0.158 | 0.143 | 0.057 |
