# Supplementary material for: A review of Euryoryzomys legatus (Rodentia, Sigmodontinae): morphological redescription, cytogenetics, and molecular phylogeny
Source: PeerJ. 2020 Oct 29;8:e9884. doi: 10.7717/peerj.9884 (PMC7603791; doi:10.7717/peerj.9884)
Supplement: Supplemental Information 10 [file peerj-08-9884-s010.docx]

|  |  | **1** | **2** | **3** | **4** | **5** | **6** | **7** | **8** | **9** | **10** | **11** | **12** | **13** | **14** | **15** | **16** | **17** | **18** |
| --- | --- | --- | --- | --- | --- | --- | --- | --- | --- | --- | --- | --- | --- | --- | --- | --- | --- | --- | --- |
| **1** | ***E. legatus* JPJ2681 - Arroyo Yuto, Jujuy, Argentina (6)** | | | | |  |  |  |  |  |  |  |  |  |  |  |  |  |  |
| **2** | ***E. legatus* JPJ2682 - Arroyo Yuto, Jujuy, Argentina (6)** | 0.002 |  |  |  |  |  |  |  |  |  |  |  |  |  |  |  |  |  |
| **3** | ***E. legatus* JPJ1109 - Finca El Piquete, Jujuy, Argentina (7)** | 0.002 | 0 |  |  |  |  |  |  |  |  |  |  |  |  |  |  |  |  |
| **4** | ***E. legatus* JPJ1542 - Campo Largo, Salta, Argentina (17)** | 0.002 | 0 | 0 |  |  |  |  |  |  |  |  |  |  |  |  |  |  |  |
| **5** | ***E. legatus* MACN26339 - Finca Río Seco, Salta, Argentina (21)** | 0.002 | 0 | 0 | 0 |  |  |  |  |  |  |  |  |  |  |  |  |  |  |
| **6** | ***E. legatus* MACN27069 - Planta Caimancito, Jujuy, Argentina (10)** | 0.002 | 0 | 0 | 0 | 0 |  |  |  |  |  |  |  |  |  |  |  |  |  |
| **7** | ***E. nitidus* MSB70697 - Estancia Cuevaas, Santa Cruz, Bolivia (33)** | 0.019 | 0.017 | 0.017 | 0.017 | 0.017 | 0.017 |  |  |  |  |  |  |  |  |  |  |  |  |
| **8** | ***E. nitidus* MSB56057 - 45km N Yacuma, Beni, Bolivia** | 0.044 | 0.043 | 0.043 | 0.043 | 0.043 | 0.043 | 0.037 |  |  |  |  |  |  |  |  |  |  |  |
| **9** | ***E. nitidus* MSB57117 - Palmira, Pando, Bolivia (32)** | 0.044 | 0.043 | 0.043 | 0.043 | 0.043 | 0.043 | 0.041 | 0.017 |  |  |  |  |  |  |  |  |  |  |
| **10** | ***E. nitidus* CR97 - Reserva Cusco Amazónico, Cusco, Peru (36)** | 0.038 | 0.036 | 0.036 | 0.036 | 0.036 | 0.036 | 0.035 | 0.019 | 0.013 |  |  |  |  |  |  |  |  |  |
| **11** | ***E. nitidus* MVZ16627 - Reserva Cusco Amazónico, Cusco, Peru (36)** | 0.04 | 0.038 | 0.038 | 0.038 | 0.038 | 0.038 | 0.037 | 0.011 | 0.013 | 0.009 |  |  |  |  |  |  |  |  |
| **12** | ***E. nitidus* MVZ190456 - Igarapé-Porangaba, Acre, Brazil (34)** | 0.038 | 0.036 | 0.036 | 0.036 | 0.036 | 0.036 | 0.035 | 0.015 | 0.009 | 0.011 | 0.009 |  |  |  |  |  |  |  |
| **13** | ***E. nitidus* INPA3193 - Igarapé-Porangaba, Acre, Brazil (34)** | 0.038 | 0.036 | 0.036 | 0.036 | 0.036 | 0.036 | 0.035 | 0.015 | 0.009 | 0.011 | 0.009 | 0 |  |  |  |  |  |  |
| **14** | ***E. nitidus* M968409 - Apiacás, Mato Grosso, Brazil (35)** | 0.05 | 0.048 | 0.048 | 0.048 | 0.048 | 0.048 | 0.046 | 0.049 | 0.051 | 0.045 | 0.047 | 0.045 | 0.045 |  |  |  |  |  |
| **15** | ***E. nitidus* M97008 - Apiacás, Mato, Grosso (35)** | 0.05 | 0.048 | 0.048 | 0.048 | 0.048 | 0.048 | 0.046 | 0.049 | 0.051 | 0.045 | 0.047 | 0.045 | 0.045 | 0 |  |  |  |  |
| **16** | ***E. nitidus* APC153 - Juruena, Mato Grosso, Brazil (25)** | 0.057 | 0.055 | 0.055 | 0.055 | 0.055 | 0.055 | 0.053 | 0.055 | 0.057 | 0.051 | 0.053 | 0.051 | 0.051 | 0.015 | 0.015 |  |  |  |
| **17** | ***Euryoryzomys* sp. MTR046 - Pacoti, Ceará, Brazil (43)** | 0.067 | 0.065 | 0.065 | 0.065 | 0.065 | 0.065 | 0.067 | 0.065 | 0.071 | 0.065 | 0.063 | 0.063 | 0.063 | 0.059 | 0.059 | 0.059 |  |  |
| **18** | ***Euryozyomys* sp. MTR048 - Pacoti, Ceará, Brazil (43)** | 0.067 | 0.065 | 0.065 | 0.065 | 0.065 | 0.065 | 0.067 | 0.065 | 0.071 | 0.065 | 0.063 | 0.063 | 0.063 | 0.059 | 0.059 | 0.059 | 0 |  |
| **19** | ***E. lamia* CRB968 - Cavalcante, Goiás, Brazil (5)** | 0.075 | 0.073 | 0.073 | 0.073 | 0.073 | 0.073 | 0.071 | 0.076 | 0.073 | 0.067 | 0.073 | 0.071 | 0.071 | 0.065 | 0.065 | 0.057 | 0.041 | 0.041 |
| **20** | ***E. lamia* CRB983 - Cavalcante, Goiás, Brazil (5)** | 0.075 | 0.073 | 0.073 | 0.073 | 0.073 | 0.073 | 0.071 | 0.076 | 0.073 | 0.067 | 0.073 | 0.071 | 0.071 | 0.065 | 0.065 | 0.057 | 0.041 | 0.041 |
| **21** | ***E. russatus* CIT77 - Fazenda Intervales, São Paulo, Brazil (40)** | 0.164 | 0.162 | 0.162 | 0.162 | 0.162 | 0.162 | 0.159 | 0.16 | 0.16 | 0.155 | 0.167 | 0.157 | 0.157 | 0.17 | 0.17 | 0.172 | 0.152 | 0.152 |
| **22** | ***E. russatus* CIT1796- Juquitiba, São Paulo, Brazil** | 0.164 | 0.162 | 0.162 | 0.162 | 0.162 | 0.162 | 0.159 | 0.162 | 0.162 | 0.158 | 0.17 | 0.165 | 0.165 | 0.168 | 0.168 | 0.17 | 0.145 | 0.145 |
| **23** | ***E. russatus* UNIBAN2022 - Biritiba Mirim, São Paulo, Brazil (39)** | 0.159 | 0.157 | 0.157 | 0.157 | 0.157 | 0.157 | 0.154 | 0.157 | 0.157 | 0.153 | 0.165 | 0.16 | 0.16 | 0.163 | 0.163 | 0.165 | 0.145 | 0.145 |
| **24** | ***E. russatus* CIT1755 - Biritiba-Mirim, São Paulo (39)** | 0.162 | 0.16 | 0.16 | 0.16 | 0.16 | 0.16 | 0.157 | 0.16 | 0.16 | 0.155 | 0.167 | 0.162 | 0.162 | 0.165 | 0.165 | 0.168 | 0.143 | 0.143 |
| **25** | ***E. russatus* ROD139 - Horto Zona Norte, São Paulo, Brazil (41)** | 0.162 | 0.16 | 0.16 | 0.16 | 0.16 | 0.16 | 0.157 | 0.16 | 0.16 | 0.155 | 0.167 | 0.162 | 0.162 | 0.165 | 0.165 | 0.168 | 0.143 | 0.143 |
| **26** | ***E. russatus* IIM298 - Wenceslau Brás, Paraná, Brazil (38)** | 0.164 | 0.162 | 0.162 | 0.162 | 0.162 | 0.162 | 0.159 | 0.157 | 0.157 | 0.153 | 0.165 | 0.155 | 0.155 | 0.17 | 0.17 | 0.173 | 0.153 | 0.153 |
| **27** | ***E. russatus* CIT09 - Fazenda Intervavles, São Paulo, Brazil (40)** | 0.162 | 0.16 | 0.16 | 0.16 | 0.16 | 0.16 | 0.157 | 0.155 | 0.155 | 0.15 | 0.162 | 0.152 | 0.152 | 0.168 | 0.168 | 0.17 | 0.15 | 0.15 |
| **28** | ***E. russatus* IIM300 - Wenceslau Brás, Paraná, Brazil (38)** | 0.159 | 0.157 | 0.157 | 0.157 | 0.157 | 0.157 | 0.154 | 0.153 | 0.153 | 0.148 | 0.16 | 0.15 | 0.15 | 0.165 | 0.165 | 0.168 | 0.148 | 0.148 |
| **29** | ***E. russatus* IIM130 - Ortigueira, Paraná, Brazil (37)** | 0.159 | 0.157 | 0.157 | 0.157 | 0.157 | 0.157 | 0.159 | 0.157 | 0.157 | 0.153 | 0.165 | 0.155 | 0.155 | 0.17 | 0.17 | 0.173 | 0.153 | 0.153 |
| **30** | ***E. russatus* IIM076 - Ortigueira, Paraná, Brazil (37)** | 0.162 | 0.16 | 0.16 | 0.16 | 0.16 | 0.16 | 0.157 | 0.155 | 0.155 | 0.15 | 0.162 | 0.152 | 0.152 | 0.168 | 0.168 | 0.17 | 0.15 | 0.15 |
| **31** | ***E. emmonsae* M97018 - Claudia, Mato Grosso, Brazil (1)** | 0.143 | 0.14 | 0.14 | 0.14 | 0.14 | 0.14 | 0.136 | 0.145 | 0.138 | 0.136 | 0.143 | 0.133 | 0.133 | 0.144 | 0.144 | 0.134 | 0.129 | 0.129 |
| **32** | ***E. emmonsae* M97120 - Claudia, Mato Grosso, Brazil (1)** | 0.143 | 0.14 | 0.14 | 0.14 | 0.14 | 0.14 | 0.136 | 0.145 | 0.138 | 0.136 | 0.143 | 0.133 | 0.133 | 0.144 | 0.144 | 0.134 | 0.129 | 0.129 |
| **33** | ***E. emmonsae* CS37 - Altamira, Pará, Brazil (4)** | 0.156 | 0.154 | 0.154 | 0.154 | 0.154 | 0.154 | 0.152 | 0.152 | 0.15 | 0.154 | 0.154 | 0.157 | 0.157 | 0.157 | 0.157 | 0.162 | 0.141 | 0.141 |
| **34** | ***E. emmonsae* MZUSP27150 - Altamira, Pará. Brazil (4)** | 0.159 | 0.157 | 0.157 | 0.157 | 0.157 | 0.157 | 0.154 | 0.154 | 0.152 | 0.157 | 0.157 | 0.159 | 0.159 | 0.16 | 0.16 | 0.165 | 0.143 | 0.143 |
| **35** | ***E. emmonsae* USNM549552 - Altamira, Pará, Brazil (4)** | 0.159 | 0.157 | 0.157 | 0.157 | 0.157 | 0.157 | 0.154 | 0.154 | 0.152 | 0.157 | 0.157 | 0.159 | 0.159 | 0.16 | 0.16 | 0.165 | 0.143 | 0.143 |
| **36** | ***E. emmonsae* APC312 - Vila Rica, Mato Grosso, Brazil (2)** | 0.164 | 0.161 | 0.161 | 0.161 | 0.161 | 0.161 | 0.159 | 0.159 | 0.157 | 0.162 | 0.162 | 0.164 | 0.164 | 0.165 | 0.165 | 0.17 | 0.148 | 0.148 |
| **37** | ***E. emmonsae* APC318 - Vila Rica, Mato Grosso, Brazil (2)** | 0.164 | 0.161 | 0.161 | 0.161 | 0.161 | 0.161 | 0.159 | 0.159 | 0.157 | 0.162 | 0.162 | 0.164 | 0.164 | 0.165 | 0.165 | 0.17 | 0.148 | 0.148 |
| **38** | ***E. macconnelli* M000147 - Juruena, Mato Grosso, Brazil (25)** | 0.148 | 0.145 | 0.145 | 0.145 | 0.145 | 0.145 | 0.145 | 0.153 | 0.143 | 0.148 | 0.15 | 0.144 | 0.144 | 0.136 | 0.136 | 0.138 | 0.143 | 0.143 |
| **39** | **E. macconnelli PEU960004 - Aripuanã, Mato Grosso, Brazil** | 0.152 | 0.15 | 0.15 | 0.15 | 0.15 | 0.15 | 0.15 | 0.158 | 0.148 | 0.148 | 0.15 | 0.148 | 0.148 | 0.143 | 0.143 | 0.145 | 0.148 | 0.148 |
| **40** | ***E. macconnelli* CS32 - Marabá, Pará, Brazil (4)** | 0.145 | 0.143 | 0.143 | 0.143 | 0.143 | 0.143 | 0.143 | 0.146 | 0.136 | 0.141 | 0.143 | 0.134 | 0.134 | 0.134 | 0.134 | 0.141 | 0.141 | 0.141 |
| **41** | ***E. macconnelli* AMNH272678 - Rio Gálvez, Loreto, Peru (28)** | 0.147 | 0.145 | 0.145 | 0.145 | 0.145 | 0.145 | 0.155 | 0.163 | 0.153 | 0.157 | 0.16 | 0.153 | 0.153 | 0.15 | 0.15 | 0.157 | 0.138 | 0.138 |
| **42** | ***E. macconnelli* RSV2025 - Nuevo San Juan, Loreto, Peru (27)** | 0.148 | 0.145 | 0.145 | 0.145 | 0.145 | 0.145 | 0.155 | 0.163 | 0.153 | 0.158 | 0.16 | 0.153 | 0.153 | 0.15 | 0.15 | 0.157 | 0.145 | 0.145 |
| **43** | ***E. macconnelli* RSV2030 - Nuevo San Juan, Loreto, Peru (27)** | 0.148 | 0.145 | 0.145 | 0.145 | 0.145 | 0.145 | 0.155 | 0.163 | 0.153 | 0.158 | 0.16 | 0.153 | 0.153 | 0.15 | 0.15 | 0.157 | 0.141 | 0.141 |
| **44** | ***E. macconnelli* LLW462 - Tangoshiari, Loreto, Peru (29)** | 0.152 | 0.15 | 0.15 | 0.15 | 0.15 | 0.15 | 0.16 | 0.163 | 0.153 | 0.163 | 0.16 | 0.153 | 0.153 | 0.155 | 0.155 | 0.157 | 0.145 | 0.145 |
| **45** | ***E. macconnelli* LLW447 - Tangoshiari, Loreto, Peru (29)** | 0.155 | 0.152 | 0.152 | 0.152 | 0.152 | 0.152 | 0.162 | 0.165 | 0.155 | 0.165 | 0.162 | 0.155 | 0.155 | 0.157 | 0.157 | 0.16 | 0.15 | 0.15 |
| **46** | ***E. macconnelli* CMNH64561 - Rudi Kappel Vliegveld, Brokopondo, Suriname** | 0.165 | 0.163 | 0.163 | 0.163 | 0.163 | 0.163 | 0.162 | 0.161 | 0.153 | 0.16 | 0.165 | 0.158 | 0.158 | 0.172 | 0.172 | 0.17 | 0.15 | 0.15 |
|  |  |  |  |  |  |  |  |  |  |  |  |  |  |  |  |  |  |  |  |

|  |  | **19** | **20** | **21** | **22** | **23** | **24** | **25** | **26** | **27** | **28** | **29** | **30** | **31** | **32** | **33** | **34** | **35** | **36** | **37** | **38** | **39** | **40** | **41** | **42** | **43** | **44** | **45** |
| --- | --- | --- | --- | --- | --- | --- | --- | --- | --- | --- | --- | --- | --- | --- | --- | --- | --- | --- | --- | --- | --- | --- | --- | --- | --- | --- | --- | --- |
| **19** | ***E. lamia* CRB968 - Cavalcante, Goiás, Brazil (5)** |  |  |  |  |  |  |  |  |  |  |  |  |  |  |  |  |  |  |  |  |  |  |  |  |  |  |  |
| **20** | ***E. lamia* CRB983 - Cavalcante, Goiás, Brazil (5)** | 0 |  |  |  |  |  |  |  |  |  |  |  |  |  |  |  |  |  |  |  |  |  |  |  |  |  |  |
| **21** | ***E. russatus* CIT77 - Fazenda Intervales, São Paulo, Brazil (40)** | 0.169 | 0.169 |  |  |  |  |  |  |  |  |  |  |  |  |  |  |  |  |  |  |  |  |  |  |  |  |  |
| **22** | ***E. russatus* CIT1796- Juquitiba, São Paulo, Brazil** | 0.162 | 0.162 | 0.029 |  |  |  |  |  |  |  |  |  |  |  |  |  |  |  |  |  |  |  |  |  |  |  |  |
| **23** | ***E. russatus* UNIBAN2022 - Biritiba Mirim, São Paulo, Brazil (39)** | 0.157 | 0.157 | 0.029 | 0.008 |  |  |  |  |  |  |  |  |  |  |  |  |  |  |  |  |  |  |  |  |  |  |  |
| **24** | ***E. russatus* CIT1755 - Biritiba-Mirim, São Paulo (39)** | 0.16 | 0.16 | 0.027 | 0.006 | 0.002 |  |  |  |  |  |  |  |  |  |  |  |  |  |  |  |  |  |  |  |  |  |  |
| **25** | ***E. russatus* ROD139 - Horto Zona Norte, São Paulo, Brazil (41)** | 0.16 | 0.16 | 0.029 | 0.008 | 0.004 | 0.002 |  |  |  |  |  |  |  |  |  |  |  |  |  |  |  |  |  |  |  |  |  |
| **26** | ***E. russatus* IIM298 - Wenceslau Brás, Paraná, Brazil (38)** | 0.17 | 0.17 | 0.008 | 0.029 | 0.029 | 0.027 | 0.025 |  |  |  |  |  |  |  |  |  |  |  |  |  |  |  |  |  |  |  |  |
| **27** | ***E. russatus* CIT09 - Fazenda Intervavles, São Paulo, Brazil (40)** | 0.167 | 0.167 | 0.004 | 0.025 | 0.025 | 0.023 | 0.025 | 0.004 |  |  |  |  |  |  |  |  |  |  |  |  |  |  |  |  |  |  |  |
| **28** | ***E. russatus* IIM300 - Wenceslau Brás, Paraná, Brazil (38)** | 0.165 | 0.165 | 0.006 | 0.027 | 0.027 | 0.025 | 0.027 | 0.006 | 0.002 |  |  |  |  |  |  |  |  |  |  |  |  |  |  |  |  |  |  |
| **29** | ***E. russatus* IIM130 - Ortigueira, Paraná, Brazil (37)** | 0.17 | 0.17 | 0.006 | 0.027 | 0.027 | 0.025 | 0.027 | 0.006 | 0.002 | 0.004 |  |  |  |  |  |  |  |  |  |  |  |  |  |  |  |  |  |
| **30** | ***E. russatus* IIM076 - Ortigueira, Paraná, Brazil (37)** | 0.167 | 0.167 | 0.004 | 0.025 | 0.025 | 0.023 | 0.025 | 0.004 | 0 | 0.002 | 0.002 |  |  |  |  |  |  |  |  |  |  |  |  |  |  |  |  |
| **31** | ***E. emmonsae* M97018 - Claudia, Mato Grosso, Brazil (1)** | 0.141 | 0.141 | 0.143 | 0.15 | 0.145 | 0.148 | 0.145 | 0.141 | 0.141 | 0.138 | 0.141 | 0.141 |  |  |  |  |  |  |  |  |  |  |  |  |  |  |  |
| **32** | ***E. emmonsae* M97120 - Claudia, Mato Grosso, Brazil (1)** | 0.141 | 0.141 | 0.143 | 0.15 | 0.145 | 0.148 | 0.145 | 0.141 | 0.141 | 0.138 | 0.141 | 0.141 | 0 |  |  |  |  |  |  |  |  |  |  |  |  |  |  |
| **33** | ***E. emmonsae* CS37 - Altamira, Pará, Brazil (4)** | 0.148 | 0.148 | 0.129 | 0.137 | 0.132 | 0.134 | 0.137 | 0.132 | 0.127 | 0.125 | 0.13 | 0.127 | 0.108 | 0.108 |  |  |  |  |  |  |  |  |  |  |  |  |  |
| **34** | ***E. emmonsae* MZUSP27150 - Altamira, Pará. Brazil (4)** | 0.145 | 0.145 | 0.139 | 0.146 | 0.142 | 0.144 | 0.146 | 0.142 | 0.137 | 0.134 | 0.139 | 0.137 | 0.108 | 0.108 | 0.008 |  |  |  |  |  |  |  |  |  |  |  |  |
| **35** | ***E. emmonsae* USNM549552 - Altamira, Pará, Brazil (4)** | 0.145 | 0.145 | 0.139 | 0.146 | 0.142 | 0.144 | 0.146 | 0.142 | 0.137 | 0.134 | 0.139 | 0.137 | 0.108 | 0.108 | 0.008 | 0 |  |  |  |  |  |  |  |  |  |  |  |
| **36** | ***E. emmonsae* APC312 - Vila Rica, Mato Grosso, Brazil (2)** | 0.15 | 0.15 | 0.136 | 0.144 | 0.139 | 0.142 | 0.144 | 0.139 | 0.134 | 0.132 | 0.137 | 0.134 | 0.11 | 0.11 | 0.006 | 0.006 | 0.006 |  |  |  |  |  |  |  |  |  |  |
| **37** | ***E. emmonsae* APC318 - Vila Rica, Mato Grosso, Brazil (2)** | 0.15 | 0.15 | 0.136 | 0.144 | 0.139 | 0.142 | 0.144 | 0.139 | 0.134 | 0.132 | 0.137 | 0.134 | 0.11 | 0.11 | 0.006 | 0.006 | 0.006 | 0 |  |  |  |  |  |  |  |  |  |
| **38** | ***E. macconnelli* M000147 - Juruena, Mato Grosso, Brazil (25)** | 0.146 | 0.146 | 0.161 | 0.159 | 0.154 | 0.157 | 0.157 | 0.162 | 0.159 | 0.157 | 0.162 | 0.159 | 0.157 | 0.157 | 0.138 | 0.138 | 0.138 | 0.146 | 0.146 |  |  |  |  |  |  |  |  |
| **39** | **E. macconnelli PEU960004 - Aripuanã, Mato Grosso, Brazil** | 0.15 | 0.15 | 0.166 | 0.164 | 0.159 | 0.161 | 0.161 | 0.166 | 0.164 | 0.161 | 0.166 | 0.164 | 0.16 | 0.16 | 0.136 | 0.136 | 0.136 | 0.143 | 0.143 | 0.011 |  |  |  |  |  |  |  |
| **40** | ***E. macconnelli* CS32 - Marabá, Pará, Brazil (4)** | 0.153 | 0.153 | 0.159 | 0.15 | 0.145 | 0.148 | 0.148 | 0.157 | 0.155 | 0.152 | 0.157 | 0.155 | 0.151 | 0.151 | 0.139 | 0.139 | 0.139 | 0.146 | 0.146 | 0.033 | 0.031 |  |  |  |  |  |  |
| **41** | ***E. macconnelli* AMNH272678 - Rio Gálvez, Loreto, Peru (28)** | 0.158 | 0.158 | 0.15 | 0.143 | 0.138 | 0.141 | 0.141 | 0.15 | 0.148 | 0.145 | 0.15 | 0.148 | 0.144 | 0.144 | 0.141 | 0.141 | 0.141 | 0.149 | 0.149 | 0.053 | 0.057 | 0.057 |  |  |  |  |  |
| **42** | ***E. macconnelli* RSV2025 - Nuevo San Juan, Loreto, Peru (27)** | 0.165 | 0.165 | 0.152 | 0.145 | 0.141 | 0.143 | 0.143 | 0.153 | 0.15 | 0.148 | 0.153 | 0.15 | 0.146 | 0.146 | 0.144 | 0.144 | 0.144 | 0.151 | 0.151 | 0.046 | 0.05 | 0.051 | 0.006 |  |  |  |  |
| **43** | ***E. macconnelli* RSV2030 - Nuevo San Juan, Loreto, Peru (27)** | 0.16 | 0.16 | 0.147 | 0.141 | 0.136 | 0.138 | 0.138 | 0.148 | 0.145 | 0.143 | 0.148 | 0.145 | 0.141 | 0.141 | 0.139 | 0.139 | 0.139 | 0.146 | 0.146 | 0.051 | 0.055 | 0.055 | 0.002 | 0.004 |  |  |  |
| **44** | ***E. macconnelli* LLW462 - Tangoshiari, Loreto, Peru (29)** | 0.165 | 0.165 | 0.157 | 0.155 | 0.15 | 0.153 | 0.153 | 0.157 | 0.155 | 0.153 | 0.157 | 0.155 | 0.151 | 0.151 | 0.146 | 0.146 | 0.146 | 0.154 | 0.154 | 0.051 | 0.055 | 0.063 | 0.017 | 0.015 | 0.015 |  |  |
| **45** | ***E. macconnelli* LLW447 - Tangoshiari, Loreto, Peru (29)** | 0.17 | 0.17 | 0.16 | 0.158 | 0.153 | 0.155 | 0.155 | 0.16 | 0.158 | 0.155 | 0.16 | 0.158 | 0.158 | 0.158 | 0.153 | 0.153 | 0.153 | 0.161 | 0.161 | 0.057 | 0.061 | 0.069 | 0.021 | 0.021 | 0.021 | 0.006 |  |
| **46** | ***E. macconnelli* CMNH64561 - Rudi Kappel Vliegveld, Brokopondo, Suriname** | 0.158 | 0.158 | 0.145 | 0.14 | 0.136 | 0.138 | 0.138 | 0.14 | 0.143 | 0.145 | 0.145 | 0.143 | 0.143 | 0.143 | 0.134 | 0.139 | 0.139 | 0.137 | 0.137 | 0.131 | 0.129 | 0.132 | 0.138 | 0.141 | 0.136 | 0.146 | 0.15 |
