# Supplementary material for: A review of Euryoryzomys legatus (Rodentia, Sigmodontinae): morphological redescription, cytogenetics, and molecular phylogeny
Source: PeerJ. 2020 Oct 29;8:e9884. doi: 10.7717/peerj.9884 (PMC7603791; doi:10.7717/peerj.9884)
Supplement: Supplemental Information 11 — Loadings of the variables, eigenvalues, and proportion of the variance explained for the first 3 principal components (PC). Results are based on log10- transformed craniodental variables. See “Material & Methods” for variable abbreviations. [file peerj-08-9884-s011.docx]

|  | Eigenvectors | | | | | |
| --- | --- | --- | --- | --- | --- | --- |
|  |  | PC 1 |  | PC 2 |  | PC 3 |
| CIL |  | 0.25125 |  | 0.090133 |  | 0.077838 |
| DL |  | 0.17469 |  | 0.39591 |  | 0.27776 |
| PB |  | 0.31198 |  | -0.074567 |  | 0.30016 |
| MTRL |  | 0.1374 |  | -0.18473 |  | 0.1122 |
| BLLT |  | 0.17458 |  | -0.24239 |  | 0.19793 |
| IFL |  | 0.010905 |  | 0.68031 |  | 0.12959 |
| AW1 |  | 0.18003 |  | -0.010013 |  | 0.072429 |
| ZB |  | 0.30715 |  | -0.036846 |  | -0.03477 |
| ZP |  | 0.42619 |  | 0.099105 |  | -0.82921 |
| BB |  | 0.2286 |  | -0.23487 |  | 0.035621 |
| IOC |  | 0.24642 |  | -0.3643 |  | 0.092174 |
| RW2 |  | 0.32411 |  | 0.11539 |  | 0.077855 |
| RL |  | 0.32679 |  | 0.057898 |  | 0.13116 |
| OL |  | 0.2351 |  | 0.12277 |  | -0.0016914 |
| OCW |  | 0.16974 |  | -0.098083 |  | 0.16816 |
| ML |  | 0.20556 |  | 0.18266 |  | 0.052726 |
| Eigenvalue |  | 0.00683981 |  | 0.00172527 |  | 0.000602453 |
| % of the variance |  | 59.629 |  | 15.041 |  | 5.2522 |
