# Supplementary material for: A review of Euryoryzomys legatus (Rodentia, Sigmodontinae): morphological redescription, cytogenetics, and molecular phylogeny
Source: PeerJ. 2020 Oct 29;8:e9884. doi: 10.7717/peerj.9884 (PMC7603791; doi:10.7717/peerj.9884)
Supplement: Supplemental Information 12 — Loadings of the variables, eigenvalues, and proportion of the variance explained for the first 3 principal components (PC). Results are based on Mosimann shape craniodental variables. See “Material & Methods” for variable abbreviations. [file peerj-08-9884-s012.docx]

|  | Eigenvectors | | | | | |
| --- | --- | --- | --- | --- | --- | --- |
|  |  | PC 1 |  | PC 2 |  | PC 3 |
| CIL |  | -0.10891 |  | 0.048904 |  | -0.047285 |
| DL |  | -0.33951 |  | -0.24832 |  | -0.022577 |
| PB |  | 0.069825 |  | 0.14695 |  | -0.11128 |
| MTRL |  | 0.33169 |  | -0.14544 |  | -0.0092972 |
| BLLT |  | 0.25943 |  | -0.025617 |  | 0.79754 |
| IFL |  | -0.32733 |  | -0.6417 |  | 0.083227 |
| AW1 |  | 0.11897 |  | -0.15181 |  | -0.1232 |
| ZB |  | -0.002002 |  | 0.17196 |  | -0.24696 |
| ZP |  | -0.47442 |  | 0.56039 |  | 0.34157 |
| BB |  | 0.31525 |  | 0.12773 |  | -0.11971 |
| IOC |  | 0.3646 |  | 0.12792 |  | -0.013059 |
| RW2 |  | -0.076431 |  | 0.1314 |  | -0.3339 |
| RL |  | -0.16426 |  | 0.17914 |  | -0.13185 |
| OL |  | -0.08275 |  | -0.03374 |  | -0.051551 |
| OCW |  | 0.23902 |  | -0.11289 |  | -0.043611 |
| ML |  | -0.12318 |  | -0.13488 |  | 0.031943 |
| Eigenvalue |  | 0.00391396 |  | 0.0015718 |  | 0.000526655 |
| % of the variance |  | 48.645 |  | 19.535 |  | 6.5456 |
