# Supplementary material for: A review of Euryoryzomys legatus (Rodentia, Sigmodontinae): morphological redescription, cytogenetics, and molecular phylogeny
Source: PeerJ. 2020 Oct 29;8:e9884. doi: 10.7717/peerj.9884 (PMC7603791; doi:10.7717/peerj.9884)
Supplement: Supplemental Information 13 — Loadings of the variables, eigenvalues, and proportion of the variance explained for the discriminant functions (DF). Results are based on Mosimann shape craniodental variables. See “Material & Methods” for variable abbreviations. [file peerj-08-9884-s013.docx]

|  | Eigenvector | |
| --- | --- | --- |
|  |  | DF 1 |
| CIL |  | 23.315 |
| DL |  | -37.499 |
| PB |  | 3.0807 |
| MTRL |  | -12.556 |
| BLLT |  | 12.268 |
| IFL |  | -23.847 |
| AW1 |  | -25.319 |
| ZB |  | 33.427 |
| ZP |  | 0.50073 |
| BB |  | 4.0298 |
| IOC |  | 24.454 |
| RW2 |  | -5.3651 |
| RL |  | 28.512 |
| OL |  | -10.801 |
| OCW |  | -11.281 |
| ML |  | -2.9183 |
| Eigenvalue |  | 4.4343 |
| % of the variance |  | 100 |
