# Supplementary material for: A review of Euryoryzomys legatus (Rodentia, Sigmodontinae): morphological redescription, cytogenetics, and molecular phylogeny
Source: PeerJ. 2020 Oct 29;8:e9884. doi: 10.7717/peerj.9884 (PMC7603791; doi:10.7717/peerj.9884)
Supplement: Supplemental Information 14 — Proportion of correct classifications = 95.7. [file peerj-08-9884-s014.docx]

|  |  | *E. legatus* |  | *E. nitidus* |  | Total |
| --- | --- | --- | --- | --- | --- | --- |
| *E. legatus* |  | 120 |  | 5 |  | 125 |
| *E. nitidus* |  | 5 |  | 104 |  | 109 |
| Total |  | 125 |  | 109 |  | 234 |
